# Phytobiotics in poultry: revolutionizing broiler chicken nutrition with plant-derived gut health enhancers

**DOI:** 10.1186/s40104-024-01101-9

**Published:** 2024-12-09

**Authors:** Uchechukwu Edna Obianwuna, Xinyu Chang, Vivian U. Oleforuh-Okoleh, Patience N. Onu, Haijun Zhang, Kai Qiu, Shugeng Wu

**Affiliations:** 1 National Engineering Research Center of Biological Feed, Institute of Feed Research, Chinese Academy of Agricultural Sciences, Beijing, 100081 China; 2 Department of Animal Science, Rivers State University, Port Harcourt, Rivers State Nigeria; 3 Department of Animal Science, Ebonyi State University, Abakiliki, Ebonyi State Nigeria

**Keywords:** Broilers, Essential oils, Gut health, Herbs and spices, Phytobiotics, Plant extracts

## Abstract

As the global population continues to expand, the demand for broiler chicken production to supply safe and high-quality meat is increasing. To meet this ever-growing demand, broiler chickens with enhanced growth performance are being developed, but they often face challenges related to oxidative stress, which can adversely affect gut health. Phytobiotics, which are plant-derived feed additives known for their antimicrobial, antioxidant, immune-modulating, and growth-promoting properties, have emerged as promising natural alternatives to synthetic antibiotics. This review consolidates recent advancements in the use of phytobiotics-derived products from leaves, roots, seeds, flowers, and their extracts in broiler diets reared under standard experimental conditions, without the introduction of stressors. The focus is on elucidating the key mechanisms through which phytobiotics improve gut health, including their effects on gut morphology, integrity, microflora composition, antioxidant capacity, and immune function. The review highlights the potential of phytobiotics to revolutionize broiler nutrition by acting as natural enhancers of gut health. Research findings reveal that phytobiotics significantly improve intestinal health, and boost growth performance, offering a sustainable approach to managing to gut dysfunction. These findings indicate a potential shift in how gut-health related challenges in broilers can be addressed, moving towards natural phytobiotic therapy. However, several challenges persist. Optimizing the dosage of phytobiotics, ensuring consistent performance, and overcoming the limitations related to their extraction and application are key areas requiring further investigation. The review emphasizes the importance of continued research to refine phytobiotic formulations, explore synergistic effects, and incorporate advanced technologies such as AI-driven methods and precision nutrition to tailor feeding strategies more effectively. Additionally, the development of innovative delivery systems, such as nanoencapsulation, is suggested as a way to enhance the effectiveness and reliability of phytobiotics. By highlighting the potential of phytobiotics to revolutionize broiler nutrition, this review supports the poultry industry’s shift towards antibiotic-free and sustainable dietary solutions, offering new perspectives on the future of broiler chicken production.

**Supplementary Information:**

The online version contains supplementary material available at 10.1186/s40104-024-01101-9.

## Background

The use of antibiotics as growth promoters (AGPs) and gut enhancers in broiler production has been a long-standing practice due to their effectiveness in enhancing growth rates, improving feed efficiency, and positively influencing gut microbiota and innate immunity: these benefits collectively contribute to better overall health and performance in poultry [1]. However, the benefits of using antibiotics are increasingly overshadowed by the risks associated with drug residues in animal products [2]. For instance, antibiotics like tetracycline and amphenicol have been detected in food products, posing a risk to consumer health [3]. Additionally, the study by Hur et al. [4] found that isolates of *Salmonella enterica* from eggs and chicken carcasses were resistant to multiple antibiotics, including penicillins, sulfisoxazole, streptomycin, tetracycline, and quinolones. This resistance not only compromises poultry health but also increases the risk of secondary contamination in animal products, thereby threatening public health. Given the growing concern over antibiotic resistance and residue issues, there is a shift in broiler production towards eliminating antibiotics as growth promoters and gut enhancers. More recently, a study by Iwnski et al. [5] demonstrated that a phytogenic blend could effectively inhibit antibiotic-resistant strains of *Salmonella enterica *subsp, including enterica serovars, Enteritidis, Typhimurium, and Kentucky, suggesting the efficacy of its microbial effect. This shift emphasizes the need for alternative natural strategies that can maintain animal health and performance without contributing to antibiotic resistance.

In modern-day poultry production, broiler chickens are selectively bred for rapid growth and increased breast meat mass, which in turn leads to a high metabolic rate [6]. This intense selection process, coupled with intensive production systems, expose these birds to various stressors including prolonged photoperiods, high dust and ammonia levels, and pathogens load; these factors collectively induce oxidative stress [7]. Consequently, the production of reactive oxygen species (ROS) is accelerated, disrupting the antioxidant balance, impairing gut health, and ultimately affecting overall performance, leading to significant economic losses [8]. Effective gut health management is, therefore, crucial to sustaining broiler health and performance, this can be achieved by upregulating the gut antioxidant system, which supports intestinal integrity and barrier function [9], maintaining the intestinal mucosal barrier and controlling inflammatory responses which are vital in preventing pathogen invasion [10]. These benefits can enhance nutrient utilization by reducing nutrient competition, prevention of infection in the intestinal tract and increased nutrient bioavailability [11]. These challenges underscore the urgent need for effective alternatives that can maintain animal health, promote gut health for efficient nutrient utilization, animal welfare, and overall performance without the adverse effects associated with antibiotics.

In this context, phytobiotics have emerged as a promising alternative to antibiotics, offering significant potentials as gut enhancers. Phytobiotics which include essential oils (EOs), plant extracts, herbs and spices, are plant-derived products that have gained recognition as a superior alternative to antibiotics; these natural products provide benefits without the risks of antibiotic resistance and residues, that can compromise both consumer safety and animal welfare [12]. Phytobiotics have demonstrated the ability to enhance gut health and overall growth performance, presenting a safer and more sustainable option for broiler production. Our previous review findings revealed that gut health is crucial for maintaining overall health and performance in broilers, encompassing enhanced intestinal antioxidant capacity, immune function, epithelial barrier integrity, gut microbiota composition, and villi morphology [13]. The antioxidant, antibacterial, and anti-inflammatory properties of these natural products; aromatic plants, herbal extracts, and EOs support gut health and growth performance, reinforcing their potential as effective alternatives to antibiotics [14, 15]. Our earlier findings revealed the efficacy of phytobiotics as natural antimicrobials and antioxidants to extend egg shelf-life by maintaining oxidative stability of the albumen [16]. Likewise, a comprehensive examination of natural plant-based additives revealed their positive impact on oviduct health, physiological response, and health status, boosting laying performance [17]. This shift supports sustainable broiler farming by leveraging the antioxidant, antibacterial, and anti-inflammatory properties of these natural products: reducing the reliance on antibiotics, mitigating the risk of antibiotics resistance and potentially leading to healthier, more resilient flocks, thereby contributing to safer animal products (meat).

Evidence increasingly supports the efficacy of natural plant-based additives over synthetic products as potential gut enhancers in broiler production. For instance, grape seed and oregano essential oil (OEO) have been shown to enhance growth performance and gut health more effectively than synthetic antioxidants [18, 19]. Recent research findings highlight the superior effects of natural products including EOs [20, 21] and plant extracts [22, 23], in improving growth performance, serum antioxidant capacity, immune function, and the balance of gut microbiota compared to antibiotics. The potency of phytobiotics may be further enhanced through synergistic combinations and optimal dosage levels. Studies have shown that the synergistic effects of certain combinations, such as polysaccharides (*Enteromorpha prolifera*) and yeast glycoprotein [24], organic zinc plus pectin oligosaccharides [25], or xylooligosaccharides plus gamma-irradiated *Astragalus* polysaccharides [26], can significantly enhance gut health compared to single components. Also, a more substantial antimicrobial effect was significant for the essential oil blend compared to the single impact of each component [27].

Conversely, not all phytogenic blends yield positive results. For example, a blend of hops, liquorice, and gum arabic had no significant effect on growth performance across all growth phases, possibly due to dosage limitations of individual components [28]. Moreover, while tannins, often considered antinutritional factors, were found to enhance villi morphology and growth performance [29]. The study demonstrated that a lower dosage (100 mg/kg) and not beyond this dosage provided an optimal improved antioxidant capacity, gut health, and nutrient absorption, which collectively contribute to enhanced growth performance and villi morphology. Eucalyptus globulus oil, an EO can increase the risk of skin irritation and toxicity at higher dosages [30]. Lavender essential oil (LEO) was found to improve mucosal immunity/inflammatory response at an inclusion level of less than 600 mg/kg, and higher levels did not enhance inflammatory response [31]. Recent advancements in feed technology such as microencapsulation techniques have been developed to protect the bioactive compounds in natural products from oxidative and mechanical degradation in the gastrointestinal tract, ensuring targeted release and improving bioavailability [32]. Research findings on these advancements demonstrated that microencapsulated forms of EO [33] and turmeric [34], exhibit more consistent and potent effects on gut health and performance than their free forms. However, determining the right combination and dosage to achieve optimal gut health benefits without causing negative interactions between components remains a challenge. These findings suggest that while phytobiotics offer promising alternatives to antibiotics, challenges remain in optimizing their efficacy and ensuring consistent performance outcomes, particularly concerning the combination form, dosage, and nature of the phytobiotic product.

This review synthesizes recent research findings, offering compelling evidence on the positive impact of phytobiotics on gut health, as demonstrated in studies conducted over the past five years on broilers reared under standard experimental conditions without stressors. It explores the mechanisms of action of phytobiotics, their benefits in broiler nutrition, and the challenges and limitations associated with their use. The review also provides insights into future directions for phytobiotic research, practical applications in the poultry industry, and strongly supports the industry’s shift towards more natural, sustainable, green feed additives.

## Methodology

This review systematically gathered, analyzed, and synthesized research on the impact of phytobiotics in broiler chicken nutrition. A literature search was conducted using databases such as PubMed, Web of Science, Scopus, and Google Scholar, focusing on studies from the past five years. Keywords included “phytobiotics”, “broiler chickens”, “gut health”, “antioxidant properties”, “immune modulation”, “gut morphology”, “gut microbiota”, and “growth performance”. Inclusion criteria were limited to peer-reviewed articles in English that examined phytobiotics, such as EOs, plant extracts, herbs, and spices, in broiler diets. Studies had to report on gut health, antioxidant capacity, immune response, or growth performance and include control groups or comparisons with synthetic antibiotics. Excluded were non-peer-reviewed publications, studies on species other than broilers, studies on broilers that introduced stressors such as disease challenge, heat stress, mycotoxins or any related forms, and articles focused on human health or non-poultry animals. Out of 200 articles that covered various aspects of broiler nutrition, 100 studies attained the inclusion and exclusion criteria for the key studies while other studies were used to substantiate claims. Data from selected studies were extracted and categorized qualitatively into themes like antioxidant function, gut morphology, gut microbiota composition, inflammatory and immune response, and growth performance. Each study was critically assessed for design, sample size, and methodological rigor. The synthesized data were then presented to highlight key findings on the role of phytobiotics in broiler nutrition as gut enhancers and influence on growth performance. Providing a concise overview of current trends, challenges, and future research directions.

## Critical relevance of gut health to broiler performance and overall health

Gut health encompasses intestinal antioxidant function, immune response, morphology barrier function, and gut microbiota modulation, which all work in synergy to maintain gut homeostasis for enhanced animal performance and overall health.

### Antioxidant and immune function

The gut immune function and antioxidant activity are deeply intertwined, forming a complex network that is vital for maintaining gut health and overall physiological homeostasis. ROS, such as superoxide, hydrogen peroxide, and hydroxyl radicals, along with reactive nitrogen species (RNS) like nitric oxide, are by-products of normal cellular metabolism, including in gastric epithelial cells. While these molecules are involved in essential cellular processes, their overproduction can overwhelm the body’s natural antioxidant defenses, leading to oxidative stress [8].

In the gut, oxidative stress disrupts the delicate balance of cellular environments by compromising the integrity of tight junctions in the gut epithelium, which reduces trans-epithelial electrical resistance (TEER) and alters ion transport [35]. The damage to tight junctions increases paracellular permeability, allowing harmful substances to pass through the gut lining. This process is exacerbated by ROS, which not only degrades tight junction proteins by activating matrix metalloproteinases (MMPs) but also triggers inflammatory signaling pathways, such as nuclear factor kappa B (NF-κB). Moreover, ROS interfere with ion channels, elevate intracellular calcium levels, and induce endoplasmic reticulum stress, all of which impair protein folding and calcium homeostasis, further destabilizing gut function [36]. These disruptions underline the critical role of antioxidants in preserving gut integrity and maintaining efficient ion transport across the gut barrier.

In broilers, oxidative stress has profound implications, not only impairing cellular membranes but also disrupting key metabolic processes, ultimately leading to reduced productivity [37]. Broilers, bred primarily for rapid muscle growth, are particularly vulnerable, as oxidative stress damages key cellular components such as lipids, proteins, and DNA, which hinders muscle development, accelerates lipid oxidation in muscle tissues, and diminishes meat quality [38], invariably resulting to poor economic returns. Additionally, oxidative stress stimulates enzymes such as nitric oxide synthase and peroxidase oxygenase, which increase the production of proinflammatory cytokines, further compromising gut health [39]. This heightened inflammatory response exacerbates the negative impacts on broiler productivity by impairing gut function and overall physiological health, underscoring the importance of antioxidant interventions in poultry nutrition.

The connection between oxidative stress and gut immune function is intricately linked, as oxidative damage not only disrupts the physical barriers of the gut but also triggers inflammatory responses that further compromise gut health. The overproduction of ROS upregulates genes associated with both innate and adaptive immune responses, including key inflammatory mediators like tumor necrosis factor (*TNF-α*), interleukins (*IL-6* and *IL-1β*), play central roles in the body’s response to oxidative stress [40, 41]. For instance, *TNF-α* activates signaling pathways like NF-κB and mitogen-activated protein kinases (MAPKs), leading to the production of additional inflammatory cytokines perpetuating the cycle of inflammation. Similarly, *IL-6* promotes the differentiation of T cells, particularly Th17 cells, which produce IL-17, a cytokine associated with chronic inflammation and mucosal damage. *IL-1β*, which facilitates the recruitment of immune cells to inflamed mucosa, can also be modulated by antioxidants, resulting in reduced immune cell recruitment and decreased mucosal inflammation. Antioxidants counteract these effects by neutralizing ROS, suppressing the activation of these pathways and decreasing the oxidative signals that drive the production of these cytokines; all of which will reduce overall inflammation, and preserve the integrity of the gut barrier [42–44].

The gut’s susceptibility to oxidative stress is further compounded by its high oxidative metabolism, which constantly exposes the intestinal epithelium to oxidative stimuli. Maintaining redox equilibrium is therefore essential for preserving intestinal integrity and overall homeostasis. Endogenous antioxidant enzymes, such as superoxide dismutase (SOD), catalase (CAT), and glutathione peroxidase (GSH-Px), are vital in defending against ROS. However, when these defense systems are overwhelmed by external stressors, oxidative damage ensues, compromising cellular membranes, DNA integrity, and other vital biological molecules [45]. This underscores the need for exogenous antioxidants that can bolster these cellular defenses and activate related signaling pathways. Highlights the significant relevance of gut immune function which depends on a coordinated network of immune organs, immunoglobulins, and cytokines to maintain effective immune defense while ensuring tolerance to harmless antigens. This function is indispensable for immune defense in the gut while maintaining tolerance to dietary antigens and commensal microbiota. Peyer’s patches house B cells, T cells, and dendritic cells that sample gut antigens, thus can trigger appropriate immune responses, mesenteric lymph nodes filter lymph and regulate differentiation of immune cells, lamina propria, populated with immune cells such as macrophages, T cells, and plasma cells, produce immunoglobulins crucial for mucosal immunity. Also, the thymus and Bursa of Fabricius ensure the proper maturation of T-cells and B-cells, after maturation these cells populate gut lymphoid tissues and work in synergy to maintain overall immune homeostasis [46, 47].

Immunoglobulins (IgA, IgG and IgM) are vital to gut immune function; IgA is the primary antibody in the gut, protecting the intestinal barrier by neutralizing pathogens and toxins. While IgG and IgM are less abundant in the gut compared to IgA, they remain vital for neutralizing pathogens that breach the mucosal barrier and enter the systemic circulation. IgM, in particular, acts as a first-line defense, initiating the immune response before sufficient levels of IgA are produced [48]. Secretory immunoglobulin A (sIgA), produced by plasma cells in the intestinal lamina propria, is most abundant in the small intestine and is critical for maintaining the gut immunity and tolerance, enhancing intestinal integrity and function. Elevated levels of gut immunity markers like sIgA may enhance intestinal integrity, contributing to better gut function [49].

In the context of gut immunity, cytokine production and the release of inflammatory mediators are central to the immune response but can damage tissue and impair the function of intestinal epithelial cells when unregulated. Oxidative stress exacerbates this by promoting intestinal inflammation and cell death within the intestine, ultimately leading to intestinal barrier dysfunction [38]. T helper (Th) cells, particularly Th1 and Th17 cells, are crucial in regulating these immune responses. Th1 cells, produce cytokines such as Interferon-gamma (IFN-γ), IL-2, IL-6, and TNF-α, which are vital to immune system development (innate and adaptive immunity). Th17 cells, produce IL-17 and IL-22, involved in defending the gut against extracellular pathogens like bacteria and fungi. IL-22 promotes the production of antimicrobial peptides and strengthens the epithelial barrier, offering additional protection against pathogens. IL-6 promotes cellular immunity and infection. However, an overexpression of Th1 cells or overabundance of IL-17 can trigger apoptosis, disrupt intestinal integrity and contribute to inflammatory diseases. Th2 cells produce anti-inflammatory cytokines like IL-10 and IL-4, which are essential for maintaining gut homeostasis by balancing pro-inflammatory and anti-inflammatory signals [47, 50]. A balanced expression of IFN-γ and IL-4 is indicative of immunological equilibrium [51]. The balance between these cytokines along with the interplay between Th17 and Tregs cells, inhibition of the NF-κB pathway is essential for maintaining immune homeostasis and preventing chronic inflammation, emphasizing the importance of antioxidants in modulating these responses.

Given the challenges posed by oxidative stress, there is a clear need for exogenous antioxidants that can bolster cellular defenses and support the gut’s immune system. Nutritional interventions, especially phytobiotics, natural plant-derived compounds with antioxidant, anti-inflammatory, antimicrobial, and immunomodulatory properties offer a promising approach. These compounds enhance the activity of cellular antioxidant enzymes and modulate immune responses, promoting overall health and productivity in poultry. Phytobiotics have shown great potential in modulating gut immunity and maintaining this delicate balance by influencing the production of chemokines, cytokines, and mucosal IgA through Toll-like receptors (TLRs), which alter the expression of pro-inflammatory and anti-inflammatory cytokines in the intestinal mucosa. Additionally, phytobiotics can modulate the gut microbiota to produce butyrate, a short-chain fatty acid that prevents inflammation by regulating gene expression related to pro-inflammatory cytokines and T-cell differentiation [52, 53]. By stimulating intracellular signaling pathways and modifying the transcriptional expression of inflammatory mediators, phytobiotics protect the intestinal epithelium from inflammation and reinforce the gut’s immune defenses.

In conclusion, the interaction between antioxidant function and gut immune function is critical for maintaining overall gut health and animal productivity. Antioxidants, particularly those derived from phytobiotics, play a pivotal role in reducing oxidative stress and inflammation, thereby preserving the integrity of the gut barrier and supporting the immune system. These interventions not only enhance the health and productivity of poultry but also contribute to a more sustainable and effective approach to animal nutrition. The synergy between antioxidant defense and immune regulation underscores the importance of integrated nutritional strategies in promoting optimal gut health and overall well-being in broilers.

### Intestinal barrier function

The intestinal mucosal barrier is a complex system composed of intestinal mucosal epithelial cells that are intricately interconnected through tight junctions (TJ), adhesion junctions (AJ), and desmosomes [54]. These structures are pivotal for maintaining the integrity of the epithelium and the mucus gel layer, thereby, playing a crucial role in preventing pathogen invasion and facilitating the paracellular transport of water, chemicals, and ions, which are key components of intestinal homeostasis and barrier function [55]. Proteins such as zona occludens-1 (ZO-1), occludin (OCLN), and claudins (CLDN) are integral to the structure of tight junctions, while E-cadherin is a significant component of the adhesion junctions. Increased mRNA expression of these tight junction proteins indicates robust intestinal integrity and barrier function, with ZO-1, for instance, being crucial in regulating intestinal barrier and permeability, thereby enhancing the resistance of the intestinal epithelium against pathogenic invasion [54].

In addition to the structural proteins, goblet cells contribute to this defense system by secreting mucins, which coat the surface of the intestinal mucosa and maintain its thickness, thereby preventing pathogen adhesion. The upregulation of *MUC-2*, not only promotes proliferation of native microbiota and mucus production, but also protects the epithelial cells from endotoxins [56]. This protective mechanism allows resident beneficial microbes to serve as the first line of defense against pathogen-induced intestinal damage. Moreover, antimicrobial peptides such as mucins and avian β-defensins, along with sIgA, play a crucial role in regulating intraepithelial lymphocytes (IELs) and preserving intestinal integrity [57, 58]. Defensins, known for their ability to directly target and neutralize pathogenic microorganisms, work synergistically with mucins—heavily glycosylated proteins, that form a protective mucus layer that serves as a scaffold for sIgA. The presence of sIgA in the mucus layer enhances the barrier’s protective functions and modulates the immune response by interacting with IELs, thereby ensuring the maintenance of intestinal homeostasis. Avian β-defensins, small cationic peptides, contribute to the innate immune response by disrupting the membranes of pathogens and modulating the function and population of IELs. Collectively, the actions of mucins, sIgA, and avian β-defensins form a robust defense system that not only protects the gut from infections but also regulates immune cells within the intestinal epithelium, ensuring the maintenance of a healthy gut barrier.

The functionality of the intestinal barrier is also influenced by metabolic products like D-lactic acid (D-LA) and enzymes such as diamine oxidase (DAO). The D-LA is produced by the fermentation of intestinal bacteria, while DAO is released by the intestinal epithelial cells in the upper villi of the intestinal mucosa and ciliated cells [59]. The key function of DAO is to break down histamine, a compound involved in various physiological processes including immune responses. Excessive histamine can trigger inflammation, increase intestinal permeability by loosening the tight junctions between epithelial cells, and potentially lead to tissue damage [60]. Hence, DAO plays a crucial role in regulating the intestinal barrier by metabolizing histamine, controlling immune responses, maintaining gut integrity, and preventing inflammation-related intestinal disorders [61]. Whereas, D-LA is incorporated into the phospholipids of cell membranes, including those of the intestinal epithelial cells, helping to maintain their structural integrity, which is vital for preventing passage of harmful substances into the bloodstream [62]. Additionally, D-LA can be converted into anti-inflammatory eicosanoids, signaling molecules that help modulate inflammation, thereby supporting the intestinal barrier integrity [63]. Both DAO and D-LA are valuable biomarkers for assessing intestinal barrier function; their presence in the blood circulation indicates a compromise in barrier integrity [64].

Oxidative stress-induced inflammatory stress response can severely damage the intestinal epithelium, which is a single layer at the luminal interface of the organism. The integrity of this epithelium is maintained by tight junctions, and destabilization of these proteins can increase intestinal permeability, leading to the influx of pathogens and toxins that impair mitochondrial function and disrupt the intestinal epithelium [65]. The ROS generated during oxidative stress can downregulate tight junction proteins such as ZO-1, claudin-1, -2, occludin, and mucins [66, 67]. This downregulation is often triggered by the activation of NF-κB pathway which can repress the transcription of genes encoding TJ proteins by recruiting co-repressors or by competing with other transcription factors that promote TJ protein expression [68]. *NF-κB* activation also induces the expression of MMPs, which degrade extracellular matrix components and tight junction proteins, while increasing levels of *TNF-α* that can lead to the internalization and degradation of occludin and ZO-1 from the tight junction complex [69]. The resulting downregulation of tight junction proteins compromises the intestinal barrier function. For instance, a knockdown of occludin distorts structural integrity and increases paracellular macromolecule permeability, facilitating enteric infections and endotoxin translocation [70]. Disruptions in intestinal integrity heighten intestinal permeability, pathogen adherence to the mucosal epithelium, and intrusion of luminal antigenic elements, leading to intestinal lesion sores, inflammation, and immune responses that adversely affecting the entire gut and metabolic processes.

It could be inferred that intestinal barrier cues, including DAO, mucins, goblet cells, and tight junction proteins, play critical roles in regulating the intestinal epithelial barrier against infections and endotoxins. Phytobiotics could play a crucial role in regulating these cues to bolster intestinal epithelial integrity, which supports villi development for nutrient metabolism.

### Intestinal morphology

Healthy intestinal epithelial barrier and intestinal shape are crucial for animal health, enhancing immunity, pathogen protection, and promoting growth [71]. Critical indicators for efficient gut morphology include longer villi, a higher villus height (VH) to crypt depth (CD) (VH/CD) ratio, and shorter crypts, which suggest well-developed enterocytes. An increase in the VH/CD ratio is associated with higher goblet cells, which produce mucin essential for maintaining intestinal barrier function. Increased VH is simultaneous with higher intestinal surface area and number of epithelial cells, which enhance nutrient absorption [72]. Intact villi allow gut modulation by microbiota to protect against enterocyte damage and promote villus regeneration and maturity, which release more nutrient metabolism-stimulating enzymes [73]. Hence, this efficient intestinal architecture increases mucosal absorptive surface area, intestinal cell proliferation, epithelial development, brush border enzymes, nutrient transport systems, and nutrient bioavailability, which are vital for improved growth performance [74].

Improved nutrient metabolism correlates with increased enzyme secretion and upregulation of nutrient transporters modulated by efficient villi development. Enhanced expression of genes linked with nutrient transport such as sodium-glucose cotransporter 1 (*SGLT1*), glucose transporter 2 (*GLUT2*), other solute carriers (SLC) family transporters (*SLC38A* and *SLC79A*), and fatty acid transport protein 4 (*SLC27A4*), is indicative of superior villi architecture, which is crucial for gut health and host performance [11]. The enhanced expression of nutrient transport genes like *SGLT1*, *GLUT2*, and various SLC transporters (*SLC38A*, *SLC79A*, and *SLC27A4*) directly correlates with superior villi architecture in the small intestine. This increased gene expression leads to the production of more transport proteins, which improves the absorption of nutrients such as glucose, amino acids, and fatty acids. As nutrient absorption efficiency rises, the villi adapt by becoming taller and more densely packed, increasing the surface area available for absorption. This structural enhancement of the villi is critical for optimizing gut health and overall performance, creating a cycle where better gene expression supports improved villi structure, leading to enhanced nutrient uptake.

However, oxidative stress accruing from digestive oxygen radicals damages intestinal mucus and villi; this damage manifests as shorter villi, shallower crypts, delayed gut epithelial cell renewal, and compensatory crypt hyperplasia, which impairs nutrient absorption and indirectly affects microbiota structure vital for gut epithelium formation [75]. Phytobiotics offer a promising strategy for scavenging or inhibiting oxygen radicals on the mucosal surface, thereby improving villi shape for nutrient utilization and modulation of the gut by beneficial microbes.

### Intestinal microbiota

The intestinal microbiota plays a pivotal role in modulating host performance and health through several intricate mechanisms, including nutrient metabolism, immune modulation, and antimicrobial functions. The balance and composition of gut bacteria, both pathogenic and symbiotic, are primarily determined by competition for resources and attachment sites within the gut. Among the key intestinal phyla in avian microbiota, Firmicutes, Bacteroidetes, Proteobacteria, Tenericutes, and Actinobacteria, are the key contributors to host metabolism and the regulation of various physiological processes. Regular residents of the chicken’s intestinal tract, such as *Escherichia coli*, *Enterococcus*, *Clostridium*, and *Lactobacillus*, further underscore the complexity of this microbial ecosystem. Firmicutes, known for their role in the fermentation of dietary fibers, produce short-chain fatty acids (SCFAs) like butyrate, propionate, and acetate, which are crucial for gut health. The dominance of this phylum is linked with the suppression of pathogenic bacteria, thereby protecting the host from infections [76]. On the other hand, Bacteroidetes are essential for the breakdown of complex carbohydrates, converting them into simpler molecules that the host can readily absorb, making them integral to carbohydrate metabolism [77]. The balance between Firmicutes and Bacteroidetes is often reflective of the host’s metabolic state, where a favorable Firmicutes-to-Bacteroidetes ratio is typically associated with enhanced physiological function. Proteobacteria, although less dominant, comprise a diverse group of bacteria with roles that range from beneficial functions like nitrogen fixation and nutrient synthesis to opportunistic pathogenicity. These bacteria become more prominent during periods of dietary change or stress, impacting overall gut health and functionality [78]. Tenericutes, though less explored, contribute to the synthesis of essential metabolites and modulation of the host’s immune response. Their prevalence in birds with varied diets suggests their adaptive role in response to dietary diversity [79]. Actinobacteria, though less abundant, are vital for vitamin production, particularly B vitamins, and the breakdown of plant polysaccharides. This phylum also aids in suppressing pathogenic microbes through the production of antimicrobial compounds, which enhance gut health and bolster immune function [80]. The diverse roles of these phyla accentuate the complexity and importance of gut microbiota in maintaining the overall health and metabolic balance of the host.

The complexity and significance of gut microbiota in maintaining host health and metabolic balance are further underscored by the specific roles of these microbes. *Bacteroides* and *Ruminococcus* enriched in cecum, are essential for microbial fermentation [81]. In the cecum, the Firmicutes and Bacteroidetes phyla play critical roles in nutrient absorption and metabolism [77], with *Clostridia* species contributing to starch digestion, protein breakdown, and butyrate production [76]; *Bacteroidetes* ferment polysaccharides and other indigestible carbohydrates into acetate and propionate and encode polysaccharides that aid sugar metabolism [82]. *Romboutsia* ferment carbohydrates to SCFAs, oligosaccharides, and other prebiotics associated with metabolizing blood triglycerides, total cholesterol, and weight gain [83]. *Alistipes* and unclassified Rikenellaceae belong to phylum Bacteriodales. *Alistipes* can produce succinic acid and long-chain fatty acids like C15, which aids intestinal lipid metabolism, while Rikenellaceae ferment mannose, galactose, and glucose, benefiting energy metabolism [81].

Besides, nutrient metabolism, these bacteria are primarily involved in production of SCFAs which have multifaceted benefits on gut health. *Blautia* degrade complex polysaccharides and produce branched-chain fatty acids such as isobutyric acid, 2-methylbutyric acid, and 3-methylbutyric acid that enhance gut morphology [84]. Also, *Faecalibacterium* and *Phascolarctobacterium* are critical producers of butyric and acetic acid, which have multiple gut benefits [85]. *Lactobacillus* uses phenols as substrates and produces vitamins and organic acid (OA), facilitating nutrient absorption and intestinal function [86]. The microbiota’s capacity to degrade carbohydrates and fibers into SCFAs is central to gut health, as these acids provide energy to epithelial cells, regulate glucose metabolism, and promote the growth and diversity of beneficial microbes. The production of butyrate, in particular, is associated with the expression of tight junction proteins like ZO-1, which are vital for maintaining gut integrity [87].

In addition to their metabolic roles, gut microbes significantly influence host immunity. Specific microbes like *Faecalibacterium prausnitzii* have been linked to anti-inflammatory effects, partly due to their butyrate production and enhanced secretion of IL-10, which inhibits pathogen invasion and sustains lysozyme activity [88, 89]. *Prevotella* ferments indigestible carbohydrates to butyrate, reducing intestinal inflammation and permeability [90], while, *Parabacteroides* promotes gut integrity by exerting anti-inflammatory effects and enhancing tight junction protein expression [91, 92]. Microbes such as *Succiniclasticum* and *Akkermansia* are involved in immunoglobulin secretion, which supports immune function [93]. Nevertheless, pathogens such as *Desulfovibrio* generate toxins, inhibit SCFA oxidation, especially butyrate, and disrupt intestinal epithelial hydrogen sulfide detoxification processes [68]. *Bacteroides* and *Desulfovibrio* positively correlated with increased levels of inflammatory cytokines (IL-1β, IL-6, and TNF-α) [55], contributing to gut inflammation and compromised gut integrity.

Understanding the regulatory effects of gut microbiota on nutrient metabolism, gut integrity, villi development, immune response, and overall microbiota composition is crucial for advancing host health. The evidence underscores the profound significance of gut health to host metabolism, with various research findings highlighting the potential of phytobiotics as natural gut enhancers. These compounds offer promising prospects as antioxidants, boosters of intestinal integrity and barrier function, energy sources for gut architecture, immunological regulators, and microbial diversity enriches. Thus, maintaining a balanced gut microbiome is essential for optimizing host health, which necessitates ongoing research into effective interventions that can enhance gut microbiota composition and function.

## An overview of phytobiotics

Phytobiotics, derived from plant sources such as herbs, spices, EOs, and extracts, have garnered significant attention in animal nutrition, particularly in poultry diets, due to their potential to enhance health and performance. As natural alternatives to synthetic antibiotics, phytobiotics offer a range of benefits including antimicrobial, antioxidant, immune-modulating, and growth-promoting effects. Their use is linked to improvements in gut health, where they enhance gut morphology, support nutrient absorption, and maintain a balanced population of beneficial bacteria while suppressing harmful pathogens [94, 95]. The bioactive compounds found in phytobiotics, including saponins, flavonoids, terpenoids, phenolic compounds, alkaloids, polysaccharides, EOs, and phytosterols, are key to these beneficial effects of phytobiotics and are presented in Table 1 [96–113]. 

**Table 1:** List of bioactive compounds from different phytobiotics samples

Plant sample	Bioactive compound	Reference
*Glycyrrhiza glabra* (Licorice) extract	Glycyrrhizin, glycyrrhizinic acid, glabridin, glabrene, and glabrol	[96]
Chamomile flower extract	β-Famesene	[97]
Thymol oil extract	Gamma-terpinene and thymol	[98]
Olive leaf extract	Oleuropein, polyphenols and flavonoids	[99]
Fennel EO	Anethol, fenchon, *trans*-anethole	[100]
*Quillaja saponaria* and *Yucca schidigera*	Saponins polyphenols	[101]
*Froriepia subpinnata*	Thymol, carvacrol, lomonene and terpinene	[102]
*Pulicaria jaubertii*	Dimethoxy dimethylsilane, benzaldehyde thiosemicarbazone	[103]
*Pulicaria gnaphalodes*	1,8-Cineole	[104]
Turmeric	Curcumin and turmerones	[34]
*Coriandrum sativum*	Carvone, geraniol, limonene, camphor and linalool	[105]
*Epimedium*	Prenyl flavonols: Icarin, epimedin A-C and baohuoside 1	[106]
*Cynara cardunculus* (Artichoke)	Cynarine	[107]
*Pueraria* extract	Puerarin	[108]
*Portulaca oleracea* L.	Phenolic alkaloids such as oleraceins	[109]
Radix *Sophorae flavescentis*	Alkaloids and flavonoids	[110]
*Thalictrum glandulosissimum*	Berberine	[111]
*Terra flavausta*	Silicic acid	[112]
*Pogostemon cablin*	Phytosterols, flavonoids	[113]

*EO* Essential oil

The antioxidant properties of phytobiotics are particularly noteworthy, as they play a critical role in reducing oxidative stress and maintaining intestinal homeostasis. For instance, grape skins and seeds contain phenolic compounds such as catechins, anthocyanins and epicatechin, which are effective at scavenging free radicals, thus protecting cellular membranes from oxidative damage [114]. Resveratrol, a potent antioxidant found in nuts, grape skins, and berries, enhances anti-inflammatory pathways by inhibiting the activation of the NF-κB pathway, thereby preventing the degradation of IκB, the inhibitor of NF-κB. This mechanism helps keep NF-κB sequestered in the cytoplasm, reducing inflammatory responses. Also, scavenges ROS which are key mediators of inflammatory pathways including NF-κB/MAPKs [115, 116]. Additionally, magnolol, a polyphenolic compound neolignan found in *Magnolia officinalis* bark has been shown to mitigate mitochondrial dysfunction via scavenging of ROS [117], promote phagocytosis [118], and upregulate *Nrf2* expression in hepatocytes [119], which in turn reduces cellular inflammation and supports gut health.

Phytobiotics also influence enzyme secretion, gut integrity, and villi renewal, all of which are essential for efficient nutrient uptake and utilization. Active compounds such as sanguinarine and chelerythrine are known to promote villi growth, increasing the absorption area in the intestine [120]. EOs like eucalyptus have been shown to enhance transepithelial electrical resistance and monocyte phagocytic activity, while cineole modulates epithelial mucus secretion, benefiting both intestinal cell wall integrity and the immune system [121].

Furthermore, saponin-containing EOs enhance the activity of digestive enzymes particularly proteases, facilitating the efficient digestion of dietary proteins and essential elements, which are vital for intestinal development [122]. Compounds such as capsicum oleoresin, cinnamaldehyde, and carvacrol further contribute to gut health by enhancing mucin synthesis, which inhibits pathogen adhesion to mucosal surfaces, thereby promoting gut integrity [123].

The antibacterial properties of phytobiotics are extensive and multifaceted. For example, curcumin and its bisdemethoxy and dimethoxy derivatives [124], eugenol in cloves powder and tulsi extract [125], and gallic acid in Roselle [126], have been shown to effectively suppress pathogens. The bioactive compounds in elecampane rhizome, such as alantolactone and isoalantolactone, disrupt bacterial cell membrane integrity, energy transduction, and other critical cellular processes [127, 128]. Similarly, a phytogenic blend (wheat germ, hops, and grape seed extract), exhibits synergistic antibacterial effects through its constituents like xanthohumol, flavonoids, lectins, tannins, and β-acids [129]. Also, an in vitro experiment demonstrated that antimicrobial peptides in sesame meal bioactive peptide (SMBP) inhibit tetrahydrofolate synthesis, key components of cell replication, further demonstrating the broad-spectrum antibacterial effects of phytobiotics [130]. Likewise, other bioactive compounds of therapeutic and aromatic herbs structurally modify entero-pathogen cellular membranes, inducing ion leakage and pathogen damage [131]. They also bind to epithelial sites and produce antibacterial bacteriocins, which increase gut commensal proliferation, protective effect against pathogens, and immune status [132].

The primary elements of plant essential oils include carvacrol (28.20%), menthol (16.62%), thymol (9.40%), ρ-cymene (9.25%), 1,8‐cineol (5.70%), menthone (4.84%), γ‐terpinene (4.49%) and α‐pinene (3.10%) [133]. The lipophilic properties of EOs allow them to permeate bacterial cell walls and membranes, disrupting the bacterial structure and preventing pathogen invasion [134]. For example, carvacrol alters ion channel permeability, while cinnamaldehyde disintegrates bacterial membranes, reducing ATP synthesis and degrading enzymatic systems [135, 136]. Plant EOs exhibit a broad-spectrum antibacterial effect, promote mucin synthesis in the intestinal tract, and inhibit mycotoxin generation, cell communication, and fungal biofilm, enabling epithelial cell proliferation for intestinal villus development [137, 138]. This substantiates the earlier report that the strong antimicrobial effects of plant EOs are linked to their broad-spectrum nature [139].

These broad-spectrum antibacterial effects, along with the ability of phytobiotics to promote mucin synthesis, inhibit mycotoxin generation, and enable epithelial cell proliferation, underscore their potential as effective natural gut enhancers. By upregulating antioxidant enzymes and genes [140], mucins, tight junction proteins [141], and supporting villi renewal [142], phytobiotics would protect the integrity of biological molecules (DNA, enzymes, proteins, carbohydrates, and lipids), and prevent gut pathogen invasion due to the stimulatory effect of bioactive compounds on mucin production. This could be a positive direction for improving growth performance in broiler production. For instance, in a study involving 3,000 broilers, supplementation with a herbal mixture consisting of (stems and leaves of spearmint, thyme, and yucca; seeds of pepper and black cumin; roots of ginger; and developing bulb part of onion and garlic), demonstrated significant benefits in growth performance, antioxidative function, and humoral immunity compared to antibiotics [66]. The organic nature and bioactive constituents of phytobiotics contribute to their effectiveness against antibiotic resistance, making them safe and acceptable feed additives for poultry health and performance.

However, the use of phytobiotics is not without challenges. Factors such as extraction methods, dosage, and application techniques can limit their effectiveness. Continued research into the mechanisms of action of phytobiotics will be essential in facilitating their integration into poultry nutrition strategies, ensuring they reach their full potential as natural, sustainable alternatives to conventional antibiotics.

**Fig. 1:**
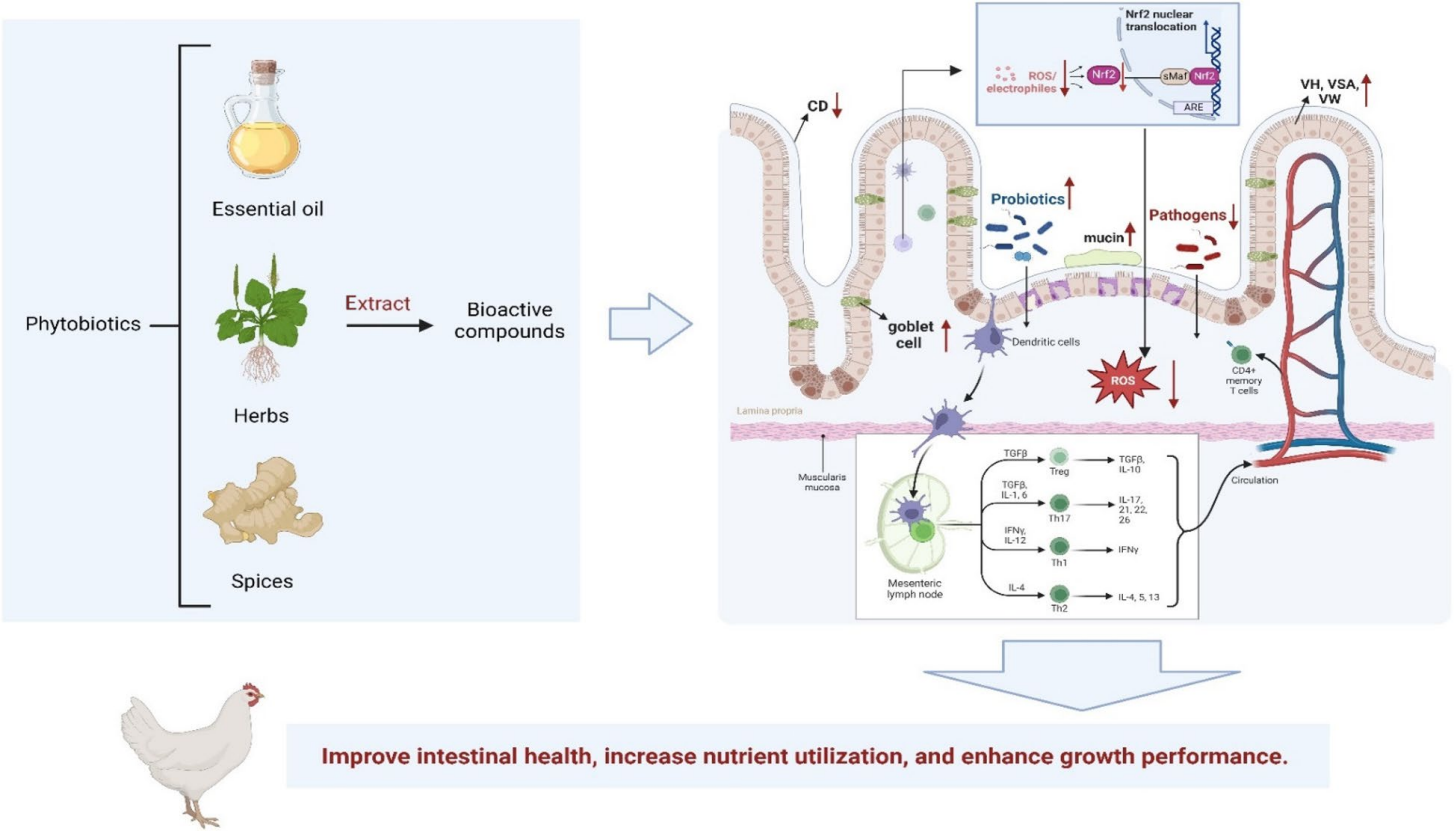
Overall impact of various bioactive components of phytobiotics on gut health of chicken. Created with BioRender.com

Various phytobiotics with specificity to dosage level, overall effect and implication for broiler performance are presented in Additional file 1. As presented in Fig. 1, phytobiotics have the potential to scavenge free radicals, protect the integrity of biological molecules (DNA, enzymes, proteins, carbohydrates, and lipids), and prevent gut pathogen invasion due to the stimulatory effect of bioactive compounds on mucin production and the hydrophobicity effect. Additionally, it upregulates tight junction proteins, promotes mucin secretion, and reduces intestinal permeability biomarkers.

## Mechanism of action of phytobiotics on gut health

The underlying mechanism of action of phytobiotics on broiler birds’ health, welfare, and performance may be explained by its effect on intestinal antioxidant function, immune and inflammatory response, intestinal barrier integrity and function, villi morphology, and antimicrobial effects. The section provides specific information relating to each mechanism of action observed in broiler birds under standard experimental research models without stressors.

### Effects of phytobiotics on gut antioxidant function

Phytobiotics, recognized for their diverse bioactive components and minimal toxicity, have emerged as natural antioxidants capable of maintaining internal homeostasis in animals. The antioxidant function of phytobiotics in broilers has been well-documented, showcasing their ability to enhance antioxidant defenses across various tissues, including the gut. Some of the research studies are presented in Table 2 [143–154], while others are presented in the text.

**Table 2:** Effect of phytobiotics on the antioxidant function of broiler birds

Diets	Antioxidant genes	Antioxidant enzymes	MDA	References
EO (thymol, carvacrol and cinnamaldehyde) at 200 or 400 mg/kg	NE	SOD**, (GSH-Px, T-AOC)^ns^	NS	[11]
Oregano essential oil: natural or synthetic at 200 mg/kg each	NE	GR, GSH-Px, SOD, T-AOC**	R	[19]
Microencapsulated turmeric by maltodextrin: 1, 2, 3 g/kg diet	NE	TAC**	R	[34]
Curcumin (CUR) (200 mg/kg), *Pueria* extract (PE) (200 mg/kg) and CUR + PE (200 + 200 mg/kg)	NE	GSH-Px, CAT**, (T-SOD, T-AOC)^ns^	R	[108]
Beta sitosterol at 25, 50, 75, and 100 mg/kg	NE	GSH-Px, CAT**	R	[142]
Ferulic acid	NE	SOD, GSH-Px**	R	[143]
Licorice extract (*Glycyrrhiza glabra*) at 0.4–0.8 g/L of water	NE	GSH, CAT**	R	[144]
Encapsulated product (Capsicum blend with black pepper and ginger extract) at 250 ppm	*CAT*,* GPx1*,* SOD1*,* Nrf2*^*ns*^	CAT**, (GPx, GST, SOD)^ns^	R	[145]
100 mg/ kg *Forsythia suspense* extract (FESE)	NE	SOD, GSH-Px, CAT**	R	[146]
A blend (*Astragalus membranaceus* and *Codnopsis pilosula*) extract at 500 mg/kg	NE	T-AOC, TSOD, GSH-PX**, (CAT)^ns^	R	[147]
*Yucca* saponin (YSa), *Yucca schidigera* (YS), and *Quillaja* *saponaria* (QS) at 500 mg/kg	NE	T-AOC, GSH-Px**	R	[148]
Chinese herbal mixture at 500, 100 or 1,500 mg/kg	*Nrf2*,* HO-1*,* NQO1*,* SOD1*, GPX**	SOD, T-AOC**	R	[149]
*Ginseng*,* Astragalus*, *Salvia miltiorrhiza* at 1,000 mg/kg	NE	GSH-Px, CAT, T-SOD, T-AOC**	R	[150]
Cinnamon oil at 500, 1,000 and 1,500 mg/kg	NE	GSH-Px, GSH, CAT, SOD, TAC**	R	[151]
EO at 200, 400 and 600 mg/kg (Carrier: rice husk powder and silica)	NE	SOD, CAT, T-AOC**	I	[152]
Lavender EO at 300 or 600 mg/kg	NE	SOD, GSH-Px**	R	[153]

*NE* Not evaluated*,* *NS* Not significant, ** Significant, *R* Reduced, *I* Increased, *ADP* Algae-derived polysaccharides, *EO* Essential oil, *MDA* Malondialdehyde, *Nrf2* Nuclear factor erythroid 2-related factor2, *GPX1* Glutathione peroxidase 1, *SOD1* Superoxide dismutase 1, *SOD2* Superoxide dismutase 2, *HMOX1/HO-1* Hemoxygenase 1, *CAT* Catalase, *T-SOD* Total superoxide dismutase, *T-AOC* Total antioxidant capacity, *GSH-Px* Glutathione peroxidase, *GSH* Glutathione, *GR* Glutathione reductase, *NQO1*-*NAD*(P)H Dehydrogenase (quinone) 1

The profound antioxidant effect of phytobiotics may lie in its capacity to upregulate antioxidant genes and activate related pathways. For instance, it has been demonstrated that a polyherbal blend can significantly increase the mRNA expression of antioxidant genes and serum antioxidant enzyme levels while concurrently reducing serum malondialdehyde (MDA) [154]. These effects are attributed to the blend’s rich content of flavonoids, phenolic acids, and alkaloids, which collectively contribute to its potent antioxidant activity [155]. Similarly, the inclusion of *Dendrobium officinale* leaves in broiler diets has been shown to bolster the antioxidant capacities of both serum and intestinal mucosa, accompanied by a reduction in ROS levels [156]. The enhancement is primarily due to the high polysaccharide content in these leaves, which plays a crucial role in the antioxidant response [157]. Building on this, the synergistic effects of *Enteromorpha polysaccharides* and yeast glycoprotein extracts have been found to boost the mRNA expression of antioxidant-related genes and enzymes in broilers [24], an effect largely mediated by the upregulation of the Nrf2 signaling pathway [158]. This pathway is integral to the body’s antioxidant defense mechanisms, suggesting that phytobiotics can effectively activate molecular pathways that mitigate oxidative stress. Additionally, *Galla chinensis* tannin has demonstrated its capability to enhance antioxidant function by improving the expression of genes associated with the Nrf2/HO-1/SOD pathway, alongside significant increases in serum glutathione peroxidase (GSH-Px) levels [159]. The *Terminalia chebula* extract is another example, with its chebulic acid content driving the activation of the Nrf2 pathway and *HO-1* expression, further supporting the gut’s antioxidant capacity [160]. Evidence from research studies on oregano EO which contains bioactive compounds like carvacrol and thymol, suggested that the diet significantly upregulated the expression of antioxidants such as *GPX1*, *HMOX1*, and *Nrf2* in the ileal mucosa, as well as increased in serum GSH-Px levels [20]. This highlights the potential of phytobiotics to modulate key signaling pathways that underpin antioxidant responses in the gut and beyond; strengthening the gut’s antioxidant defenses.

Moreover, dietary phytobiotics can increase levels of antioxidant enzymes in the serum and intestinal mucosa. Marine algae-derived polysaccharides have been noted for their ability to enhance antioxidant enzyme activities [161], attributed to their hemiacetal hydroxyl structures, which contribute to ROS scavenging capabilities [162]. Ethanol extracts of elecampane (*Inula helenium* L.) rhizome [163] and anthocyanin-rich roselle (*Hibiscus sabdariffa *L.) extracts [164], also demonstrated dose-dependent antioxidant effects, likely due to the ROS scavenging capacity of alantolactone, isoalantolactone [165], and polyphenols [166], respectively. This indicates that the gut’s antioxidant response can be modulated by a variety of phytobiotic components, each contributing to a holistic enhancement of antioxidant functions. Furthermore, *Ilicis chinensis *folium extract, rich in phenolic and triterpenoid constituents [167], has been identified as another potent antioxidant [168], while LEO, containing monoterpenes such as linalool [169], has shown efficacy in reducing oxidative stress through its electron-donating properties [31]. In addition to these findings, β-sitosterol was found to enhance antioxidant function in the small intestine [142] by preserving cell membrane lipids from oxidative damage [170], demonstrating the diverse mechanisms through which phytobiotics exert their antioxidant effects. Although *Galla chinensis* extracts reduced MDA levels in the serum without significantly affecting antioxidant enzymes, this reduction in lipid peroxidation further highlights the multifaceted role of phytobiotics in mitigating oxidative damage [171].

Overall, the incorporation of plant-based natural antioxidants into broiler diets presents a compelling strategy to enhance gut health by providing antioxidant cues, inhibiting ROS generation, stimulating antioxidant enzyme secretion, and activating related signaling pathways. The ability of phytobiotics to activate these defenses and reduce oxidative stress underscores their potential to improve immune responses and overall animal health, making them valuable additions to animal nutrition regimens aimed at promoting longevity and productivity.

### Effects of phytobiotics on gut immune and inflammatory response

Plant-based feed additives have emerged as potent modulators of cytokine expression, playing a crucial role in balancing proinflammatory and anti-inflammatory factors to maintain equilibrium in the Th1/Th2 cytokine profile. This balance is essential for regulating immune responses in broilers, with research highlighting the positive effects of phytobiotics on both immune modulation and inflammation (Table 3). For instance, the anti-inflammatory properties of *Dendrobium officinale* leaves have been shown to be dose-dependent, contributing to increased weight of immune organs such as the thymus, spleen, and bursa of Fabricius, likely due to their polysaccharide content [157]. Similarly, essential oil blends have demonstrated potential immune benefits by improving the weight of the thymus and bursa of Fabricius [134]. A polyherbal mixture further supports immune health by increasing the organ index of the spleen and thymus and enhancing serum immunoglobulins and sIgA levels in the jejunal mucosa, reinforcing its role as an effective immunomodulator [154]. Nonetheless, ferulic acid enhanced the humoral immune response by increasing IgM levels, although their effects on IgG and IgA remained unaffected [143]. Also, a combination of cinnamon EO and bamboo leaf flavonoid showed no impact on the weight of immune organs [172], suggesting that not all phytobiotic combinations yield uniform benefits.

**Table 3:** Effects of phytobiotics on immune and inflammatory functions of broiler birds

Sample	Diets	Cytokines		Immunoglobulins		Effects	References
		Downregulated	Upregulated	IgA, IgM, IgG	sIgA		
I	Oregano essential oil at 150 or 300 mg/kg diet	TGF-β, TNF-α, MYD88, TLR4	N	NS	**	ANT	[19]
I	Oregano aqueous extract at 400, 500, 600 and 700 mg/kg	IL-4, 1L-10	N	NE	**	IM	[23]
S	*Astragalus membranaceus*, and *Glycyrrhiza uralencis* at 150 mg/kg each	IL-1β, TNF-α, IL-6	N	**	NE	ANT	[56]
I	A blend (*Astragalus membranaceus* and *Codnopsis pilosula* extract) at 500 mg/kg	N	IL-6, IL-10, IFN-β, IFN-γ, and TNF-α	NE	**	IM and ANT	[147]
S	*Yucca* saponin (YSa), *Yucca* *schidigera* (YS), and *Quillaja Saponaria* (QS) at 500 mg/kg	IL-6, TNF-α	N	**	NE	ANT	[148]
S	Plant polysaccharides	IL-β, TNF-α	IL-4, IL-10	**	NE	ANT	[150]
I	Sanguinarine (from *Macleaya cordata*) at 0.7 mg/kg diet	TNF-α, IL-4	N	NE		ANT	[120]
S	*Ilicis Chinesis *folium extract (powder) at 0, 250, 500, and 1,000 mg/kg	N	IL-4, IL-2	**	NE	ANT	[168]
S	Oral solutions of Oregano EO, and *Macleaya cordata* extract, at (125 mL/1,000L)	TNF-α, IL-1β, IL-4, and IL-6	N	**	NE	ANT	[173]
I	Thymol and Carvacrol eucetic at 30 mg/kg	IL-6, TNF-α	IL-10	NS	NE	ANT	[173]
I	*Loncirae flos* and Turmeric extract at 0, 300, 500 g/t	IL-2, IL-8, TNF-α, TLR4, NF-κB, and MyD88	N	**	NE	ANT	[174]
S	Plant tannins from various sources: 68% (AT), 60% (CT), 73% (QT), and 50% (TT)	IL-6, IL-1β, TNF-α	IL-10	**	**	ANT	[175]
I	Fermented Chinese herbal residue at 5%	TNF-α, IFN-γ, 1L-1β, 1L-6	N	NE	NE	ANT	[176]

*I* Intestinal mucosa, *S* Serum, *ANT* Anti-inflammatory, *IM* Immunomodulatory, *N* None, *NS* Non-significant, ** Significant, *NE* Not evaluated, *IgA*, *IgG*, *IgM* Immunoglobulin A, G, M, *sIgA* Secretory immunoglobulin A, *EO* Essential oil, *AT* *Acacia mearnsii* tannin, *CT* *Castanea sativa* tannin, *QT* *Schinopsis lorenzii* tannin, *TT* *Caesalpinia spinosa* tannin, *TNF*-α tumor necrosis factor-α, *IFN*-γ Interferon-tau, *IL* Interleukins, *1L-1β* Interleukin beta 1, *TLR4* Toll-like receptor 4, *NF-κB* Nuclear factor-kappa B, *MyD88* Myeloid differentiation response 88

A blend of Chinese herbs exhibited both immunomodulatory and anti-inflammatory effects, likely due to its rich content of bioactive compounds, which increased sIgA content in the jejunal mucosa and boosted serum immunoglobulins [149]. Similarly, the immunostimulatory and anti-inflammatory effects of *Pulicaria jaubertii* extract [103] were attributed to its triterpenes content, which possesses strong anti-inflammatory properties [177]. In addition to these benefits, the bioactive substances in LEO, including monoterpenoids and monoterpenes [178], have been shown to increase proinflammatory cytokines such as IL-1β and IFN-γ [31]. This immunostimulatory effect enhances the bird’s defense mechanisms, although it also underscores the complex interplay between stimulation and regulation within the immune system. The efficacy of a polyherbal mixture in modulating immune responses is further illustrated by its ability to increase and decrease the mRNA expression of *IL-4* and *IFN-γ*, respectively, reflecting a nuanced impact on serum cytokine levels [154]. The presence of OAs, alkaloids, flavonoids, and terpenoids in these herbs contributes significantly to their anti-inflammatory properties, adding to the complexity of their effects [179].

Inflammation regulation through key signaling pathways, such as NF-κB, further highlights the role of phytobiotics. Extracts from *Terminalia chebula* [160] and *Galla chinensis* [180] mediate anti-inflammatory effects through this pathway. However, higher doses of *Galla chinensis* extract may induce undesirable gut inflammatory responses, highlighting the importance of dosage in the application of these phytobiotics. Similarly, the alkaloid sanguinarine demonstrates anti-inflammatory properties by blocking the NF-κB pathway, a critical regulator of inflammation [181, 182]. Fenugreek seed extract further exemplifies this immune balance by reducing the expression of proinflammatory cytokines and the activities of nitric oxide synthases (NOS) and inducible NOS (iNOS), potentially by maintaining a balance between Th1 and Th2 cytokines [183]. The immunomodulatory potential of phytobiotics is further demonstrated by *Enteromorpha polysaccharide* extracts, which stimulate the mRNA expression of cytokines and related signaling pathways in both intestinal mucosa and serum [10]. This suggests that these can activate the TLR4/MyD88/NF-κB signaling pathway, thereby exerting immunomodulatory activity. Also, oregano EO has been shown to upregulate Avian β-defensins (*AvBD1*) in the ileum, increase the expression of *TGF-β*, and enhance sIgA concentration in the jejunum and ileum, primarily by inhibiting TNF-α synthesis [20]. Interestingly, while ferulic acid increased the expression of proinflammatory factors, it did not affect anti-inflammatory factors, underscoring the complexity of plant phenolic compounds in stimulating beneficial immune responses [143]. Taken together, all these findings emphasize the diverse mechanisms through which plant-based additives exert their effects.

In conclusion, phytobiotics represent a promising avenue for balancing immunomodulatory and anti-inflammatory effects in broilers, potentially acting as immune regulators and optimizing growth efficiency. However, the challenges associated with dosage and combination of these additives require further investigation to fully harness their potential in poultry production. The integration of these findings into practical applications will depend on a deeper understanding of the mechanisms through which these compounds interact with the avian immune system, ensuring that their use contributes to sustainable and efficient poultry farming practices.

### Effects of phytobiotics on gut barrier function

Research indicates that phytobiotics significantly enhance intestinal epithelial barrier function by modulating key molecules essential for maintaining mucosal integrity, such as mucins, tight junction proteins (TJPs), and endotoxins. Supplementation of *Pulicaria jaubertii* notably increased *MUC-2* expression in the intestinal mucosa, emphasizing its role in regulating tight junctions [103]. Similar benefits were observed with thymol and carvacrol eutectic, which significantly boosted mucin content in the ileum [173], although a phytogenic blend showed no such effect [184]. *Pueraria* extract increased the expression of the *MUC-2*, while curcumin had no effect [108]. Notably, while plant extracts like tannins offer gut protection, their effectiveness on TJP expression varies, potentially explaining differential susceptibility to enteric infections among different groups fed tannin [175]. *Dendrobium officinale* leaves increased the mRNA expressions of tight junction proteins throughout the small intestine, particularly in the duodenum [156].

Furthermore, *Glycyrrhiza glabra* (licorice) extract and a blend of plant extracts (Curcuma and olive leaf extract) significantly increased the expression of *JAM-2*, a critical component of the tight junction complex [96, 185]. This effect is likely attributed to the flavonoid content in these extracts, which helps preserve the structure of the intestinal epithelial barrier. EOs also enhanced the expression of tight junction proteins in the jejunum at 21 d of age, but lower levels of expression were observed at 48 d of age, the decline in expression at 48 d suggests a time-dependent effect, potentially due to initial adaptive responses or changes in the gut environment [152]. Moreover, the oral administration of carvacrol EO for two weeks has been shown to enhance the expression of key tight junction proteins such as *Occludin*, *Claudin-1*, *Claudin-5*, *ZO-1*, and *ZO-2* in the intestinal mucosa [186]. A synergistic effect was observed with xylooligosaccharides and gamma-irradiated *Astragalus* polysaccharides, resulting in significant levels of protein expressions in the jejunum, number of goblet cells, and a substantial reduction in serum D-LA levels [26], indicative of improved intestinal barrier function.

The beneficial effect of phytobiotics on intestinal permeability is further highlighted by the use of the *Quillaja* and *Yucca* (QY) blend, which reduced serum levels of fluorescein isothiocyanate-dextran (FITC-d), an indicator of intestinal permeability [101]. Additionally, polysaccharides derived from *Astragalus membranaceus* and *Glycyrrhiza uralensis*, were found to lower levels of DAO in the serum but did not affect D-LA concentrations [56], whereas algae-derived polysaccharides effectively reduced the serum levels of both D-LA and DAO [161]. These reductions are significant as they suggest enhanced mucosal integrity and a strengthened intestinal barrier.

In a similar vein, plant extracts such as Chinese herbal mixture extract [149] and a combination of *Lonicerae flos* and turmeric extract [174] reduced the levels of endotoxin and DAO in the blood. The positive correlation between the levels of DAO and endotoxin may explain the simultaneous reduction of both substances [187]. Also, GC tannin reduced endotoxin concentration in the serum [159]. The study of Ding et al. [152] reported that EO (containing thymol and carvacrol and carrier was rice husk powder and silica) reduced the concentration of DAO in the serum at 21 d of age but a significant increase in the levels was found at 48 d, the protective effect of EO may be efficient at early growth phase but not at a later phase, suggesting its effectiveness may be dose and time-dependent, and the composition of the EOs may also be a contributory factor. Beta-sitosterol reduced plasma DAO activity and D-lactate levels at both 14 and 21 d of age, irrespective of the inclusion dosage, supporting the early stages of growth [142]. The reduction in the levels of intestinal permeability indicators is favorable for preserving the integrity of intestinal mucosa. The findings reveal variability in efficacy due to variety, dosage level and time of exposure.

**Fig. 2:**
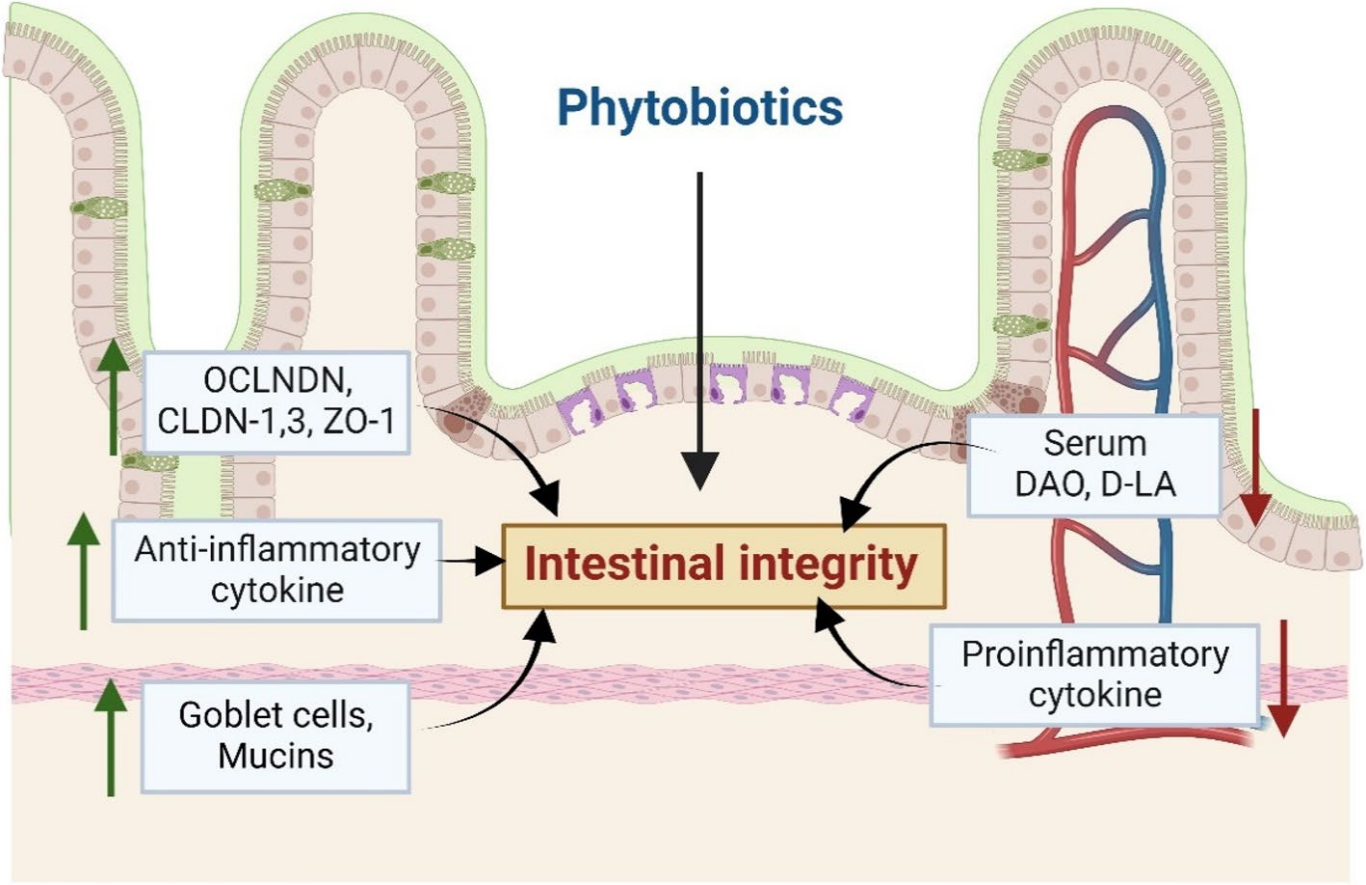
The mechanism by which phytobiotics maintain gut epithelium integrity. Created with BioRender.com

Nonetheless, these findings collectively underscore the potential of phytobiotics to enhance gut barrier function through multiple mechanisms, including the regulation of TJPs, modulation of gut hormone expression, and reduction of serum endotoxin levels, as illustrated in Fig. 2.

### Effects of phytobiotics on gut morphology

Research on broiler chickens has consistently highlighted the beneficial impact of phytobiotics on gut morphology, particularly on villi structure. Various studies have demonstrated that dietary supplementation with specific phytobiotics can significantly enhance VH and the VH/CD ratio, which are critical indicators of gut health and nutrient absorption efficiency, as listed in Table 4.

**Table 4:** Effect of phytobiotics on intestinal morphology of broiler birds

Diet	VH			CD			VH/CD ratio			References
	D	J	IL	D	J	IL	D	J	IL	
*Enteromorpha prolifera* polysaccharide at 400 mg/kg diet	NS	**	**	NS	**	**	**	**	**	[10]
POS (pectin oligosaccharide; 80 mg/kg Zn + 482 mg/kg) and Zn-POS chelate (80 mg/kg)	**	**	NS	**	**	NS	**	**	NS	[25]
Xylooligosaccharides (XOS; 100 mg/kg) and gamma-irradiated *Astragalus* polysaccharides (600 mg/kg)	**	NS	**	NS	NS	NS	NS	NS	**	[26]
Microencapsulated turmeric by maltodextrin: 1, 2, or 3 g/kg diet	**	**	**	NS	NS	NS	**	**	**	[34]
Polysaccharides: *Astragalus membranaceus* (300 mg/kg), or *Glycyrrhiza uralencis* (150 mg/kg)	**	**	**		NS	NS	**	**	**	[56]
EO (basil, caraway, laurel, lemon, oregano, sage, tea, and thyme) at 100 mg/kg	**	**	NS	**	NS	NS	NS	NS	NS	[84]
Sanguinarine (from *Macleaya cordata*) at 0.7 mg/kg diet	**	**	NS	NS	**	**	**	**	**	[120]
*Dendrobium officinale* leaves at 0, 1%, 5% or 10%	**	**	**	**	NS	NS	**	**	NS	[156]
*Terminalia Chebula* extract at 0, 200, 400 or 600 mg/kg	**	**	**	**	NS	NS	**	NS	**	[160]
Ethanol extract of elecampane (*Inula helenium *L.) rhizome at 250, 500 or 1,000 mg/kg	NE	**	**	NE	NS	**	NE	**	**	[163]
Anthocyanin-rich roselle (*Hibiscus sabdariffa* L.) extract at 50, 100, 200 or 400 mg/kg	**	**	**	NS	NS	**	**	NS	**	[164]
*Ilicis chinesis* folium extract (powder) at 0, 250, 500, or 1,000 mg/kg	**	NS	**	**	**	**	**	**	**	[168]
Combination of 100/200 mg of CEO (cinnamon EO) and 16.7/33.3 mg of BLF (Bamboo leaf flavonoid)/kg	NS	NS	NS	**	NS	NS	NS	NS	NS	[172]
Fenugreek extract at 50 or 100 mg/kg	NE	**	NS	NE	NS	**	NE	**	**	[183]
Plant extract (curcuma, chamomile, licorice and olive leaf) at 500–1,000 mg/kg	**	**	**	**	NS	NS	**	**	**	[185]
Blend of oregano essential oil (OEO) and GLM (glycerol monolaurate) at 0, 0.15%, 0.45%, or 0.75%	**	**	**	**	**	**	NE	NE	NE	[188]
Coconut husk extract at 100, 400 or 700 ppm	NS	NS	**	**	NS	**	**	NS	**	[189]

*NS* Non-significant, ^**^ Significant, *NE* Not evaluated, *EO* Essential oil, *OA* Organic acid, *VH* Villi height, *CD* Crypt depth, *D* Duodenum, *J* Jejunum, *IL* Ileum

For example, diets enriched with spent tea leaves and mannan-oligosaccharides increased the duodenal VH/CD ratio, likely due to the hydrolysis of oligosaccharides into SCFAs which serve as energy sources for enterocytes [190]. Likewise, a polyherbal mixture containing various herbs significantly enhanced the jejunal VH and VH/CD ratio [154], underscoring the potential of combined phytobiotics to amplify positive outcomes on gut health. Moreover, the synergistic effects of blended phytobiotics have shown even more promising results. Combinations of herbs such as green tea, Ashwagandha, ginger, black seed, and licorice, as well as mixtures like *Aerva lanata*,* Piper betle*,* Cynodon dactylon*, and *Piper nigrum*, have been reported to significantly improve villi structure compared to their individual components [15]. The combination of probiotics and *Pulicaria gnaphalodes* increased duodenal and ileal villi morphometrics, likely due to probiotic effects providing additional energy to intestinal cells [104]. Also, using pectin oligosaccharides with zinc enhanced villi morphology, likely through the fermentation of these oligosaccharides into butyrate and acetate, which are known to support gut health [25]. These findings suggest that the bioactive compounds in these blends work in concert to enhance gut morphology, likely through mechanisms involving SCFA synthesis and the promotion of epithelial cell proliferation. The significant enhancement effects of plant extract blend on gut morphology and integrity, regardless of the cereal type, suggest the promoting effect of the bioactive compounds in the blend [185].

Similarly, supplementation with *Forsythia suspensa* extracts improved villi morphometrics across all segments of the small intestine [146], a result that can be linked to its bioactive compounds such as phillyrin, forsythialan A, and phillygenin, which are known to promote lymphocyte proliferation and enhance the intestinal absorptive surface [191]. Also, *Yucca* saponin extracts were found to improve intestinal morphology, likely through their antimicrobial effects and suppression of gut inflammation [148]. Additionally, the inclusion of *Macleaya cordata* extract (MCE), into broiler diets, was observed to reduce CD and significantly increase the VH/CD ratio in the jejunum [192], suggesting its role in promoting intestinal integrity. Tannins from *Platyarya strohilacea Sieb. et Zucc* demonstrated a dose-dependent enhancement in jejunal VH and ileal VH/CD ratio [29]. Pueraria extract (PE) and curcumin enhanced villi morphology, with PE showing a more pronounced effect in the jejunum, probably due to low absorption and biotransformation of curcumin in the gut [108]. Basil oil also showed improvement in villi morphology, likely due to its content of methyl chavicol, an antioxidant that reduces tissue damage and enhances villi development [33].

The positive influence of phytobiotics extends beyond VH and CD. Various studies have reported enhancements in the surface area, width, length, weight, and density of intestinal villi, along with an increase in the number of goblet cells, which are crucial for mucus production and gut barrier function. For example, birds fed with gum arabic from *Acacia Senegal* exhibited increased villi surface area and width while simultaneously reducing CD, suggesting a protective effect on the gut mucosa [75]. Additionally, the administration of fennel essential oil was found to increase the width and surface area of ileum villi while reducing the muscular layer and lamina propria thickness [100], which could enhance nutrient absorption efficiency. Safflower oil, rich in polyunsaturated fatty acids, improved intestinal histology, mucosal thickness, goblet cell count, and lymphocytic infiltrations, indicating its potential to support gut health through anti-inflammatory mechanisms [193].

The synthesis of SCFAs due to gut fermentation of these plant products provides energy to villi enterocytes and promotes epithelial cell proliferation to support overall intestinal integrity. Additionally, a blend of essential oils like thyme, peppermint, and eucalyptus in drinking water [133], *Macleaya cordata* extract (MCE) and OEO [194], combinations of organic acids and EOs [195], or sesame bioactive peptide with savory and thyme oil [196], have demonstrated positive results in enhancing villi morphology in both jejunum and ileum. Organic acids and EOs also improved villi morphology by reducing gut inflammation and lesion scores [21]. Nevertheless, vegetable oil and quercetin did not improve villi morphology, possibly because the diets may not increase fatty acid metabolism, which can synthesize saturated fatty acids for epithelial gut cell proliferation [197].

**Fig. 3:**
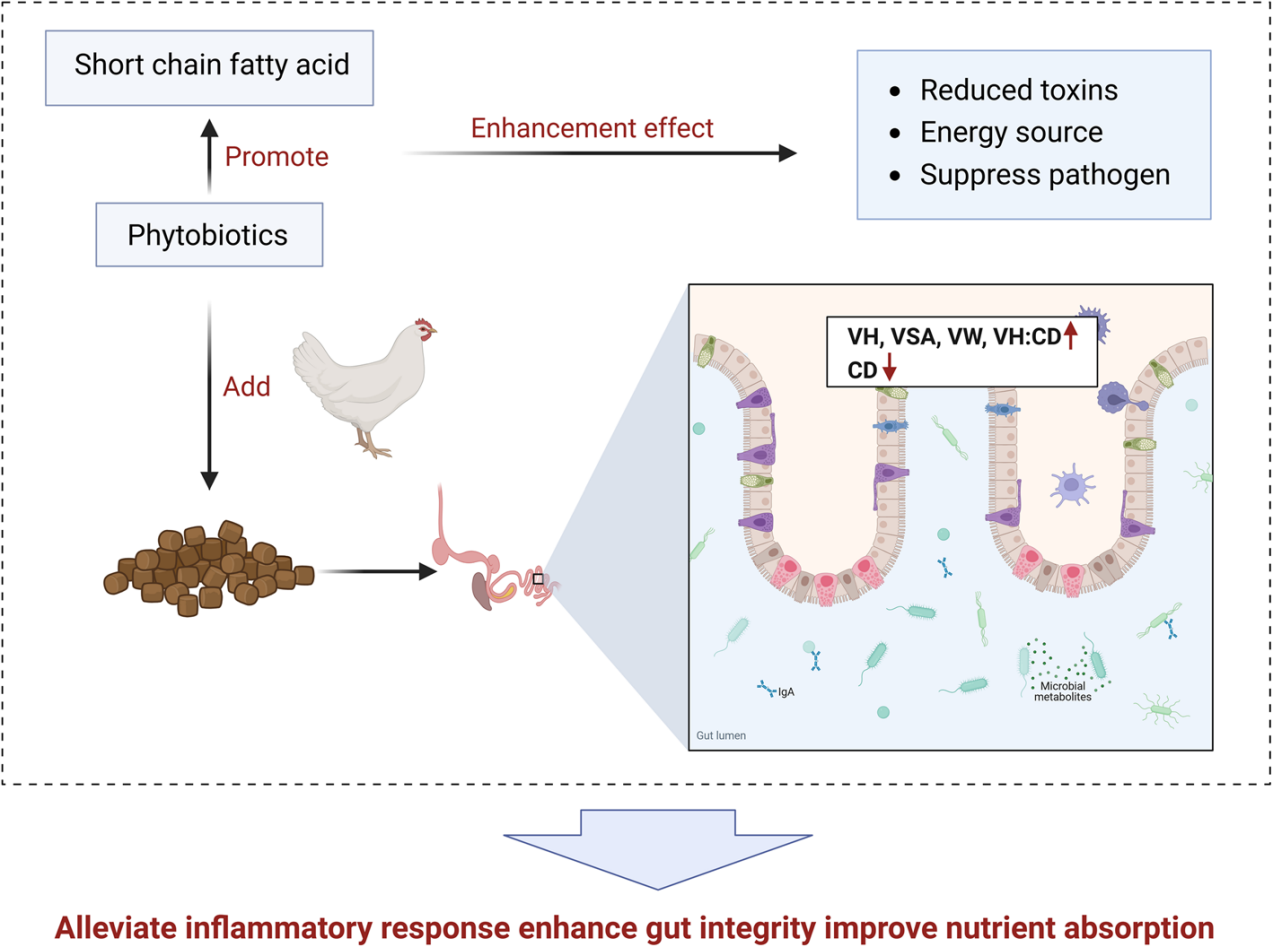
The mechanism of phytobiotics on gut villi morphology. Created with BioRender.com

In conclusion, the use of phytobiotics, particularly those that promote the synthesis of SCFAs, along with strategic combinations of various phytobiotics as a single feed additive, shows great promise in improving gut morphology and overall intestinal health in broiler chickens. These improvements not only support gut integrity but also enhance nutrient absorption and feed efficiency, which are crucial for the optimal performance and health of poultry, as presented in Fig. 3.

### Effects of phytobiotics on gut microbiota composition

Phytobiotics play a pivotal role in modulating gut microbiota composition, thereby enhancing gut health, host performance, and overall well-being. These plant-based compounds target key areas such as microbiota diversity, nutrient metabolism, immune response, and antimicrobial activity, each contributing to a holistic improvement in gut health. By promoting the proliferation of beneficial microbes while suppressing pathogenic species, phytobiotics offer a sustainable alternative to antibiotics, supporting both animal health and production efficiency. The success of these mechanisms depends on increasing beneficial microbes while reducing pathogens, as highlighted in various research studies (Table 5).

**Table 5:** Effect of phytobiotics on gut microbiota composition

Diets	Increased microbes	Reduced microbes	References
Blend (powder; ginger, liquorice, ashwagandha root, black seed and green tea leaves) at 3 kg/ton	*Lactobacillus*	*Coliforms*	[15]
OA (200 mg/kg), EO (150 mg/kg) and OA blend plus coated EO	*Lactobacillus*	*E. coli*,* Salmonella*,* Clostridium perfringens*	[21]
Ginger root extract at 1.5% or 3%	*Lactobacillus*,* Bifidobacterium*	*E. coli*	[22]
Oregano aqueous extract at 400, 500, 600 and 700 mg/kg	Firmicutes,* Lactobacillus*	*E. coli*	[23]
Microencapsulated turmeric by maltodextrin: 1, 2, or 3 g/kg diet	*Lactobacillus*	*Coliform*	[34]
0.1%–0.5% of olive leaf extract	*Lactobacillus*,* Bifidobacterium*	*E. coli*	[99]
*Forepia subpinata* powder at 1%, 2%, 3%	*Lactobacillus*,* Bifidobacterium*	*E. coli*,* Coliform*	[102]
*Pulicaria jauberti* powder at 0, 3, 6 and 9 g/kg	*Lactobacillus*	*Salmonella*	[103]
Blend ((wheat germ (powder), hops and grape seed (extract)): 0.05%, 0.1% or 0.2%	*Lactobacillus*	*E. coli*, *Salmonella*	[129]
Magnolol at 100–400 mg/kg	*Faecalibacterium*	*Coprobacillus*	[140]
A blend (*Astragalus membranaceus* and *Codnopsis pilosula* extract) at 500 mg/kg	*Bacteroidetes*,* unclassified Bacteroidales*	*Firmicutes* and *Alistipes*	[147]
EO at 200, 400 and 600 mg/kg (carrier: rice husk powder and silica)	*Lactobacillus*,* Faecalibacterium*	*Bacteroides*,* Intestinimonas*	[152]
Combined plant oil (eucalyptus, carvacrol, cinnamyl aldehyde) at 100 g/ton	*Streptococcus*,* Bifidobacterium*	*Escherichia-Shigella* and *Erysipelatoclostridium*	[198]
Plant extract oil at 200 or 400 mg/kg	*Faecalibacterium* and *unclassified Rikenellaceae, Lachinospiraceae*	*Lactobacillus*	[199]
Thyme extract at 150 mg/L	*Lactobacillus*	*Coliforms*	[200]
*Epimedium* extract at 200 mg /kg	*Lactobacillus*,* g_NK4A214_group*	*Microbacterium*, *Bacteroides*,* Gallibacterium*	[201]
Clove powder and Tulsi extract via water at (0.5% + 2%), (1% + 3%), and (1.5% + 4%)	*Lactobacillus*	*E. coli*	[202]

*EO* Essential oil, *OA* Organic acids, *E*. *coli* *Escherichia coli*, *S.* *aureus* *Staphylococcus aureus*

#### Increasing microbiota diversity and richness

Phytobiotics have emerged as potent modulators of gut microbiota, influencing both diversity and richness, which are critical indicators of gut health. For instance, *Galla chinensis* extract has been shown to enhance microbial diversity by promoting Bacteroidetes over Firmicutes, a shift typically associated with improved gut health [180]. This modulation reflects the ability of phytobiotics to influence the composition of the gut microbiome positively. However, the effects may vary; tannins derived from *Galla chinensis*, for example, have been reported to reduce alpha diversity [159], likely due to their bacteriostatic properties [203]. This reduction highlights the dual nature of phytobiotics, where their antimicrobial effects can sometimes lead to decreased microbial richness, as observed in studies where the pathogen inhibition properties of Chinese herbal medicine (CHM) contributed to a decline in microbial richness in birds [149]. Nevertheless, there are some phytobiotics that do not exert any pronounced effect on the gut microbiota composition. Herbal mixtures containing ginseng and artichoke, for example, did not significantly alter the microbial richness or diversity in birds [204], suggesting that the impact of phytobiotics can vary widely depending on their composition and the specific gut environment they interact with. These findings underscore the complex and context-dependent effects of phytobiotics on the microbial ecosystem, where enhancing microbial diversity can be a key mechanism by which these compounds modulate gut health.

#### Modulation of nutrient metabolism

Beyond influencing microbial diversity, phytobiotics also play a significant role in modulating nutrient metabolism within the gut, thereby enhancing overall metabolic efficiency. Previous studies showed that Fenugreek seed extracts increased the abundance of *Bacteroides* which are closely linked to the metabolism of carbohydrates and polysaccharides [183], thereby improving the breakdown and assimilation of these nutrients. Similarly, the administration of dietary supplements such as *Dendrobium officinale* leaves and Chinese herbal medicine (CHM) has been associated with an increase in the populations of Firmicutes and Bacteroidetes [149, 156], two bacterial phyla integral to nutrient utilization. An enhanced ratio of Firmicutes to Bacteroidetes is particularly noteworthy, as it has been correlated with weight gain in birds, a phenomenon observed with the administration of various plant extracts, including polysaccharide extracts [10, 56], sanguinarine [182], and low-bush blueberry pomace [205]. This increase in carbohydrate-hydrolyzing bacteria is key to improving feed efficiency and promoting weight gain [206], a positive trait for the broiler industry.

Furthermore, the inclusion of a blend of EOs and OAs in the diet promotes the abundance of Clostridiales, a bacterial order positively correlated with weight gain and efficient nutrient absorption [195]. Plant oils have been shown to increase the abundance of Bacteroidetes, the genera *Alistipes*, and *unclassified Rikenellaceae* [81], which are linked with lipid metabolism. OEO increased *Clostridium sensu stricto-1*, and *Lactobacillus* [19], which protects intestinal integrity due to its capacity to produce SCFA and supply energy to the intestinal cells [207]. Fenugreek seed extract augments cellulolytic bacteria, improving starch and sucrose metabolism pathways [183], while berberine enhances beneficial microbes linked to DNA replication and metabolism, potentially boosting growth performance [208]. Firmicutes and Bacteroidetes are closely related to nutrient metabolic pathways, where they facilitate the digestion and absorption of essential nutrients. Leveraging the notion that phytobiotics not only to maintain gut health but also to enhance metabolic efficiency, ultimately contributing to improved animal performance.

#### Modulation of immune and inflammatory response

In addition to their effects on nutrient metabolism, phytobiotics also modulate the immune and inflammatory responses in the gut. Extracts of *Lonicerae flos* and turmeric elevated microbial richness, enriching pathways associated immune system and biosynthesis of secondary metabolites [174]. This microbial modulation is critical as it directly influences the host’s immune status. Polyherbal mixtures increased the abundance of *Oscillospira* and *Ruminococcus*, which are linked to elevated levels of sIgA and IL-4 levels [154], indicating a strengthened mucosal immune response. Furthermore, the fermentation by-products of tannins derived from *Galla chinensis*, particularly propionic acid, enhanced immune function while simultaneously reducing inflammatory markers. This effect is mediated through the elevation of beneficial bacteria like *Faecalibacterium prausnitzii*, a species renowned for its anti-inflammatory properties [159, 209]. Essential oils have been found to increase the abundance of *Blautia*, which is positively correlated with the production of branched-chain fatty acids, thereby reducing the expression of inflammatory genes in the duodenum [84]. Similarly, tannins increased the abundance of *Parabacteroides*, a bacterium known for their anti-inflammatory and immune-regulatory effects, further emphasizing the immune-modulatory potential of phytobiotics [29]. Additionally, dietary inclusion of *Ampelopsis grossedentata* extract increased the abundance of *Succiniclasticum* and *Akkermansia*, both of which are positively correlated with increased concentrations of IgA and IgG, markers of robust immune response [210]. However, extracts of fenugreek seeds reduce the abundance of bacteria such as* Synergistes*,* Campylobacter*, and* Lachnoclostridium* [183], which are often associated with intestinal dysbiosis [211]. This reduction highlights the selective modulation of gut microbiota by phytobiotics, targeting harmful bacteria while promoting the growth of beneficial ones. Moreover, *Glycyrrhiza uralensis* and *Astragalus membranaceus* extracts have demonstrated inhibitory effects on the proliferation of *Bacteroides* and *Desulfovibrio*, resulting in reduced endotoxin production and lower levels of inflammatory cytokines [41, 181]. These microbial-immune interactions are essential not only for maintaining gut integrity but also for optimizing nutrient utilization. The interplay between gut microbiota and the host immune system is crucial for maintaining gut integrity and optimizing nutrient utilization, with phytobiotics playing a central role in this complex interaction.

#### Generation of antimicrobial effects

Finally, the antimicrobial effects of phytobiotics are a critical aspect of their role in gut health. These effects are manifested through the increased proliferation of beneficial microbes and the inhibition of pathogen growth. The antimicrobial effects of phytobiotics reflect increased beneficial microbes and inhibition of pathogen proliferation, as presented in various studies in Table 5.

LEO significantly reduces *E. coli* and coliforms in the ileum and cecum [153]. Similarly, oregano and phytogenic blend EOs reduced *E. coli* and *Salmonella* in both ileum and cecum [14, 101]. In contrast, a similar reduction in the ileum was observed with EER and cinnamon oil [123, 136]. Also, fermented grape seed reduced *Staphylococcus aureus* significantly, although it did not affect *E. coli* [13]. Synergistic antibacterial effects were notable with plant extract blend [159], EO blend in drinking water [103], encapsulated EO and OA [183], and sesame meal bioactive peptides in combination with EO [142]. The antibacterial effects of these plant products are likened to the increased proliferation of *Lactobacillus*, which aids in the competitive exclusion of pathogens. Also, it may be due to antimicrobial attributes of bioactive compounds present in phytobiotics, as discussed in the earlier section (An overview of phytobiotics). For instance, linalyl acetate and linalool in lavender EO impair cell permeability and homeostasis, leading to bacterial cell death [185, 186]. Further examples revealed the increased presence of beneficial bacteria such as Streptococcus in Xufeng black bone hens [29], which produce bacteriocins or hydrogen peroxide, conferring antibacterial benefits [175]. The capacity to increase beneficial microbes, modulate nutrient metabolism, regulate immune response, and reduce pathogen load suggests that phytobiotics can naturally and sustainably replace antibiotics, regulating microbiota-host relationships holistically.

### In vitro research studies

In various in vitro research studies, the antimicrobial efficacy of EOs and other bioactive compounds has been extensively investigated, demonstrating significant inhibitory effects on various pathogens. For instance, Ding et al. [152] found that the inclusion of EOs significantly inhibited the growth and biofilm formation of avian pathogenic *Escherichia coli* O78 and *Salmonella pullorum*. Similarly, *P*. *gnaphalodes* EO exhibited potent antibacterial activity against *Salmonella typhimurium*,* E. coli*, and *Mycobacterium tuberculosis.* The major bioactive compounds hydrocarbon monoterpenes (e.g., α-pinene, cymene, and terpinene), alcohol monoterpenes (e.g., 1,8-cineole, α-terpineol, terpinene-4-ol), and phenolic compounds like thymol, contribute to its high antimicrobial and antioxidant activities [212, 213]. Likewise, Kollanoor-Johny et al. [214], reported that using a modified cecal medium, several EO compounds; *trans*-cinnamaldehyde, eugenol, thymol, and carvacrol, were found to be very effective against *Salmonella* and *Campylobacter* in commercial broiler and layer chickens. The significant reduction of the population of the pathogens was stronger with *trans*-cinnamaldehyde and eugenol, thus emerging as potential key effective bactericidal agents. Furthermore, a phytobiotic mixture containing thymol, menthol, linalool, *trans*-anethole, methyl salicylate, 1,8-cineole, and p-cymene was evaluated for its antibacterial activity against selected strains of *Salmonella* spp., showing effectiveness against antibiotic-resistant strains of *Salmonella enterica* serovars Enteritidis, Typhimurium, and Kentucky [5]. The lipophilic nature of these bioactive constituents, such as menthol and 1,8-cineole, inhibits bacterial efflux pumps and ATP synthesis, leading to impaired cellular functions. Additionally, linalool has been reported to interfere with protein synthesis by binding to bacterial ribosomes, thereby inhibiting the translation of essential proteins. Also, fermentation in vitro together with oral administration or oregano aqueous extracts, increased the mRNA expression levels of *MUC2* and secretion of sIgA, increased LAB and suppressed pathogens, the improved mucosal immunity could be attributable to its higher content of acetic acid [23]. Forsythiaside A, a major component in Forsythia suspensa, has been shown to inhibit LPS-induced inflammatory responses in BV2 microglial cells and primary microglial cells through the inhibition of *NF-κB* activation and activation of the Nrf2/HO-1 signaling pathway [215]. Oregano aqueous extract was found to directly exert strong antibacterial activity reducing the abundance of microbes involved in toxin production and causing gut dysbiosis [216]. In conclusion, phytobiotics offer a compelling natural alternative to synthetic antibiotics, enhancing gut health by modulating microbiota composition, nutrient metabolism, immune response, and antimicrobial activity. In vitro studies further validate their efficacy, demonstrating their ability to disrupt harmful bacteria and reduce reliance on antibiotics. The precise mechanisms, such as cell membrane disruption and inhibition of DNA and protein synthesis, offer valuable insights that can inform in vivo studies. The benefit of these in vitro studies lies in their ability to identify effective compounds, optimize dosages, and explore synergistic interactions before advancing to in vivo applications, ultimately paving the way for more.

Building on the positive effects of phytobiotics on gut health; specifically, their enhancement of gut morphology, antioxidant function, gut barrier integrity, and the modulation of gut microbiota, immune, and inflammatory responses. The synergistic enhancement of gut morphology, antioxidant function, and gut barrier integrity by phytobiotics not only fortifies the physical structure and defense mechanisms of the gastrointestinal tract but also cultivates a more balanced and resilient gut microbiota. This harmonious interaction between the gut and its microbial inhabitants optimizes the immune and inflammatory responses, reducing oxidative stress, allowing the broilers’ energy to be more focused on growth rather than on combating stressors. Thus, creating a conducive environment for improved nutrient utilization which translates into better feed conversion ratios, leading to enhanced growth performance. Consequently, these integrated effects contribute significantly to the overall growth performance and health of broilers, highlighting the multifaceted role of phytobiotics in poultry nutrition. For the poultry industry, this translates to improved animal health, better feed efficiency, and enhanced production performance. By fostering beneficial microbes and optimizing gut functions, phytobiotics can sustainably boost productivity while addressing the growing concern over antibiotic resistance.

## Benefits of phytobiotics in broiler nutrition: implications for nutrient utilization and growth performance

Building on the positive effects of phytobiotics on gut health, this section highlights the significant impact of phytobiotics, including essential oils, plant extracts, herbs, and spices on nutrient utilization and growth performance. The translation of better gut health to improved nutrient utilization and growth performance (measured in terms of weight gain and feed conversion ratio), due to dietary phytobiotics from our key 100 studies are presented in Additional file 1. These natural feed additives, rich in bioactive compounds, have been shown to positively influence weight gain, feed efficiency, and nutrient absorption in broiler chickens through various mechanisms, including the stimulation of digestive enzymes and modulation of appetite-regulating pathways.

The incorporation of bioactive compounds from various plant-based supplements in animal nutrition has shown substantial promise in enhancing weight gain and feed efficiency. For instance, LEO, rich in linalool, has been found to have a notable impact on weight gain, likely due to its enzyme-stimulating and appetite-enhancing properties [217]. EOs (thymol, carvacrol, and cinnamaldehyde) enhanced growth performance by restoring microbiota balance, which in turn optimized nutrient absorption [11]. In the case of gum arabic (*Acacia senegal*), its fermentation in the cecum produces propionic acid, a compound that promotes gut health and nutrient uptake, contributing to increased weight gain and improved feed conversion ratio (FCR) [75]. The bioactive substances in cinnamon oil, such as cinnamaldehyde and eugenol, are known to stimulate endogenous enzymes, further enhancing weight gain and feed efficiency [151]. *Pulicaria jaubertii* powder, with its richness in fatty acids and phenolic compounds, has been found to improve feed efficiency and weight gain, potentially by enhancing gut motility through the increased secretion of endogenous enzymes [103]. The beneficial effect of tulsi and clove powder on growth performance may be attributed to the impact of trace elements in cloves [218], reduced amino acid degradation [219], while tulsi’s gastroprotective and immunomodulatory properties [125] further support these benefits. Steroid saponins in fenugreek seed [158] and yucca extracts [120] play a crucial role in enhancing weight gain and feed efficiency by stimulating nutrient digestion and absorption. Likely due to their interaction with key endogenous molecules, such as ghrelin, neuropeptide Y (NPY), and Agouti-related protein (AgRP) in the hypothalamus, which is essential for hunger stimulation and feed intake [220, 221]. Additionally, extracts from *Lonicerae flos* and turmeric, rich in chlorogenic acid and curcumin, respectively, promote weight gain due to their anti-inflammatory and antioxidant properties [174], highlighting the importance of natural dietary interventions in improving animal growth performance by targeting multiple physiological pathways.

Moreover, phytobiotics, particularly EOs and their combinations, have demonstrated significant roles in enhancing nutrient digestibility, thereby promoting weight gain in broiler birds. The combination of glycerol monolaurate and oregano EO improved growth performance, primarily by enhancing the apparent digestibility of essential amino acids, which are crucial for regulating metabolic pathways [188]. Similarly, dietary blends of OAs and EOs have been reported to improve the apparent ileal digestibility of dry matter (DM), crude protein, ether extract (EE), and apparent metabolizable energy (AME) [21], while EO further supports these findings and boosted sucrase [11]. Additionally, the ethanol extract of elecampane rhizome (EEER) enhances the digestibility of DM, organic matter (OM), and GE, although it reduced protein digestibility due to its protein-binding properties [163], suggesting the need for further research into better sources, optimal dosage and extraction method. Encapsulated additives, such as those containing capsicum mixed with black pepper and ginger extracts, have shown improved digestibility of DM, GE, and crude protein [145], likely due to their slow release along the intestine. Enhanced secretion of enzymes such as trypsin, chymotrypsin, lipase, and amylase, leading to better nutrient utilization were significant in broiler birds fed natural EO [19] and combined essential oils and saponins [122]. However, the growth-promoting effect of plant oils combined as a single feed additive was comparable to the control and Antibiotics groups, although no adverse effect was found [38]. Therefore, the action of phytobiotics on digestive enzymes plays a critical role in promoting nutrient digestibility, ultimately contributing to improved weight gain in broilers.

The synergistic effects of various phytobiotics, when combined, have been shown to significantly enhance weight gain and improve overall growth performance in poultry. The combination of xylooligosaccharides with gamma-irradiated *Astragalus* polysaccharides positively influenced villi structure and intestinal barrier integrity, leading to enhanced weight gain [26]. Sesame seed bioactive peptides, when combined with EOs, improve production performance traits, likely due to the enzyme-stimulating activity of the EOs, which enhances the functionality of sesame meal in the gastrointestinal tract [196]. Probiotics such as Protexin, when used alongside plant extracts like Gunnera, exhibit synergistic effects on feed efficiency and growth performance [222], likely due to probiotic effect in the gut. Previous report established a positive correlation between weight gain and the abundance of *Lactobacillus* in the gut [223]. Additionally, a herbal mixture containing Ginseng and Artichoke has been shown to result in weight gain [204], attributed to ginsenoside Rg1’s ability to preserve gut morphology, maintain intestinal integrity, and suppress pathogens, along with the antioxidant, anti-inflammatory, and digestive benefits of these herbs [224, 225]. In another study, broilers fed a polyherbal mixture showed tremendous increases in weight gain and decreases in FCR, possibly due to the diverse bioactive substances present in the herbs with therapeutic properties [126].

The findings from various studies suggest that the positive effects on growth performance associated with phytogenic blends and EOs can be attributed; to the presence of natural antioxidants that slow intestinal motility and protect against oxidative injury [129], and presence of thymol, carvacrol, and terpinene, which are known to stimulate bile acid and pancreatic enzyme secretion (amylase, lipase, and protease) [226], and carvacrol is an essential appetite stimulant in the hypothalamus [227]. Furthermore, the growth-promoting effects of EOs, herbs, and plant extracts are mainly due to their enzyme-stimulating aromatic compound content.

In conclusion, plant-based supplements and EOs in animal nutrition present a promising approach to enhancing weight gain and feed efficiency. The bioactive compounds in these natural products target multiple physiological pathways, including enzyme stimulation, microbiota balance, and nutrient absorption, leading to improved growth performance. The synergistic effects observed with combined phytobiotics underscore their potential as effective dietary interventions in poultry production. As research progresses, these natural strategies may offer sustainable and efficient alternatives to conventional growth-promoting methods in animal agriculture.

## Challenges and limitations of phytobiotics use in broiler nutrition

The use of phytobiotics in broiler nutrition is an area of growing interest due to their potential to enhance growth performance, improve gut health, and reduce the reliance on synthetic antibiotics. However, several challenges and limitations must be addressed to fully harness their benefits. A significant challenge is the variability in the effects of phytobiotics, which can be attributed to factors such as the specific type of phytobiotic used, the dosage, the form in which it is administered, and the growth phase of the broilers. The study by Windsch et al. [228], attributed the varying results on the efficacy of phytobiotics in poultry performance to a lack of understanding of Phyto additive processing techniques. This variability is not just a matter of scientific curiosity but has direct implications for broiler production.

Taking cognizance of these variables would provide insights into optimizing these additives, boosting their benefits, and reducing broiler health and productivity issues related to nutritional interventions.

### Nature of phytobiotics and form of delivery

Tannins, widely recognized for their potent antioxidant properties, have been extensively studied for their potential to contribute to disease prevention in poultry. However, their application is not without complications. The bitterness and protein-binding capabilities of tannins can impair protein digestibility and reduce mineral absorption, leading to decreased feed intake and growth performance [229, 230]. Additionally, high levels of tannins may irritate the mucosal lining of the gastrointestinal tract and disrupt microbial populations, further complicating their role in broiler nutrition. Consequently, the use of tannins in broiler diets requires careful calibration to strike a balance between their antioxidant benefits and their potential negative impacts on growth and feed efficiency, which are critical for the profitability of broiler production.

Similarly, EOs, another prominent class of phytobiotics, are valued for their strong antimicrobial properties, making them appealing as natural alternatives to antibiotics [231]. However, the effectiveness of EOs can be compromised by their volatility and sensitivity to environmental factors such as heat and light, leading to reduced oxidative stability, thermostability, and biological potency [232]. In addition to tannins and EOs, other phytobiotics such as gum arabic and specific plant extracts also demonstrate both beneficial and limiting effects. Gum arabic, due to its highly viscous and insoluble nature, can reduce feed intake by affecting the gut passage rate [75]. These limitations pose challenges in maintaining the consistent efficacy of these plant-derived products in broiler diets, necessitating advancements in formulation and delivery methods to optimize their benefits while minimizing variability in performance outcomes.

The variability of microbiota across different intestinal segments further complicates the bioavailability and efficacy of plant-based products [233]. *Macleaya cordata* extract increased microbial diversity in the jejunum and ileum, while effects in the cecum remained unaffected [192]. Berberine effectively modulates gut microbiota diversity and function [208], likely due to its limited systemic absorption and predominant action at the gastrointestinal tract surface [234]. The bioavailability of curcumin, another phytobiotic, is similarly influenced by its low absorption and biotransformation in the gut [235], leading to minimal impact on the expression of tight junction proteins in the jejunum [108]. These findings highlight the critical role of gut physiology and microbial populations in modulating the efficacy of phytobiotics, which can vary significantly depending on the segment of the intestine.

The choice of delivery route further confounds the application of phytobiotics in broiler production. For example, extracts of OEO and *Macleaya cordata* prepared into oral liquid and administered directly through waterline systems have demonstrated better regulatory effects on broiler performance [194]. However, water-based delivery systems may not be ideal, as some substances could adhere to water-pipe linings, reducing their absorption. Carvacrol, which is insoluble in water, is typically administered orally, which, while reducing feeding load, may result in uneven distribution when used in-feed, limiting its effectiveness [186]. Therefore, employing advanced technologies that preserve the stability of natural plant products is crucial for enhancing their utilization and consistency in broiler production. The use of cranberry and blueberry pomace or their ethanolic extracts, for instance, has shown variable effects on weight gain, likely due to differences in vegetative parts and bioactive compounds [236], underscoring the need for extraction methods tailored to each plant part.

Phytobiotics in broiler diets offer benefits but also present challenges due to their potential to impair nutrient absorption and alter gut microbiota. To maximize their advantages, careful management of their use is essential. Future research should focus on improving the stability, bioavailability, and delivery methods of these compounds. Additionally, understanding the interactions between phytobiotics, gut microbiota, and intestinal physiology will help develop more effective dietary strategies, ensuring both the health of broilers and the profitability of production.

### Variations in structure and functional groups

Phytobiotics, particularly EOs, exhibit diverse antimicrobial actions due to variations in their molecular structure and functional groups, which influence their hydrophilicity, polarity, and hydrogen-binding capacity [237]. These structural differences are critical in determining the efficacy of their antimicrobial properties. The carbonyl groups present in cinnamaldehyde allow it to bind to microbial proteins, inhibiting enzymatic activity and forming membrane pores [238]. Similarly, thymol and eugenol, which are also bioactive compounds found in plant EOs, disrupt microbial lipid membranes, altering their permeability and causing leakage of cellular components [239]. Notably, the antibacterial potency of cinnamaldehyde derived from cinnamon oil has been shown to be superior against *E. coli* compared to other EOs like those from clove bud or ajwain seed [240]. Additionally, carvacrol, a component of oregano oil, exhibits stronger antimicrobial effects than other plant phenolics, the efficacy of natural oregano oil, which contains multiple functional groups and aromatic compounds, surpasses that of synthetic versions composed solely of thymol and carvacrol [19], highlighting the importance of the natural synergistic interactions within blends [27].

The structural-functional group interactions of these phytobiotics are not only significant in understanding their antibacterial mechanisms but also have practical implications for their application in the broiler industry. By strategically utilizing these varied mechanisms, phytobiotics can be employed to target specific pathogens, thereby enhancing gut health and nutrient absorption in broilers. The synergistic effects observed in essential oil blends, particularly those containing carvacrol, offer a more potent antimicrobial activity than individual compounds. This makes them effective natural alternatives to synthetic antibiotics, which can contribute to improving growth performance and weight gain in poultry. Ultimately, leveraging the structural-functional interactions of phytobiotics offers a promising strategy for reducing reliance on antibiotics in poultry production, promoting more sustainable and health-conscious practices in the industry.

### Dosage levels and sensitivity

The impact of dosage levels on growth performance in broilers is a critical factor that varies depending on the specific additive and its concentration. For instance, the inclusion of LEO up to 460 mg/kg did not negatively affect growth performance, demonstrating that certain levels can be used safely without adverse effects [31]. In contrast, clove powder and tulsi extract showed a dose-dependent increase in performance, where moderate doses enhanced growth, but higher doses led to a reduction in feed intake and weight gain [174]. This decline in performance at higher concentrations can be attributed to the eugenol content in clove, which is known to inhibit gastrointestinal motility, potentially leading to compromised nutrient absorption and gastrointestinal issues [241, 242]. Feeding broilers graded levels of ginger extract showed varied effects on growth performance; a 1.5% supplementation improved weight gain and FCR, though not significantly different from the antibiotics group, but higher dosage inhibited growth, likely due to phytotoxicity effects impairing normal physiological functions [22]. Moreover, the inclusion of *Dendrobium officinale* leaves at different concentrations highlighted the importance of optimal dosage. About 1% inclusion improved growth performance indices, while a 5% inclusion produced results comparable to the control group, suggesting that moderate levels are beneficial. However, a 10% inclusion reduced average daily weight gain, likely due to an overload of certain bioactive components that may have disrupted nutrient absorption or metabolic processes [156]. *Galla chinesis* extract increased final body weight but at a lower concentration of 250 mg/kg [180], depicting the dosage inclusion level issue. Overall, these examples underscore the importance of dosage in determining the efficacy of feed additives. Moderate levels can enhance growth performance by improving nutrient utilization and promoting overall health, while higher doses may lead to adverse effects such as reduced feed intake, impaired nutrient absorption, and potential toxicity. Thus, determining the optimal dosage is essential to maximize the benefits of these additives while avoiding negative outcomes.

### Growth phases of birds

Diets supplemented with PE and curcumin did not affect feed intake or weight gain over 28 d [108]. Lavender EO at 300 or 600 mg/kg enhanced weight gain only during the finisher phase but not the starter phase [153]. Encapsulated EO and OA boosted growth performance during the starter phase, but no notable effects were found in the finisher phase, possibly due to the early modulation by gut microbiota [243]. Diets containing an encapsulated phytogenic blend of capsicum, black pepper, and ginger extract improved growth performance only during the first week, with no significant effects after that [145]. Possibly, the feeding duration was too short to elicit substantial changes in the concentration used. Supplementation of anthocyanin-rich roselle (*Hibiscus sabdariffa *L.) extracts at various doses increased villi development and improved growth performance at the starter phase only but had no significant effect on overall weight gain [164]. Although polyphenols are beneficial, they can reduce protein and amino acid digestibility by inhibiting pancreatic enzymes and reducing body weight [141]. The study by Yang et al. [172] found that cinnamon EO and bamboo leaf flavonoid had no significant impact on the growth performance of broilers.

The phase-specific effectiveness of phytobiotics in broilers is influenced by the birds’ physiological needs, gut microbiota interactions, and the mode of action of the bioactive compounds. During the starter phase, rapid growth demands enhanced nutrient absorption, making supplements like encapsulated EOs and OAs more effective, whereas the finisher phase focuses on fat deposition, where these benefits may diminish. Additionally, phytobiotics like polyphenols, while beneficial for their antioxidant properties, can inhibit digestive enzymes, reducing nutrient digestibility in later stages. Optimizing phytobiotics involves tailoring supplements to specific growth stages, using synergistic combinations, adjusting dosage and duration, and continuously monitoring performance to ensure sustained benefits throughout the broiler’s growth cycle.

In conclusion, while phytobiotics offer promising benefits in broiler nutrition, such as enhanced growth performance and improved gut health, their use is accompanied by significant challenges. The variability in their effects, influenced by factors like type, dosage, delivery method, and growth phase, complicates their consistent application. Additionally, issues like impaired nutrient absorption and altered gut microbiota underscore the need for careful management. To maximize the advantages of phytobiotics, future research should focus on optimizing their stability, bioavailability, and delivery methods, ensuring more consistent and effective outcomes in broiler production.

## Conclusion and future directions in phytobiotics research

The future of phytobiotics in animal nutrition holds promise, underlined by the need for precise formulation and innovative delivery methods to leverage their benefits maximally. Technological advancements, notably in microencapsulation and extraction techniques, have mitigated previous challenges associated with phytobiotics, expanding their applications in promoting animal health. For instance, new extraction techniques and refined dosage formulations have revealed that appropriate levels of tannin supplementation can significantly enhance animal performance [244, 245]. More recently, the study by Tong et al. [28] reported that optimal tannin levels can improve growth without compromising digestive enzyme secretion or intestinal absorption, countering the challenges of their bitter taste. Additionally, microencapsulated *Galla chinensis* tannins have been shown to enhance the antioxidant defense system of birds during early growth stages [246]. Further, substantiates the earlier findings on potentials of microencapsulation technology to improve the palatability and efficacy of tannins [247]. Likewise, nanoencapsulation techniques have been reported to enhance the bioavailability, oxidative stability, thermostability, and biological potency of essential oils [248]. For example, thyme essential oil encapsulated in chitosan nanoparticles [227] microencapsulated basil oil [33] has been shown to exhibit higher efficacy in improving the performance and physiology of the broiler birds significantly, compared to its free form. Thymol and carvacrol eutectic, a novel approach, has proven effective in maintaining the stability of volatile essential oils, leading to improved growth performance [173].

As research progresses, integrating phytobiotics with conventional feed additives, may pave the way for more sustainable, efficient, and health-promoting animal husbandry techniques. This integration emphasizes the need to understand molecular interactions and microbial diversity involved, highlighting the complexity and potentials of phytobiotics. Such tailored applications are becoming essential components of precision animal nutrition methods and nutrigenomics approaches. Moreover, the ongoing development of advanced delivery technologies, such as nanoencapsulation, holds promise for improving the stability and efficacy of phytobiotics, positioning them as both growth promoters and viable gut enhancers. This trend is likely to enhance food safety and consumer confidence, making it imperative to conduct further research into effective plant-based products for their successful integration into poultry practices.

As phytobiotics continue to gain traction, evolving regulatory requirements will ensure their safety and consistency, aligning with consumer demands for naturally produced and sustainable animal products. However, while the potential benefits of phytobiotics are significant, there are still concerns about their effects on physiological processes in the gut, with some compounds potentially causing inflammation due to irritation of the mucosal membrane. This underscores the importance of extensive toxicity assessments and ongoing research to determine the best uses of phytobiotics as antibiotic alternatives.

Our findings indicate a complex interplay between plant-based products and animal health, underlining the need for additional research to optimize their use. The studies reviewed in this paper demonstrate intricate relationship between the phytochemical properties of plant-derived products, their delivery techniques, and their biological impacts on animal health and performance. While challenges related to palatability, stability, uniform distribution and changes at molecular levels remain, novel techniques like nanotechnology and omics technology have shown great promise. As the strategic use of phytobiotics in animal feed evolves, it becomes increasingly evident that significant improvements in agricultural practices are possible, benefiting both productivity and animal welfare. Future research should concentrate on fine-tuning processing procedures, dosage levels, and distribution routes to promote health and productivity gains while reducing adverse effects to the barest minimum. Thus, it provides a pragmatic direction for comparing the optimal efficiency and suitability of different plant products for broiler production in this era of antibiotic-free diet. In the broiler industry, nutritional strategies integrating phytobiotics, alongside omics technology, are gaining prominence. Thus, focusing on the critical role of gene-nutrient-microbiota interactions within the framework of nutrigenomics and the microbiota-gut-brain axis could offer a more comprehensive understanding of their efficacy in broiler nutrition. Moreover, the relationship between phytobiotics and gut microbiota presents an exciting avenue for research in nutrigenomics. A deeper understanding of this relationship could drive innovations in dietary strategies that leverage these natural additives for enhanced health benefits.

This review has underscored the potential of phytobiotics as a sustainable alternative to synthetic antibiotics in broiler nutrition, highlighting their multifaceted benefits on gut health, immune response, and overall performance of broiler chickens. By integrating findings from recent studies, it is evident that phytobiotics enhance gut health via its impact enhancement effect on gut morphology, antioxidant and immune function, gut barrier integrity and function, gut microbiota, contributing to better growth performance, and reduced mortality without the adverse effects of antibiotic resistance and residues. EOs, rich in bioactive compounds, offer significant potential for broiler production from both performance and health perspectives, and the application of omics technology could further elucidate these benefits. Despite these promising advantages, challenges such as variability in efficacy, complexity in optimal dosage formulations, and the economic feasibility of integrating phytobiotics into large-scale poultry operations require further exploration. Future research should aim to standardize phytobiotics formulations, ensure their safety and consistency, and assess their long-term impacts on both animal welfare and the economic landscape of the poultry industry.

In conclusion, phytobiotics represent a viable and promising path toward antibiotic-free poultry farming, aligning with consumer preferences for naturally reared and healthier meat products. Continued advancements in this field will improve broiler health and productivity and significantly contribute to the sustainability and ethical standards of global poultry practices.

## Supplementary Information

**Additional file 1.** Effects of various phytobiotics on physiological responses and growth performance of broiler birds.

## Data Availability

The paper dataset generated for this review is available in an Excel file as a supplementary file.

## Abbreviations

AvBD1: Avian β-defensins
AJ: Adhesion junctions
CAT: Catalase
CD: Crypt depth
DAO: Diamine oxidase
D-LA: D-Lactic acid
EO: Essential oil
GIT: Gastro intestinal tract
GSH-Px: Glutathione peroxidase
IL: Interleukins
LAB: *Lactobacillus*
MDA: Malondialdehyde
MUC-2: Mucin-2
Nrf2: Nuclear factor erythroid 2-related factor 2
NF-κB: Nuclear factor-kappa B
RNS: Reactive nitrogen species
ROS: Reactive oxygen species
SOD: Superoxide dismutase
SCFA: Short chain fatty acid
sIgA: Secretory immunoglobulin A
TLRs: Toll like receptors
TNF-α: Tumor necrosis factor
TJ: Tight junction
VH: Villi height
VH/CD: Villi height to crypt depth ratio
ZO-1: Zonula occludens-1

## References

[1] Gadde U, Kim WH, Oh ST, Lillehoj HS. Alternatives to antibiotics for maximizing growth performance and feed efficiency in poultry: a review. Anim Health Res Reviews. 2017;18(1):26–45. 10.1017/S1466252316000207. 10.1017/S1466252316000207 28485263

[2] Ronquillo MG, Hernandez JCA. Antibiotic and synthetic growth promoters in animal diets: review of impact and analytical methods. Food Control. 2017;72(Part B):255–67. 10.1016/j.foodcont.2016.03.001.

[3] Diarra MS, Malouin F. Antibiotics in Canadian poultry productions and anticipated alternatives. Front Microbiol. 2014;5:282. 10.3389/fmicb.2014.00282. 24987390 10.3389/fmicb.2014.00282 PMC4060556

[4] Hur J, Kim JH, Park JH, Lee YJ, Lee JH. Molecular and virulence characteristics of multi-drug resistant *Salmonella* Enteritidis strains isolated from poultry. Vet J. 2011;189(3):306–11. 10.1016/j.tvjl.2010.07.017. 20822940 10.1016/j.tvjl.2010.07.017

[5] Iwinski H, Wódz K, Chodkowska K, Nowak T, Rózanski H. In vitro evaluation of antimicrobial effect of phytobiotics mixture on *Salmonella* spp. isolated from chicken broiler. Antibiotics. 2022;11(7):868. 10.3390/antibiotics11070868. 10.3390/antibiotics11070868 PMC9312233 35884122

[6] Maharjan P, Martinez D, Weil J, Suesuttajit N, Umberson C, Mullenix G, et al. Physiological growth trend of current meat broilers and dietary protein and energy management approaches for sustainable broiler production. Animal. 2021;15:100284. 10.1016/j.animal.2021.100284. 34246596 10.1016/j.animal.2021.100284

[7] Mishra B, Jha R. Oxidative stress in the poultry gut: potential challenges and interventions. Front Vet Sci. 2019;6:60. 10.3389/fvets.2019.00060. 30886854 10.3389/fvets.2019.00060 PMC6409315

[8] Surai PF, Kochish II, Fisinin VI, Kidd MT. Antioxidant defence systems and oxidative stress in poultry biology: an update. Antioxidants. 2019;8(7):235. 10.3390/antiox8070235. 31336672 10.3390/antiox8070235 PMC6680731

[9] Min Y, Niu Z, Sun T, Wang Z, Jiao P, Zi B, et al. Vitamin E and vitamin C supplementation improves antioxidant status and immune function in oxidative-stressed breeder roosters by upregulating expression of GSH-Px gene. Poult Sci. 2018;97(4):1238–44. 10.3382/ps/pex417. 29452404 10.3382/ps/pex417

[10] Wassie T, Lu Z, Duan X, Xie C, Gebeyew K, Yumei Z, et al. Dietary *Enteromorpha *polysaccharide enhances intestinal immune response, integrity, and caecal microbial activity of broiler chickens. Front Nutr. 2021:8:783819. 10.3389/fnut.2021.783819 10.3389/fnut.2021.783819 PMC8667661 34912840

[11] Su G, Wang L, Zhou X, Wu X, Chen D, Yu B, et al. Effects of essential oil on growth performance, digestibility, immunity, and intestinal health in broilers. Poult Sci. 2021;100(8):1012421–10. 10.1016/j.psj.2021.10124. 10.1016/j.psj.2021.101242 PMC8242051 34174571

[12] Suresh G, Das RK, Kaur Brar S, Rouissi T, Avalos Ramirez A, Chorfi Y, et al. Alternatives to antibiotics in poultry feed: molecular perspectives. Crit Rev Microbiol. 2018;44(3):318–35. 10.1080/1040841X.2017.1373062. 28891362 10.1080/1040841X.2017.1373062

[13] Obianwuna UE, Agbai Kalu N, Wang J, Zhang H, Qi G, Qiu K, et al. Recent trends on mitigative effect of probiotics on oxidative-stress-induced gut dysfunction in broilers under necrotic enteritis challenge: a review. Antioxidants. 2023;12(4):9111–34. 10.3390/antiox12040911. 10.3390/antiox12040911 PMC10136232 37107286

[14] Gessner D, Ringseis R, Eder K. Potential of plant polyphenols to combat oxidative stress and inflammatory processes in farm animals. J Anim Physiol Anim Nutr. 2017;101(4):605–28. 10.1111/jpn.12579. 10.1111/jpn.12579 27456323

[15] Gilani SMH, Rashid Z, Galani S, Ilyas S, Sahar S, Al-Ghanim K, et al. Growth performance, intestinal histomorphology, gut microflora and ghrelin gene expression analysis of broiler by supplementing natural growth promoters: a nutrigenomics approach. Saudi J Biol Sci. 2021;28(6):3438–47. 10.1016/j.sjbs.2021.03.008. 34121882 10.1016/j.sjbs.2021.03.008 PMC8176037

[16] Obianwuna UE, Oleforuh-Okoleh VU, Wang J, Zhang H-J, Qi GH, Qiu K, et al. Potential implications of natural antioxidants of plant origin on oxidative stability of chicken albumen during storage: a review. Antioxidants. 2022;11(4):6301–20. 10.3390/antiox11040630. 10.3390/antiox11040630 PMC9027279 35453315

[17] Obianwuna UE, Oleforuh-Okoleh VU, Wang J, Zhang H-J, Qi G-H, Qiu K, et al. Natural products of plants and animal origin improve albumen quality of chicken eggs. Front Nutr. 2022;9:8752701–19. 10.3389/fnut.2022.875270. 10.3389/fnut.2022.875270 PMC9226613 35757269

[18] Gungor E, Altop A, Erener G. Effect of raw and fermented grape seed on growth performance, antioxidant capacity, and cecal microflora in broiler chickens. Animal. 2021;15(4):100194. 10.1016/j.animal.2021.100194. 33640294 10.1016/j.animal.2021.100194

[19] Zhang L, Peng Q, Liu Y, Ma Q, Zhang J, Guo Y, et al. Effects of oregano essential oil as an antibiotic growth promoter alternative on growthf performance, antioxidant status, and intestinal health of broilers. Poult Sci. 2021;100(7):1011631–12. 10.1016/j.psj.2021.101163. 10.1016/j.psj.2021.101163 PMC8181178 34082177

[20] Ruan D, Fan Q, Fouad AM, Sun Y, Huang S, Wu A, et al. Effects of dietary oregano essential oil supplementation on growth performance, intestinal antioxidative capacity, immunity, and intestinal microbiota in yellow-feathered chickens. J Anim Sci. 2021;99(2):1–11. 10.1093/jas/skab033. 10.1093/jas/skab033 PMC7918158 33544855

[21] Islam Z, Sultan A, Khan S, Khan K, Jan AU, Aziz T, et al. Effects of an organic acids blend and coated essential oils on broiler growth performance, blood biochemical profile, gut health, and nutrient digestibility. Ital J Anim Sci. 2024;23(1):152–63. 10.1080/1828051X.2023.2297562.

[22] Dosu G, Obanla TO, Zhang S, Sang S, Adetunji AO, Fahrenholz AC, et al. Supplementation of ginger root extract into broiler chicken diet: effects on growth performance and immunocompetence. Poult Sci. 2023;102(10):102897. 10.1016/j.psj.2023.102897. 10.1016/j.psj.2023.102897 PMC10432838 37562125

[23] Zhang F, Yang J, Zhan Q, Shi H, Li Y, Li D, et al. Dietary oregano aqueous extract improves growth performance and intestinal health of broilers through modulating gut microbial compositions. J Anim Sci Biotechnol. 2023;14:77. 10.1186/s40104-023-00857-w. 10.1186/s40104-023-00857-w PMC10472629 37653529

[24] Wassie T, Cheng B, Zhou T, Gao L, Lu Z, Wang J, et al. *Enteromorpha *polysaccharide and yeast glycoprotein mixture improves growth, antioxidant activity, serum lipid profile and regulates lipid metabolism in broiler chickens. Poult Sci. 2022;101(10):102064. 10.1016/j.psj.2022.102064. 10.1016/j.psj.2022.102064 PMC9445391 36055019

[25] Wang Z, Yu H, Xie J, Cui H, Gao X. Effect of pectin oligosaccharides and zinc chelate on growth performance, zinc status, antioxidant ability, intestinal morphology and short-chain fatty acids in broilers. J Anim Physiol Nut. 2019;103(3):935–46. 10.1111/jpn.13076. 10.1111/jpn.13076 30801843

[26] Wang Q, Wang X, Xing T, Li J, Zhu X, Zhang L, et al. The combined impact of xylo-oligosaccharides and gamma-irradiated *Astragalus polysaccharides* on growth performance and intestinal mucosal barrier function of broilers. Poult Sci. 2021;100(3):100909. 10.1016/j.psj.2020.11.075. 10.1016/j.psj.2020.11.075 PMC7936216 33518329

[27] Park J, Kim I. Effects of a protease and essential oils on growth performance, blood cell profiles, nutrient retention, ileal microbiota, excreta gas emission, and breast meat quality in broiler chicks. Poult Sci. 2018;97(8):2854–60. 10.3382/ps/pey151. 29788490 10.3382/ps/pey151

[28] Michalczuk M, Holl E, Möddel A, Jóźwik A, Slósarz J, Bień D, et al. Phytogenic ingredients from hops and organic acids improve selected indices of welfare, health status markers, and bacteria composition in the caeca of broiler chickens. Animals (Basel). 2021;11(11):3249. 10.3390/ani11113249 10.3390/ani11113249 PMC8614400 34827980

[29] Tong Z, Lei F, Liu L, Wang F, Guo A. Effects of *Plotytarya strohilacea Sieb. et zuce* tannin on the growth performance, oxidation resistance, intestinal morphology and cecal microbial composition of broilers. Front Vet Sci. 2022;8:806105. 10.3389/fvets.2021.806105. 10.3389/fvets.2021.806105 PMC8766804 35071393

[30] Mengiste B, Zenebe T, Dires K, Lulekal E, Mekonnen A, Zegeye N, et al. Safety evaluation of *Eucalyptus globulus* essential oils through acute and sub-acute toxicity and skin irritation in mice and rats. Curr Chem Biol. 2020;14(3):187–95. 10.2174/2212796814999200818095036.

[31] Amer SA, Abdel-Wareth AA, Gouda A, Saleh GK, Nassar AH, Sherief WR, et al. Impact of dietary lavender essential oil on the growth and fatty Acid Profile of breast muscles, antioxidant activity, and inflammatory responses in broiler chickens. Antioxidants. 2022;11(9):1798. 10.3390/antiox11091798. 10.3390/antiox11091798 PMC9495784 36139872

[32] Jia Z, Dumont MJ, Orsat V. Encapsulation of phenolic compounds present in plants using protein matrices. Food Biosci. 2016;15:87–104. 10.1016/j.fbio.2016.05.007.

[33] Thuekeaw S, Angkanaporn K, Nuengjamnong C. Microencapsulated basil oil (*Ocimum basilicum *Linn.) enhances growth performance, intestinal morphology, and antioxidant capacity of broiler chickens in the tropics. Anim Bioscience. 2022;35(5):752–62. 10.5713/ab.21.0299. 10.5713/ab.21.0299 PMC9065782 34991219

[34] Febrianta H, Yunianto VD, Nurwantoro N, Bintoro VP. Dietary addition of microencapsulated turmeric in an amorphous matrix of maltodextrin on quality characteristics of broiler chicken. J Adv Vet Anim Res. 2022;9(2):221–9. 10.5455/javar.2022.i587. 35891663 10.5455/javar.2022.i587 PMC9298097

[35] Resta-Lenert S, Smitham J, Barrett KE. Epithelial dysfunction associated with the development of colitis in conventionally housed mdr1a^–/–^ mice. Am J Physiol Gastrointest Liver Physiol. 2005;289(1):G153–62. 10.1152/ajpgi.00395.2004. 15774938 10.1152/ajpgi.00395.2004

[36] Rao RK, Basuroy S, Rao VU, Karnaky KJ, Jr GA. Tyrosine phosphorylation and dissociation of occludin-ZO-1 and e-cadherin-beta-catenin complexes from the cytoskeleton by oxidative stress. Biochem J. 2002;368:471–81. 10.1042/bj20011804. 12169098 10.1042/BJ20011804 PMC1222996

[37] Zhong RZ, Zhou DW. Oxidative stress and role of natural plant derived antioxidants in animal reproduction. J Integr Agr. 2013;12(10):1826–38. 10.1016/S2095-3119(13)60412-8.

[38] Nawaz AH, Zhang L. Oxidative stress in broiler chicken and its consequences on meat quality. Int J Life Sci Res Archive. 2021;1(1):045–54. 10.53771/ijlsra.2021.1.1.0054.

[39] Chouhan S, Guleria S. Anti-inflammatory activity of medicinal plants: Present status and future perspectives. Bot Leads Drug Discovery. 2020;67–92. 10.1007/s11101-024-09955-7.

[40] Tylutka A, Walas L, Zembron-Lacny A. Level of IL-6, TNF, and IL-1β and age-related diseases: a systematic review and meta-analysis. Front Immunol. 2024;15:1330386. 10.3389/fimmu.2024.1330386. 10.3389/fimmu.2024.1330386 PMC10943692 38495887

[41] Güven G, Köseoğlu P, Lohmann E, Samancı B, Şahin E, Bilgiç B, et al. Peripheral expression of IL-6, TNF-α and TGF-β1 in Alzheimer’s Disease patients. Turkish J Immunol. 2024;12(1):128–34. 10.4274/tji.galenos.2024.76598.

[42] Albarrak SM. Antioxidant and immune responses of broiler chickens supplemented with *Rhazya stricta* extract in drinking water. Vet World. 2021;14(6):1437–49. 10.14202/vetworld.2021.1437-1449. 10.14202/vetworld.2021.1437-1449 PMC8304433 34316190

[43] Sierżant K, Piksa E, Konkol D, Lewandowska K, Asghar MU. Performance and antioxidant traits of broiler chickens fed with diets containing rapeseed or flaxseed oil and optimized quercetin. Sci Rep. 2023;13:14011. 10.1038/s41598-023-41282-3. 37640806 10.1038/s41598-023-41282-3 PMC10462632

[44] Zhao X, Du B, Wan M, Li J, Qin S, Nian F, et al. Analysis of the antioxidant activity of toons sinensis extract and their biological effects on broilers. Front Vet Sci. 2024;10:1337291. 10.3389/fvets.2023.1337291. 10.3389/fvets.2023.1337291 PMC10800727 38260193

[45] Surai PF, Earle-Payne K. Antioxidant defences and redox homeostasis in animals. Antioxidants. 2022;11(5):1012. 10.3390/antiox11051012. 10.3390/antiox11051012 PMC9137460 35624875

[46] Song B, Tang D, Yan S, Fan H, Li G, Shahid, et al. Effects of age on immune function in broiler chickens. J Anim Sci Biotechnol. 2021;12:42. 10.1186/s40104-021-00559-1. 10.1186/s40104-021-00559-1 PMC7971956 33731181

[47] Wlaźlak S, Pietrzak E, Biesek J, Dunislawska A. Modulation of the immune system of chickens a key factor in maintaining poultry production—a review. Poult Sci. 2023;102(8):102785. 10.1016/j.psj.2023.102785. 37267642 10.1016/j.psj.2023.102785 PMC10244701

[48] Morgan PM. Immune response in mammals and chickens. In: Zhang XY, Vieira-Pires RS, Morgan PM, Schade R, editors. IgY-Technology: production and application of egg yolk antibodies. Cham: Springer; 2021. p. 31–47. 10.1007/978-3-030-72688-1_3.

[49] Mantis NJ, Rol N, Corthésy B. Secretory IgA’s complex roles in immunity and mucosal homeostasis in the gut. Mucosal Immunol. 2011;4(6):603–11. 10.1038/mi.2011.41. 10.1038/mi.2011.41 PMC3774538 21975936

[50] Kulkarni RR, Gaghan C, Mohammed J, Sharif S, Taha-Abdelaziz K. Cellular immune responses in lymphoid tissues of broiler chickens experimentally infected with necrotic enteritis–producing *Clostridium perfringens* strains. Avian Dis. 2023;67(2):186–96. 10.1637/aviandiseases-D-23-00012. 37556298 10.1637/aviandiseases-D-23-00012

[51] Koarada S, Wu Y, Olshansky G, Ridgway WM. Increased nonobese diabetic Th1:Th2 (IFN-γ:IL-4) ratio is CD4^+^ T cell intrinsic and independent of APC genetic background. J Immunol. 2002;169(11):6580–7. 10.4049/jimmunol.169.11.6580. 12444170 10.4049/jimmunol.169.11.6580

[52] Alagawany M, Elnesr SS, Farag MR, Abd El-Hack ME, Barkat RA, Gabr AA, et al. Potential role of important nutraceuticals in poultry performance and health-A comprehensive review. Res Vet Sci. 2021;137:9–29. 10.1016/j.rvsc.2021.04.009. 33915364 10.1016/j.rvsc.2021.04.009

[53] Ismail IE, Alagawany M, Taha AE, Puvača N, Laudadio V, Tufarelli V. Effect of dietary supplementation of garlic powder and phenylacetic acid on productive performance, blood haematology, immunity and antioxidant status of broiler chickens. Anim Biosci. 2021;34(3):363–70. 10.5713/ajas.20.0140. 10.5713/ajas.20.0140 PMC7961197 32777893

[54] Yang G, Bibi S, Du M, Suzuki T, Zhu MJ. Regulation of the intestinal tight junction by natural polyphenols: a mechanistic perspective. Crit Rev Food Sci. 2017;57(18):3830–9. 10.1080/10408398.2016.1152230. 10.1080/10408398.2016.1152230 27008212

[55] Pandey U, Aich P. Postnatal intestinal mucosa and gut microbial composition develop hand in hand: a mouse study. Biomed J. 2023;46(2):100519. 10.1016/j.bj.2022.03.004. 10.1016/j.bj.2022.03.004 PMC10267966 35306225

[56] Qiao Y, Liu C, Guo Y, Zhang W, Guo W, Oleksandr K, et al. Polysaccharides derived from *Astragalus membranaceus* and *Glycyrrhiza uralensis* improve growth performance of broilers by enhancing intestinal health and modulating gut microbiota. Poult Sci. 2022;101(7):101905. 10.1016/j.psj.2022.101905. 10.1016/j.psj.2022.101905 PMC9117935 35576745

[57] Lehrer RI, Bevins CL, Ganz T. Defensins and other antimicrobial peptides and proteins. In: Mestecky J, Lamm ME, McGhee JR, Bienenstock J, Mayer L, Strober W, editors. Mucosal Immunology. 3rd ed. Academic Press. 2005. p. 95–110. 10.1016/B978-012491543-5/50010-3.

[58] Kopp ZA, Jain U, Van Limbergen J, Stadnyk AW. Do antimicrobial peptides and complement collaborate in the intestinal mucosa? Front Immunol. 2015;6:17. 10.3389/fimmu.2015.00017. 10.3389/fimmu.2015.00017 PMC4311685 25688244

[59] Nieto N, Torres M, Fernandez M, Giron M, Rios A, Suarez M, et al. Experimental ulcerative colitis impairs antioxidant defense system in rat intestine. Digest Dis Sci. 2000;45:1820–7. 10.1023/a:1005565708038. 10.1023/a:1005565708038 11052326

[60] Tan Z, Ou Y, Cai W, Zheng Y, Li H, Mao Y, et al. Advances in the clinical application of histamine and diamine oxidase (DAO) activity: a review. Catalysts. 2022;13(1):48. 10.3390/catal13010048.

[61] Cucca V, Ramirez GA, Pignatti P, Asperti C, Russo M, Della-Torre E, et al. Basal serum diamine oxidase levels as a biomarker of histamine intolerance: a retrospective cohort study. Nutrients. 2022;14(7):1513. 10.3390/nu14071513. 10.3390/nu14071513 PMC9003468 35406126

[62] Xu Q, Zhao J, Jian H, Ye J, Gong M, Zou X, et al. Linoleic acid ameliorates intestinal mucosal barrier injury in early weaned pigeon squabs (*Columba livia*). J Anim Sci. 2023;101:skad125. 10.1093/jas/skad125. 10.1093/jas/skad125 PMC10195202 37186172

[63] Kirpich IA, Feng W, Wang Y, Liu Y, Beier JI, Arteel GE, et al. Ethanol and dietary unsaturated fat (corn oil/linoleic acid enriched) cause intestinal inflammation and impaired intestinal barrier defense in mice chronically fed alcohol. Alcohol. 2013;47(3):257–64. 10.1016/j.alcohol.2013.01.005. 10.1016/j.alcohol.2013.01.005 PMC3617059 23453163

[64] Wu Y, Shao Y, Song B, Zhen W, Wang Z, Guo Y, et al. Effects of *Bacillus coagulans* supplementation on the growth performance and gut health of broiler chickens with *Clostridium perfringens*-induced necrotic enteritis. J Anim Sci Biotechnol. 2018;9:9. 10.1186/s40104-017-0220-2. 10.1186/s40104-017-0220-2 PMC5784659 29416856

[65] Circu ML, Aw TY. Intestinal redox biology and oxidative stress. Semin Cell Dev Biol. 2012;23(7):729–37. 10.1016/j.semcdb.2012.03.014. 22484611 10.1016/j.semcdb.2012.03.014 PMC3396776

[66] Saleh AA, Ebeid TA, Abudabos AM. Effect of dietary phytogenics (herbal mixture) supplementation on growth performance, nutrient utilization, antioxidative properties, and immune response in broilers. Environ Sci Pollut R. 2018;25:14606–13. 10.1007/s11356-018-1685-z. 10.1007/s11356-018-1685-z 29532373

[67] Zihni C, Mills C, Matter K, Balda MS. Tight junctions: from simple barriers to multifunctional molecular gates. Nat Rev Mol Cell Biol. 2016;17(9):564–80. 10.1038/nrm.2016.80. 27353478 10.1038/nrm.2016.80

[68] He E, Quan W, Luo J, Liu C, Zheng W, Shen Q. Absorption and transport mechanism of red meat-derived N-glycolylneuraminic acid and its damage to intestinal barrier function through the NF-κB signaling pathway. Toxins. 2023;15:132. 10.3390/toxins1502013. 36828446 10.3390/toxins15020132 PMC9966629

[69] Gonzalez-Avila G, Sommer B, Mendoza-Posada DA, Ramos C, Garcia-Hernandez AA, Falfan-Valencia R. Matrix metalloproteinases participation in the metastatic process and their diagnostic and therapeutic applications in cancer. Crit Rev Oncol/Hematol. 2019;137:57–83. 10.1016/j.critrevonc.2019.02.010. 31014516 10.1016/j.critrevonc.2019.02.010

[70] Dubreuil JD. Enterotoxigenic *Escherichia coli* targeting intestinal epithelial tight junctions: an effective way to alter the barrier integrity. Microb Pathog. 2017;113:129–34. 10.1016/j.micpath.2017.10.037. 29079214 10.1016/j.micpath.2017.10.037

[71] Xiao D, Wang Z, Dai X, Hu Y, Zhong M, Xiong L, et al. Effects of *Bacillus methylotrophicus* SY200 supplementation on growth performance, antioxidant status, intestinal morphology, and immune function in broiler chickens. Probiotics Antimicro. 2023;15(4):925–40. 10.1007/s12602-022-09924-6. 10.1007/s12602-022-09924-6 35150396

[72] Long L, Zhang H, Wang F, Yin Y, Yang L, Chen J. Research note: effects of polysaccharide-enriched *Acanthopanax senticosus* extract on growth performance, immune function, antioxidation, and ileal microbial populations in broiler chickens. Poult Sci. 2021;100(4):101028. 10.1016/j.psj.2021.101028. 33647719 10.1016/j.psj.2021.101028 PMC7921867

[73] Fernandez-Alarcon M, Trottier N, Steibel J, Lunedo R, Campos D, Santana A, et al. Interference of age and supplementation of direct-fed microbial and essential oil in the activity of digestive enzymes and expression of genes related to transport and digestion of carbohydrates and proteins in the small intestine of broilers. Poult Sci. 2017;96(8):2920–30. 10.3382/ps/pex039. 28339792 10.3382/ps/pex039

[74] Celi P, Verlhac V, Calvo EP, Schmeisser J, Kluenter AM. Biomarkers of gastrointestinal functionality in animal nutrition and health. Anim Feed Sci Tech. 2019;250:9–31. 10.1016/j.anifeedsci.2018.07.012.

[75] Al-Baadani HH, Al-Mufarrej SI, Azzam MM, Alharthi AS, Al-Garadi MA, Al-Gabri NA, et al. Evaluation of gum Arabic* (Acacia senegal)* as a natural prebiotic to improve growth performance and health status of broiler chickens. Trop Anim Health Prod. 2022;54(4):244. 10.1007/s11250-022-03245-0. 10.1007/s11250-022-03245-0 35913613

[76] Shaona L, Bin Z, Decai X, Zhiyong Z, Yajie C, Qian S. Effects of *Bacillus velezensis* on growth performance, fecal microbiota and metabolites in pigs. Chin J Anim Nutr. 2020;33:5622–35.

[77] Quan J, Cai G, Yang M, Zeng Z, Ding R, Wang X, et al. Exploring the fecal microbial composition and metagenomic functional capacities associated with feed efficiency in commercial DLY pigs. Front Microbiol. 2019;10:521–12. 10.3389/fmicb.2019.00052. 10.3389/fmicb.2019.00052 PMC6361760 30761104

[78] Rizzatti G, Lopetuso LR, Gibiino G, Binda C, Gasbarrini A. Proteobacteria: a common factor in human diseases. BioMed Res Int. 2017;2017(1):9351507. 10.1155/2017/9351507. 10.1155/2017/9351507 PMC5688358 29230419

[79] Nikonov IN, Il’ina LA, Kochish II, Romanov MN, Podobed LI, Laptev GY, et al. Changing the intestinal microbiota of chickens in ontogenesis. Ukrainian J Ecol. 2017;7(4):492–9. 10.15421/2017_150.

[80] Rychlik I. Composition and function of Chicken gut microbiota. Animals (Basel). 2020;10(1):103. 10.3390/ani10010103. 10.3390/ani10010103 PMC7022619 31936291

[81] Chen Y, Wang J, Yu L, Xu T, Zhu N. Microbiota and metabolome responses in the cecum and serum of broiler chickens fed with plant essential oils or virginiamycin. Sci Rep. 2020;10:5382. 10.1038/s41598-020-60135-x. 32214106 10.1038/s41598-020-60135-x PMC7096418

[82] Stevenson DM, Weimer PJ. Dominance of *Prevotella* and low abundance of classical ruminal bacterial species in the bovine rumen revealed by relative quantification real-time PCR. Appl Microbiol Biot. 2007;75:165–74. 10.1007/s00253-006-0802-y. 10.1007/s00253-006-0802-y 17235560

[83] Zeng Q, Li D, He Y, Li Y, Yang Z, Zhao X, et al. Discrepant gut microbiota markers for the classification of obesity-related metabolic abnormalities. Sci Rep. 2019;9:13424. 10.1038/s41598-019-49462-w. 10.1038/s41598-019-49462-w PMC6748942 31530820

[84] Chang WY, Yu YH. Effect of *Bacillus* species–fermented products and essential oils on growth performance, gut morphology, cecal short-chain fatty acid levels, and microbiota community in broilers. Poult Sci. 2022;101(8):101970. 10.1016/j.psj.2022.101970. 10.1016/j.psj.2022.101970 PMC9241036 35760005

[85] Zhao J, Zhang X, Liu H, Brown MA, Qiao S. Dietary protein and gut microbiota composition and function. Curr Protein Pept Sc. 2019;20(2):145–54. 10.2174/1389203719666180514145437. 29756574 10.2174/1389203719666180514145437

[86] LeBlanc JG, Milani C, De Giori GS, Sesma F, Van Sinderen D, Ventura M. Bacteria as vitamin suppliers to their host: a gut microbiota perspective. Curr Opin Biotech. 2013;24(2):160–8. 10.1016/j.copbio.2012.08.005. 22940212 10.1016/j.copbio.2012.08.005

[87] Nogal A, Valdes AM, Menni C. The role of short-chain fatty acids in the interplay between gut microbiota and diet in cardio-metabolic health. Gut Microbes. 2021;13(1):1897212. 10.1080/19490976.2021.1897212. 33764858 10.1080/19490976.2021.1897212 PMC8007165

[88] Rossi O, Van Berkel LA, Chain F, Tanweer Khan M, Taverne N, Sokol H, et al. *Faecalibacterium prausnitzii* A2-165 has a high capacity to induce IL-10 in human and murine dendritic cells and modulates T cell responses. Sci Rep. 2016;6:18507. 10.1038/srep18507. 10.1038/srep18507 PMC4698756 26725514

[89] Maioli TU, Borras-Nogues E, Torres L, Barbosa SC, Martins VD, Langella P, et al. Possible benefits of *Faecalibacterium prausnitzii* for obesity-associated gut disorders. Front Pharmacol. 2021;12:740636. 10.3389/fphar.2021.740636. 10.3389/fphar.2021.740636 PMC8677946 34925006

[90] Patel R, DuPont HL. New approaches for bacteriotherapy: prebiotics, new-generation probiotics, and synbiotics. Clin Infect Dis. 2015;60(Suppl 2):S108–121. 10.1093/cid/civ177. 25922396 10.1093/cid/civ177 PMC4490231

[91] Lai HC, Lin TL, Chen TW, Kuo YL, Chang CJ, Wu TR, et al. Gut microbiota modulates COPD pathogenesis: role of anti-inflammatory *Parabacteroides goldsteinii* lipopolysaccharide. Gut. 2022;71(2):309–21. 10.1136/gutjnl-2020-322599. 33687943 10.1136/gutjnl-2020-322599

[92] Koh GY, Kane AV, Wu X, Crott JW. *Parabacteroides distasonis* attenuates tumorigenesis, modulates inflammatory markers and promotes intestinal barrier integrity in azoxymethane-treated A/J mice. Carcinogenesis. 2020;41(7):909–17. 10.1093/cdn/nzz030.OR04-02-19. 32115637 10.1093/carcin/bgaa018

[93] Kim M, Qie Y, Park J, Kim CH. Gut microbial metabolites fuel host antibody responses. Cell Host Microbe. 2016;20(2):202–14. 10.1016/j.chom.2016.07.001. 27476413 10.1016/j.chom.2016.07.001 PMC4982788

[94] Abd El-Hack ME, El-Saadony MT, Salem HM, El-Tahan AM, Soliman MM, Youssef GBA, et al. Alternatives to antibiotics for organic poultry production: types, modes of action and impacts on bird’s health and production. Poult Sci. 2022;101(4):101696. 10.1016/j.psj.2022.101696. 10.1016/j.psj.2022.101696 PMC8844281 35150942

[95] Ayalew H, Zhang H, Wang J, Wu S, Qiu K, Qi G, et al. Potential feed additives as antibiotic alternatives in broiler production. Front Vet Sci. 2022;9:916473. 10.3389/fvets.2022.916473 10.3389/fvets.2022.916473 PMC9247512 35782570

[96] Ibrahim D, Sewid AH, Arisha AH, Abd El-Fattah AH, Abdelaziz AM, Al-Jabr OA, et al. Influence of *Glycyrrhiza glabra* extract on growth, gene expression of gut integrity, and *Campylobacter jejuni* colonization in broiler chickens. Front Vet Sci. 2020;7:612063. 10.3389/fvets.2020.612063. 10.3389/fvets.2020.612063 PMC7782238 33415133

[97] Nazarizadeh H, Mohammad Hosseini S, Pourreza J. Effect of plant extracts derived from thyme and chamomile on the growth performance, gut morphology and immune system of broilers fed aflatoxin B1 and ochratoxin A contaminated diets. Ital J Anim Sci. 2019;18(1):1073–81. 10.1080/1828051X.2019.1615851.

[98] Galovičová L, Borotová P, Valková V, Vukovic NL, Vukic M, Štefániková J, et al. *Thymus vulgaris* essential oil and its biological activity. Plants. 2021;10(9):1959. 10.3390/plants10091959. 10.3390/plants10091959 PMC8467294 34579491

[99] Xie P, Deng Y, Huang L, Zhang C. Effect of olive leaf (*Olea europaea *L.) extract addition to broiler diets on the growth performance, breast meat quality, antioxidant capacity and caecal bacterial populations. Ital J Anim Sci. 2022;21(1):1246–58. 10.1080/1828051X.2022.2105265.

[100] Ghiasvand A, Khatibjoo A, Mohammadi Y, Akbari Gharaei M, Shirzadi H. Effect of fennel essential oil on performance, serum biochemistry, immunity, ileum morphology and microbial population, and meat quality of broiler chickens fed corn or wheat-based diet. Br Poult Sci. 2021;62(4):562–72. 10.1080/00071668.2021.1883551. 33530744 10.1080/00071668.2021.1883551

[101] Stefanello C, Moreira B, Gräf W, Robalo S, Costa S, Vieira I, et al. Effects of a proprietary blend of *Quillaja* and *Yucca* on growth performance, nutrient digestibility, and intestinal measurements of broilers. J Appl Poult Res. 2022;31(2):100251. 10.1016/j.japr.2022.100251.

[102] Rostampour B, Chamani M, Seidavi A, Zarei A, Karimi N. The effect of *Froriepia subpinnata* on the performance, carcass characteristics, blood parameters, immune system, microbial population, intestinal morphology, and breast meat fatty acid content of broiler chickens. Trop Anim Health Prod. 2024;56:43. 10.1007/s11250-024-03887-2. 10.1007/s11250-024-03887-2 38217627

[103] Alharthi AS, Alruwaili NW, Al-Baadani HH, Al-Garadi MA, Shamlan G, Alhidary IA. Investigating the effect of *Pulicaria jaubertii* as a natural feed additive on the growth performance, blood biochemistry, immunological response, and cecal microbiota of broiler chickens. Animals. 2023;13(6):1116. 10.3390/ani13061116. 10.3390/ani13061116 PMC10044572 36978656

[104] Shirani V, Jazi V, Toghyani M, Ashayerizadeh A, Sharifi F, Barekatain R. *Pulicaria gnaphalodes* powder in broiler diets: consequences for performance, gut health, antioxidant enzyme activity, and fatty acid profile. Poult Sci. 2019;98(6):2577–87. 10.3382/ps/pez010. 30690512 10.3382/ps/pez010

[105] Gurram S, Chinni Preetam V, Vijaya Lakshmi K, Raju M, Venkateswarlu M, Bora S. Synergistic effect of probiotic, chicory root powder and coriander seed powder on growth performance, antioxidant activity and gut health of broiler chickens. PLoS ONE. 2022;17(6):e0270231. 10.1371/journal.pone.0270231. 10.1371/journal.pone.0270231 PMC9236266 35759473

[106] Shen G, Luo Y, Yao Y, Meng G, Zhang Y, Wang Y, et al. The discovery of a key prenyltransferase gene assisted by a chromosome-level *Epimedium pubescens* genome. Front Plant Sci. 2022;13:1034943. 10.3389/fpls.2022.1034943. 10.3389/fpls.2022.1034943 PMC9702526 36452098

[107] Xia N, Pautz A, Wollscheid U, Reifenberg G, Förstermann U, Li H. Artichoke, cynarin and cyanidin downregulate the expression of inducible nitric oxide synthase in human coronary smooth muscle cells. Molecules. 2014;19(3):3654–68. 10.3390/molecules19033654. 24662080 10.3390/molecules19033654 PMC6271736

[108] Guo S, Hu J, Ai S, Li L, Ding B, Zhao D, et al. Effects of *pueraria* extract and curcumin on growth performance, antioxidant status and intestinal integrity of broiler chickens. Animals. 2023;13(8):1276. 10.3390/ani13081276. 10.3390/ani13081276 PMC10135329 37106839

[109] Montoya-García CO, García-Mateos R, Becerra-Martínez E, Toledo-Aguilar R, Volke-Haller VH, Magdaleno-Villar JJ. Bioactive compounds of purslane (*Portulaca oleracea *L.) according to the production system: A review. Sci Hortic-Amsterdam. 2023;308:111584. 10.1016/j.scienta.2022.111584.

[110] Holleran G, Scaldaferri F, Gasbarrini A, Currò D. Herbal medicinal products for inflammatory bowel disease: a focus on those assessed in double-blind randomised controlled trials. Phytother Res. 2020;34(1):77–93. 10.1002/ptr.6517. 31701598 10.1002/ptr.6517

[111] Hu QF, Wu F, Zhu YN, Liu L, Liu MX, Cai BB, et al. Three new anti-rotavirus quinoline alkaloids from the whole plant of *Thalictrum glandulosissimum*. Heterocyles. 2021;102(8):1588–94. 10.3987/COM-21-14484.

[112] Wu T, Jin T. Prevention and treatment of digestive tract issues induced by postoperative adjuvant chemotherapy for pancreatic cancer using Terra Flava Usta. Am J Transl Res. 2021;13(4):3182–9. PMC8129296 34017487

[113] Junren C, Xiaofang X, Mengting L, Qiuyun X, Gangmin L, Huiqiong Z, et al. Pharmacological activities and mechanisms of action of *Pogostemon cablin *Benth: a review. Chin Med. 2021;16(5):1–20. 10.1186/s13020-020-00413-y. 10.1186/s13020-020-00413-y PMC7791836 33413544

[114] Rockenbach II, Gonzaga LV, Rizelio VM, Gonçalves AESS, Genovese MI, Fett R. Phenolic compounds and antioxidant activity of seed and skin extracts of red grape (*Vitis vinifera* and *Vitis labrusca*) pomace from Brazilian winemaking. Food Res Int. 2011;44(4):897–901. 10.1016/j.foodres.2011.01.049.

[115] Meng T, Xiao D, Muhammed A, Deng J, Chen L, He J. Anti-inflammatory action and mechanisms of resveratrol. Molecules. 2021;26(1):229. 10.3390/molecules26010229. 10.3390/molecules26010229 PMC7796143 33466247

[116] Xing C, Wang Y, Dai X, Yang F, Luo J, Liu P, et al. The protective effects of resveratrol on antioxidant function and the mRNA expression of inflammatory cytokines in the ovaries of hens with fatty liver hemorrhagic syndrome. Poult Sci. 2020;99(2):1019–27. 10.1016/j.psj.2019.10.009. 32036959 10.1016/j.psj.2019.10.009 PMC7587695

[117] Liu X, Wang Y, Wu D, Li S, Wang C, Han Z, et al. Magnolol prevents acute alcoholic liver damage by activating PI3K/Nrf2/PPARγ and inhibiting NLRP3 signaling pathway. Front Pharmacol. 2019;10:1459. 10.3389/fphar.2019.01459. 10.3389/fphar.2019.01459 PMC6915046 31920652

[118] Chen H, Fu W, Chen H, You S, Liu X, Yang Y, et al. Magnolol attenuates the inflammation and enhances phagocytosis through the activation of MAPK, NF-κB signal pathways in vitro and in vivo. Mol Immunol. 2019;105:96–106. 10.1016/j.molimm.2018.11.008. 10.1016/j.molimm.2018.11.008 30500626

[119] Kuo NC, Huang SY, Yang CY, Shen HH, Lee YM. Involvement of HO-1 and autophagy in the protective effect of magnolol in hepatic steatosis-induced NLRP3 inflammasome activation in vivo and in vitro. Antioxidants. 2020;9(10):924. 10.3390/antiox9100924. 10.3390/antiox9100924 PMC7600324 32992548

[120] Chen J, Kang B, Zhao Y, Yao K, Fu C. Effects of natural dietary supplementation with *Macleaya cordata* extract containing sanguinarine on growth performance and gut health of early-weaned piglets. J Anim Physiol Anim Nutr. 2018;102(6):1666–74. 10.1111/jpn.12976. 10.1111/jpn.12976 30129225

[121] Shiffman ME, Soo RM, Dennis PG, Morrison M, Tyson GW, Hugenholtz P. Gene and genome-centric analyses of koala and wombat fecal microbiomes point to metabolic specialization for Eucalyptus digestion. PeerJ. 2017;5:e4075. 10.7717/peerj.4075. 29177117 10.7717/peerj.4075 PMC5697889

[122] Youssef IM, Männer K, Zentek J. Effect of essential oils or saponins alone or in combination on productive performance, intestinal morphology and digestive enzymes’ activity of broiler chickens. J Anim Physiol Anim Nutr (Berl). 2021;105(1):99–107. 10.1111/jpn.13431. 32755039 10.1111/jpn.13431

[123] Jamroz D, Wertelecki T, Houszka M, Kamel C. Influence of diet type on the inclusion of plant origin active substances on morphological and histochemical characteristics of the stomach and jejunum walls in chicken. J Anim Physiol Anim Nutr (Berl). 2006;90(5–6):255–68. 10.1111/j.1439-0396.2005.00603.x. 16684147 10.1111/j.1439-0396.2005.00603.x

[124] Khudhayer Oglah M, Fakri Mustafa Y. Curcumin analogs: synthesis and biological activities. Med Chem Res. 2020;29:479–86. 10.1007/s00044-019-02497-0.

[125] Batiha GES, Alkazmi LM, Wasef LG, Beshbishy AM, Nadwa EH, Rashwan EK. *Syzygium aromaticum* L. (Myrtaceae): traditional uses, bioactive chemical constituents, pharmacological and toxicological activities. Biomolecules. 2020;10(2):202. 10.3390/biom10020202. 10.3390/biom10020202 PMC7072209 32019140

[126] Shao D, Li J, Li J, Tang R, Liu L, Shi J, et al. Inhibition of gallic acid on the growth and biofilm formation of *Escherichia coli* and *Streptococcus mutans*. J Food Sci. 2015;80(6):M1299–305. 10.1111/1750-3841.12902. 25974286 10.1111/1750-3841.12902

[127] Diguță C, Cornea C, Ioniță L, Brîndușe E, Farcaș N, Bobit D, et al. Studies on antimicrobial activity of *Inula helenium *L Romanian cultivar. Rom Biotech Lett. 2014;19(5):9699–704.

[128] Kenny CR, Stojakowska A, Furey A, Lucey B. From monographs to chromatograms: the antimicrobial potential of *Inula helenium* L. (Elecampane) naturalised in Ireland. Molecules. 2022;27(4):1406. 10.3390/molecules27041406. 35209195 10.3390/molecules27041406 PMC8874828

[129] Zou Q, Meng W, Li C, Wang T, Li D. Feeding broilers with wheat germ, hops and grape seed extract mixture improves growth performance. Front Physiol. 2023;14:1144997. 10.3389/fphys.2023.1144997. 10.3389/fphys.2023.1144997 PMC10086265 37057186

[130] Salavati ME, Rezaeipour V, Abdullahpour R, Mousavi N. Effects of graded inclusion of bioactive peptides derived from sesame meal on the growth performance, internal organs, gut microbiota and intestinal morphology of broiler chickens. Int J Pept Res Ther. 2020;26:1541–8. 10.1007/s10989-019-09947-8.

[131] Tiwari R, Latheef SK, Ahmed I, Iqbal H, Bule MH, Dhama K, et al. Herbal immunomodulators-a remedial panacea for designing and developing effective drugs and medicines: current scenario and future prospects. Curr Drug Metab. 2018;19(3):264–301. 10.2174/1389200219666180129125436. 29380694 10.2174/1389200219666180129125436

[132] MacDonald TT, Monteleone G. Immunity, inflammation, and allergy in the gut. Science. 2005;307(5717):1920–5. 10.1126/science.1106442. 15790845 10.1126/science.1106442

[133] Hesabi Nameghi A, Edalatian O, Bakhshalinejad R. Effects of a blend of thyme, peppermint and eucalyptus essential oils on growth performance, serum lipid and hepatic enzyme indices, immune response and ileal morphology and microflora in broilers. J Anim Physiol Anim Nutr. 2019;103(5):1388–98. 10.1111/jpn.13122. 10.1111/jpn.13122 31106919

[134] Burt S. Essential oils: their antibacterial properties and potential applications in foods—a review. Int J Food Microbiol. 2004;94(3):223–53. 10.1016/j.ijfoodmicro.2004.03.022. 15246235 10.1016/j.ijfoodmicro.2004.03.022

[135] Mączka W, Twardawska M, Grabarczyk M, Wińska K. Carvacrol-A natural phenolic compound with antimicrobial properties. Antibiotics (Basel). 2023;12(5):824. 10.3390/antibiotics12050824. 10.3390/antibiotics12050824 PMC10215463 37237727

[136] Farag MR, Alagawany M, Tufarelli V. In vitro antioxidant activities of resveratrol, cinnamaldehyde and their synergistic effect against cyadox-induced cytotoxicity in rabbit erythrocytes. Drug Chem Toxicol. 2017;40(2):196–205. 10.1080/01480545.2016.1193866. 10.1080/01480545.2016.1193866 27314888

[137] Nazzaro F, Fratianni F, Coppola R, De Feo V. Essential oils and antifungal activity. Pharmaceuticals. 2017;10(4):86. 10.3390/ph10040086. 29099084 10.3390/ph10040086 PMC5748643

[138] Mohebodini H, Jazi V, Ashayerizadeh A, Toghyani M, Tellez-Isaias G. Productive parameters, cecal microflora, nutrient digestibility, antioxidant status, and thigh muscle fatty acid profile in broiler chickens fed with *Eucalyptus globulus* essential oil. Poult Sci. 2021;100(3):100922. 10.1016/j.psj.2020.12.020. 10.1016/j.psj.2020.12.020 PMC7936223 33652520

[139] Long S, Xu Y, Wang C, Li C, Liu D, Piao X. Effects of dietary supplementation with a combination of plant oils on performance, meat quality and fatty acid deposition of broilers. Asian Austral J Anim. 2018;31(11):1773. 10.5713/ajas.18.0056. 10.5713/ajas.18.0056 PMC6212761 29642679

[140] Xie Q, Xie K, Yi J, Song Z, Zhang H, He X. The effects of magnolol supplementation on growth performance, meat quality, oxidative capacity, and intestinal microbiota in broilers. Poult Sci. 2022;101(4):101722. 10.1016/j.psj.2022.101722. 35196587 10.1016/j.psj.2022.101722 PMC8866717

[141] Chamorro S, Romero C, Brenes A, Sánchez-Patán F, Bartolomé B, Viveros A, et al. Impact of a sustained consumption of grape extract on digestion, gut microbial metabolism and intestinal barrier in broiler chickens. Food Funct. 2019;10(3):1444–54. 10.1039/C8FO02465K. 30768097 10.1039/c8fo02465k

[142] Xie T, Liu W, Chen Y, Zhou Y. An evaluation of graded levels of beta-sitosterol supplementation on growth performance, antioxidant status, and intestinal permeability-related parameters and morphology in broiler chickens at an early age. Poult Sci. 2022;101(11):102108. 10.1016/j.psj.2022.102108. 10.1016/j.psj.2022.102108 PMC9472065 36099659

[143] Du J, Wang Y, Wang R, Ji X, Zhang J, Guo T, et al. Effect of ferulic acid on growth, digestibility, digestive enzyme activity, immunity and antioxidant status of broilers. S Afr J Anim Sci. 2022;52(3):280–90. 10.4314/sajas.v52i3.4.

[144] Abo-Samaha MI, Alghamdi YS, El-Shobokshy SA, Albogami S, El-Maksoud EMA, Farrag F, et al. Licorice extract supplementation affects antioxidant activity, growth-related genes, lipid metabolism, and Immune markers in broiler chickens. Life. 2022;12(6):9141–21. 10.3390/life12060914. 10.3390/life12060914 PMC9225592 35743945

[145] Herrero-Encinas J, Huerta A, Blanch M, Pastor JJ, Morais S, Menoyo D. Impact of dietary supplementation of spice extracts on growth performance, nutrient digestibility and antioxidant response in broiler chickens. Animals. 2023;13(2):250. 10.3390/ani13020250. 36670790 10.3390/ani13020250 PMC9854518

[146] Long S, He T, Wu D, Yang M, Piao X. *Forsythia suspensa* extract enhances performance via the improvement of nutrient digestibility, antioxidant status, anti-inflammatory function, and gut morphology in broilers. Poult Sci. 2020;99(9):4217–26. 10.1016/j.psj.2020.05.011. 32867965 10.1016/j.psj.2020.05.011 PMC7598019

[147] Liu S, Xiao G, Wang Q, Tian J, Feng X, Zhang Q, et al. Effects of dietary *Astragalus membranaceus* and *Codonopsis pilosula* extracts on growth performance, antioxidant capacity, immune status, and intestinal health in broilers. Front Vet Sci. 2023;10:1302801. 10.3389/fvets.2023.1302801. 10.3389/fvets.2023.1302801 PMC10748503 38144468

[148] Dai Z, Wang H, Liu J, Zhang H, Li Q, Yu X, et al. Comparison of the effects of *Yucca saponin*, *Yucca schidigera*, and *Quillaja saponaria* on growth performance, immunity, antioxidant capability, and intestinal flora in broilers. Animals. 2023;13(9):1447. 10.3390/ani13091447. 10.3390/ani13091447 PMC10177514 37174484

[149] Liu M, Chen R, Wang T, Ding Y, Zhang Y, Huang G, et al. Dietary Chinese herbal mixture supplementation improves production performance by regulating reproductive hormones, antioxidant capacity, immunity, and intestinal health of broiler breeders. Poult Sci. 2024;103(1):103201. 10.1016/j.psj.2023.103201. 10.1016/j.psj.2023.103201 PMC10692728 37980727

[150] Zhao G, Niu Y, Wang H, Qin S, Zhang R, Wu Y, et al. Effects of three different plant-derived polysaccharides on growth performance, immunity, antioxidant function, and cecal microbiota of broilers. J Sci Food Agr. 2024;104(2):1020–9. 10.1002/jsfa.12988. 37718500 10.1002/jsfa.12988

[151] Saied A, Attia A, El-Kholy M, Reda F, Nagar A. Effect of cinnamon oil supplementation into broiler chicken diets on growth, carcass traits, haemato-biochemical parameters, immune function, antioxidant status and caecal microbial count. J Anim Feed Sci. 2022;31:21–33. 10.22358/jafs/146921/2022.

[152] Ding Y, Hu Y, Yao X, He Y, Chen J, Wu J, et al. Dietary essential oils improve the growth performance, antioxidant properties and intestinal permeability by inhibiting bacterial proliferation, and altering the gut microbiota of yellow-feather broilers. Poult Sci. 2022;101(11):102087. 10.1016/j.psj.2022.102087. 10.1016/j.psj.2022.102087 PMC9472070 36095866

[153] Barbarestani SY, Jazi V, Mohebodini H, Ashayerizadeh A, Shabani A, Toghyani M. Effects of dietary lavender essential oil on growth performance, intestinal function, and antioxidant status of broiler chickens. Livest Sci. 2020;233:103958. 10.1016/j.livsci.2020.103958.

[154] Liu M, Zhou J, Li Y, Ding Y, Lian J, Dong Q, et al. Effects of dietary polyherbal mixtures on growth performance, antioxidt capacity, immune function and jejunal health of yellow-feathered broilers. Poult Sci. 2023;102(7):102714. 10.1016/j.psj.2023.102714. 10.1016/j.psj.2023.102714 PMC10196799 37172360

[155] Ghorbani MR, Bojarpur M, Mayahi M, Fayazi J, Fatemitabatabaei R, Tabatabaei S, et al. Effects of purslane extract on performance, immunity responses and cecal microbial population of broiler chickens. Span J Agr Res. 2014;12(4):1094–8. 10.5424/sjar/2014124-5483.

[156] Zhao W, Chen Y, Tian Y, Wang Y, Du J, Ye X, et al. Dietary supplementation with *Dendrobium officinale* leaves improves growth, antioxidant status, immune function, and gut health in broilers. Front Microbiol. 2023;14:1255894. 10.3389/fmicb.2023.1255894. 37789853 10.3389/fmicb.2023.1255894 PMC10544969

[157] Ke Y, Zhan L, Lu T, Zhou C, Chen X, Dong Y, et al. Polysaccharides of *Dendrobium officinale* Kimura & Migo leaves protect against ethanol-induced gastric mucosal injury via the AMPK/mTOR signaling pathway in vitro and vivo. Front Pharmacol. 2020;11:526349. 10.3389/fphar.2020.526349. 10.3389/fphar.2020.526349 PMC7686799 33262700

[158] Sun Y, Liu J, Wan L, Wang F, Zhang XJ, Qi YJ. Improving effects of *Astragalus polysaccharides* on cardiac function via Keap1/Nrf2-ARE signal pathway in adjuvant arthritis rats. Chin Herb Med. 2016;8(2):143–53. 10.1016/S1674-6384(16)60024-2.

[159] Yuan P, Ren X, Niu J, Liu Y, Huang L, Jiang S, et al. Effects of dietary *Galla Chinensis* Tannin supplementation on antioxidant capacity and intestinal microbiota composition in Broilers. Agriculture. 2023;13(9):1780. 10.3390/agriculture13091780.

[160] Cheng Y, Liu S, Wang F, Wang T, Yin L, Chen J, et al. Effects of dietary *Terminalia chebula* extract on growth performance, immune function, antioxidant capacity, and intestinal health of broilers. Animals. 2024;14(5):746. 10.3390/ani14050746. 10.3390/ani14050746 PMC10931075 38473130

[161] Liu WC, Guo Y, Zhao Z-H, Jha R, Balasubramanian B. Algae-derived polysaccharides promote growth performance by improving antioxidant capacity and intestinal barrier function in broiler chickens. Front Vet Sci. 2020;7:601336. 10.3389/fvets.2020.601336. 10.3389/fvets.2020.601336 PMC7738339 33344535

[162] Laurienzo P. Marine polysaccharides in pharmaceutical applications: an overview. Mar Drugs. 2010;8(9):2435–65. 10.3390/md8092435. 20948899 10.3390/md8092435 PMC2953395

[163] Abolfathi M, Tabeidian S, Shahraki AF, Tabatabaei S, Habibian M. Effects of ethanol extract of elecampane (*Inula helenium* L.) rhizome on growth performance, diet digestibility, gut health, and antioxidant status in broiler chickens. Livest Sci. 2019;223:68–75. 10.1016/j.livsci.2019.03.006. 10.1080/1745039X.2019.1581027 30821191

[164] Amer SA, Al-Khalaifah HS, Gouda A, Osman A, Goda NI, Mohammed HA, et al. Potential effects of anthocyanin-rich roselle *(Hibiscus sabdariffa *L.) extract on the growth, intestinal histomorphology, blood biochemical parameters, and the immune status of broiler chickens. Antioxidants. 2022;11(3):544. 10.3390/antiox11030544. 10.3390/antiox11030544 PMC8944722 35326194

[165] Seo JY, Park J, Kim HJ, Lee IA, Lim JS, Lim SS, et al. Isoalantolactone from *Inula helenium* caused Nrf2-mediated induction of detoxifying enzymes. J Med Food. 2009;12(5):1038–45. 10.1089/jmf.2009.0072. 19857067 10.1089/jmf.2009.0072

[166] Surai P. Polyphenol compounds in the chicken/animal diet: from the past to the future. J Anim Physiol Anim Nutr. 2014;98(1):19–31. 10.1111/jpn.12070. 10.1111/jpn.12070 23527581

[167] Mahfuz S, Shang Q, Piao X. Phenolic compounds as natural feed additives in poultry and swine diets: a review. J Anim Sci Biotechnol. 2021;12:48. 10.1186/s40104-021-00565-3. 10.1186/s40104-021-00565-3 PMC8025492 33823919

[168] Zhong Y, Li L, Chen W, Xing D, Wu X. Effects of *Ilicis chinensis *folium extract supplementation on growth performance, serum parameters, intestinal morphology, and antioxidant capacity of broiler chickens. BMC Vet Res. 2023;19:94. 10.1186/s12917-023-03667-4. 10.1186/s12917-023-03667-4 PMC10373348 37496032

[169] Jabir MS, Taha A, Sahib U. Antioxidant activity of Linalool. Eng Technol J. 2018;36:64–7. 10.30684/etj.36.1B.11.

[170] Liao PC, Lai MH, Hsu KP, Kuo YH, Chen J, Tsai MC, et al. Identification of β-sitosterol as in vitro anti-inflammatory constituent in *Moringa oleifera*. J Agric Food Chem. 2018;66(41):10748–59. 10.1021/acs.jafc.8b04555skong. 10.1021/acs.jafc.8b04555 30280897

[171] Nouri A, Heibati F, Heidarian E. Gallic acid exerts anti-inflammatory, anti-oxidative stress, and nephroprotective effects against paraquat-induced renal injury in male rats. Naunyn Schmiedeberg's Arch Pharmacol. 2021;394:1–9. 10.1007/s00210-020-01931-0. 32734364 10.1007/s00210-020-01931-0

[172] Yang YF, Zhao L, Shao YX, Liao XD, Zhang LY, Lin L, et al. Effects of dietary graded levels of cinnamon essential oil and its combination with bamboo leaf flavonoid on immune function, antioxidative ability and intestinal microbiota of broilers. J Integr Agr. 2019;18(9):2123–32. 10.1016/S2095-3119(19)62566-9.

[173] Li L, Chen X, Zhang K, Tian G, Ding X, Bai S, et al. Effects of thymol and carvacrol eutectic on growth performance, serum biochemical parameters, and intestinal health in broiler chickens. Animals. 2023;13(13):2242. 10.3390/ani13132242. 37444040 10.3390/ani13132242 PMC10340057

[174] Ji Y, Liu X, Lv H, Guo Y, Nie W. Effects of *Lonicerae flos* and turmeric extracts on growth performance and intestinal health of yellow-feathered broilers. Poult Sci. 2024;103(4):103488. 10.1016/j.psj.2024.103488. 10.1016/j.psj.2024.103488 PMC10869291 38335669

[175] Liu S, Wang K, Lin S, Zhang Z, Cheng M, Hu S, et al. Comparison of the effects between Tannins extracted from different natural plants on growth performance, antioxidant capacity, immunity, and intestinal flora of broiler chickens. Antioxidants. 2023;12(2):441. 10.3390/antiox12020441. 10.3390/antiox12020441 PMC9952188 36829999

[176] Zhou X, Li S, Jiang Y, Deng J, Yang C, Kang L, et al. Use of fermented Chinese medicine residues as a feed additive and effects on growth performance, meat quality, and intestinal health of broilers. Front Vet Sci. 2023;10:1157935. 10.3389/fvets.2023.1157935. 10.3389/fvets.2023.1157935 PMC10086232 37056232

[177] Mohammed HA, Abdelwahab MF, El-Ghaly E-SM, Ragab EA. Phytochemical characterization, in vitro anti-inflammatory, anti-diabetic, and cytotoxic activities of the edible aromatic plant; *Pulicaria jaubertii*. Molecules. 2021;26(1):203. 10.3390/molecules26010203. 10.3390/molecules26010203 PMC7796184 33401558

[178] Zhang Y, Li XY, Zhang BS, Ren LN, Lu YP, Tang JW, et al. In vivo antiviral effect of plant essential oils against avian infectious bronchitis virus. BMC Vet Res. 2022;18:90. 10.1186/s12917-022-03183-x. 10.1186/s12917-022-03183-x PMC8899001 35255906

[179] Qiao JY, Li HW, Liu FG, Li YC, Tian S, Cao LH, et al. Effects of *Portulaca oleracea* extract on acute alcoholic liver injury of rats. Molecules. 2019;24(16):2887. 10.3390/molecules24162887. 10.3390/molecules24162887 PMC6720614 31398934

[180] Yin X, Ding P, Xiao J, Yang Y, Song Z, He X, et al. Effects of *Galla chinensis* extract on growth performance, carcass traits, serum antioxidation, immune function, and gut microbiota of broilers. Front Anim Sci. 2022;3:880237. 10.3389/fanim.2022.880237.

[181] Niu X, Fan T, Li W, Xing W, Huang H. The anti-inflammatory effects of sanguinarine and its modulation of inflammatory mediators from peritoneal macrophages. Eur J Pharmacol. 2012;689(1–3):262–9. 10.1016/j.ejphar.2012.05.039. 22705062 10.1016/j.ejphar.2012.05.039

[182] Liu ZY, Wang XL, Ou SQ, Hou DX, He JH. Sanguinarine modulate gut microbiome and intestinal morphology to enhance growth performance in broilers. PLoS ONE. 2020;15(6):e0234920. 10.1371/journal.pone.0234920. 10.1371/journal.pone.0234920 PMC7304598 32559224

[183] Yang L, Chen L, Zheng K, Ma YJ, He RX, Arowolo MA, et al. Effects of fenugreek seed extracts on growth performance and intestinal health of broilers. Poult Sci. 2022;101(7):101939. 10.1016/j.psj.2022.101939. 10.1016/j.psj.2022.101939 PMC9194860 35691048

[184] Sharma M, Dinh T, Adhikari P. Production performance, egg quality, and small intestine histomorphology of the laying hens supplemented with phytogenic feed additive. J Appl Poult Res. 2020;29(2):362–71. 10.1016/j.japr.2019.12.001.

[185] Farahat M, Ibrahim D, Kishawy A, Abdallah H, Hernandez-Santana A, Attia G. Effect of cereal type and plant extract addition on the growth performance, intestinal morphology, caecal microflora, and gut barriers gene expression of broiler chickens. Animal. 2021;15(3):100056. 10.1016/j.animal.2020.100056. 10.1016/j.animal.2020.100056 33573933

[186] Liu S, Song M, Yun W, Lee J, Lee C, Kwak W, et al. Effects of oral administration of different dosages of carvacrol essential oils on intestinal barrier function in broilers. J Anim Physiol Anim Nutr. 2018;102(5):1257–65. 10.1111/jpn.12944. 10.1111/jpn.12944 29968943

[187] Cheng C, Wei H, Xu C, Xie X, Jiang S, Peng J. Maternal soluble fiber diet during pregnancy changes the intestinal microbiota, improves growth performance, and reduces intestinal permeability in piglets. Appl Environ Microb. 2018;84:e01047–18. 10.1128/AEM.01047-18. 10.1128/AEM.01047-18 PMC6102992 29959248

[188] Amer SA, Tolba SA, AlSadek DM, Abdel Fattah DM, Hassan AM, Metwally AE. Effect of supplemental glycerol monolaurate and oregano essential oil blend on the growth performance, intestinal morphology, and amino acid digestibility of broiler chickens. BMC Vet Res. 2021;17:312. 10.1186/s12917-021-03022-5. 10.1186/s12917-021-03022-5 PMC8467238 34563182

[189] Rusdi R, Hasanuddin A, Mulyati M, Fatmawati F. Effect of coconut husk extract on broiler chicken performance, pH and microbial composition of digesta, and small intestine histomorphology. J Indonesian Trop Anim Agric. 2022;47(2):119–27. 10.14710/jitaa.47.2.119-127.

[190] Sittiya J, Chimtong S, Sriwarcharameta P. Effects of crude oligosaccharide extract from agricultural by-products on the performance and gut development of broilers. Anim Biosci. 2023;36(6):891–8. 10.5713/ab.22.0338. 36634649 10.5713/ab.22.0338 PMC10164534

[191] Hao Y, Li D, Piao X, Piao X. *Forsythia suspensa* extract alleviates hypersensitivity induced by soybean β-conglycinin in weaned piglets. J Ethnopharmacol. 2010;128(2):412–18. 10.1016/j.jep.2010.01.035. 20083183 10.1016/j.jep.2010.01.035

[192] Guo S, Liu L, Lei J, Qu X, He C, Tang S, et al. Modulation of intestinal morphology and microbiota by dietary *Macleaya cordata* extract supplementation in Xuefeng Black-boned chicken. Animal. 2021;15(12):100399. 10.1016/j.animal.2021.100399. 34768172 10.1016/j.animal.2021.100399

[193] Amer SA, Mohamed WA, Gharib HS, Al-Gabri NA, Gouda A, Elabbasy MT, et al. Changes in the growth, ileal digestibility, intestinal histology, behavior, fatty acid composition of the breast muscles, and blood biochemical parameters of broiler chickens by dietary inclusion of safflower oil and vitamin C. BMC Vet Res. 2021;17:68. 10.1186/s12917-021-02773-5. 33541348 10.1186/s12917-021-02773-5 PMC7863266

[194] Zhang C, Li W, Chen L, Chen Z, Wang X, Xu Q, et al. Oregano oil combined with *Macleaya cordata* oral solution improves the growth performance and immune response of broilers. Animals. 2022;12(18):2480. 10.3390/ani12182480. 10.3390/ani12182480 PMC9495209 36139338

[195] Adewole DI, Oladokun S, Santin E. Effect of organic acids-essential oils blend and oat fiber combination on broiler chicken growth performance, blood parameters, and intestinal health. Anim Nutr. 2021;7(4):1039–51. 10.1016/j.aninu.2021.02.001. 34738034 10.1016/j.aninu.2021.02.001 PMC8546314

[196] Bahadori MM, Rezaeipour V, Abdullahpour R, Irani M. Effects of sesame meal bioactive peptides, individually or in combination with a mixture of essential oils, on growth performance, carcass, jejunal morphology, and microbial composition of broiler chickens. Trop Anim Health Prod. 2022;54(4):2351–8. 10.1007/s11250-022-03232-5. 10.1007/s11250-022-03232-5 35859053

[197] Parmar A, Patel V, Padheriya Y, Raval A, Rathwa S, Patel S. Effect of dietary supplementation of vegetable oil and quercetin on haematological indices and gut attributes in broiler chickens. Indian J Anim Health. 2022;61(1):139–46. 10.36062/ijah.2022.03122.

[198] Xue F, Shi L, Li Y, Ni A, Ma H, Sun Y, et al. Effects of replacing dietary aureomycin with a combination of plant essential oils on production performance and gastrointestinal health of broilers. Poult Sci. 2020;99(9):4521–9. 10.1016/j.psj.2020.05.030. 32867996 10.1016/j.psj.2020.05.030 PMC7598001

[199] Zhu N, Wang J, Yu L, Zhang Q, Chen K, Liu B. Modulation of growth performance and intestinal microbiota in chickens fed plant extracts or virginiamycin. Front Microbiol. 2019;10:1333. 10.3389/fmicb.2019.0133. 10.3389/fmicb.2019.01333 PMC6591263 31275268

[200] Sigolo S, Milis C, Dousti M, Jahandideh E, Jalali A, Mirzaei N, et al. Effects of different plant extracts at various dietary levels on growth performance, carcass traits, blood serum parameters, immune response and ileal microflora of Ross broiler chickens. Ital J Anim Sci. 2021;20(1):359–71. 10.1080/1828051X.2021.1883485.

[201] Zhang J, Yu H, Zhang H, Zhao Q, Si W, Qin Y, et al. Dietary *Epimedium* extract supplementation improves intestinal functions and alters gut microbiota in broilers. J Anim Sci Biotechnol. 2023;14:14. 10.1186/s40104-022-00812-1. 10.1186/s40104-022-00812-1 PMC9847172 36653873

[202] Islam R, Sultana N, Bhakta S, Haque Z, Hasan A, Siddique MP, et al. Modulation of growth performance, gut morphometry, and cecal microbiota in broilers by clove (*Syzygium aromaticum*) and tulsi (*Ocimum sanctum*) supplementation. Poult Sci. 2023;102(1):102266. 10.1016/j.psj.2022.102266. 10.1016/j.psj.2022.102266 PMC9660731 36370662

[203] Scalbert A. Antimicrobial properties of tannins. Phytochemistry. 1991;30(12):3875–83. 10.1016/0031-9422(91)83426-L.

[204] Hossain MM, Cho SB, Kang D-K, Nguyen QT, Kim IH. Comparative effects of dietary herbal mixture or guanidinoacetic acid supplementation on growth performance, cecal microbiota, blood profile, excreta gas emission, and meat quality in Hanhyup-3-ho chicken. Poult Sci. 2024;103(4):103553. 10.1016/j.psj.2024.103553. 10.1016/j.psj.2024.103553 PMC10907848 38417333

[205] Islam MR, Lepp D, Godfrey DV, Orban S, Ross K, Delaquis P, et al. Effects of wild blueberry (*Vaccinium angustifolium*) pomace feeding on gut microbiota and blood metabolites in free-range pastured broiler chickens. Poult Sci. 2019;98(9):3739–55. 10.3382/ps/pez062. 30918964 10.3382/ps/pez062

[206] Zhang Y, Liu Y, Li J, Xing T, Jiang Y, Zhang L, et al. Dietary resistant starch modifies the composition and function of caecal microbiota of broilers. J Sci Food Agr. 2020;100(3):1274–84. 10.1002/jsfa.10139. 31721238 10.1002/jsfa.10139

[207] Kong C, Gao R, Yan X, Huang L, Qin H. Probiotics improve gut microbiota dysbiosis in obese mice fed a high-fat or high-sucrose diet. Nutrition. 2019;60:175–84. 10.1016/j.nut.2018.10.002. 10.1016/j.nut.2018.10.002 30611080

[208] Zhu C, Huang K, Bai Y, Feng X, Gong L, Wei C, et al. Dietary supplementation with berberine improves growth performance and modulates the composition and function of cecal microbiota in yellow-feathered broilers. Poult Sci. 2021;100(2):1034–48. 10.1016/j.psj.2020.10.071. 33518062 10.1016/j.psj.2020.10.071 PMC7858044

[209] Shi Z, Qiu Y, Wang J, Fang Y, Zhang Y, Chen H, et al. Dysbiosis of gut microbiota in patients with neuromyelitis optica spectrum disorders: a cross sectional study. J Neuroimmunol. 2020;339:577126. 10.1016/j.jneuroim.2019.577126. 10.1016/j.jneuroim.2019.577126 31841737

[210] Huang T, Wang X, Yang Q, Peng S, Peng M. Effects of dietary supplementation with *Ampelopsis grossedentata* extract on production performance and body health of hens. Trop Anim Health Pro. 2022;54(1):451–14. 10.1007/s11250-022-03044-7. 10.1007/s11250-022-03044-7 35015154

[211] Zhou Y, Chen L, Sun G, Li Y, Huang R. Alterations in the gut microbiota of patients with silica-induced pulmonary fibrosis. J Occup Med Toxicol. 2019;14:5. 10.1186/s12995-019-0225-1. 10.1186/s12995-019-0225-1 PMC6399897 30867671

[212] Gandomi H, Abbaszadeh S, Rahimikia E, Shariatifar N. Volatile organic compound from *Pulicaria gnaphalodes* and the antibacterial and antifungal properties of its essential oil and aqueous, ethanolic and methanolic extracts. J Food Process Preserv. 2015;39(6):2129–34. 10.1111/jfpp.12456.

[213] Hozoorbakhsh F, Esfahan BN, Moghim S, Asghari G. Evaluation of the effect of *Pulicaria gnaphalodes* and *Perovskia abrotanoides* essential oil extracts against Mycobacterium tuberculosis strains. Adv Biomed Res 5:128–1340. 10.4103/2277-9175.180991 10.4103/2277-9175.180991 PMC4863401 27195252

[214] Kollanoor-Johny A, Darre MJ, Donoghue AM, Donoghue DJ, Venkitanarayanan K. Antibacterial effect of *trans*-cinnamaldehyde, eugenol, carvacrol, and thymol on *Salmonella *Enteritidis and *Campylobacter jejuni* in chicken cecal contents in vitro. J Appl Poult Res. 19:237–244. 10.3382/japr.2010-00181.

[215] Wang Y, Zhao H, Lin C, Ren J, Zhang S. Forsythiaside A exhibits anti-inflammatory effects in LPS-stimulated BV2 microglia cells through activation of Nrf2/HO-1 signaling pathway. Neurochem Res. 2016;41(4):659–65. 10.1007/s11064-015-1731-x. 26498935 10.1007/s11064-015-1731-x

[216] Martin-Gallausiaux C, Béguet-Crespel F, Marinelli L, Jamet A, Ledue F, Blottière HM, et al. Butyrate produced by gut commensal bacteria activates TGF-beta1 expression through the transcription factor SP1 in human intestinal epithelial cells. Sci Rep. 2018;8:9742. 10.1038/s41598-018-28048-y. 29950699 10.1038/s41598-018-28048-y PMC6021401

[217] Cabuk M, Alcicek A, Bozkurt M, Imre N, editors. Antimicrobial properties of the essential oils isolated from aromatic plants and using possibility as alternative feed additives. In National Animal Nutrition Congress. 2003;18(20):184–7.

[218] Ghanima MMA, Alagawany M, Abd El-Hack ME, Taha A, Elnesr SS, Ajarem J, et al. Consequences of various housing systems and dietary supplementation of thymol, carvacrol, and euganol on performance, egg quality, blood chemistry, and antioxidant parameters. Poult Sci. 2020;99(9):4384–97. 10.1016/j.psj.2020.05.028. 10.1016/j.psj.2020.05.028 PMC7598024 32867982

[219] Mansoub NH. Comparison of effects of using nettle (*Urtica dioica*) and probiotic on performance and serum composition of broiler chickens. Global Vet. 2011;6(3):247–50.

[220] Toaha SM, Mollah BR, Ahammad MU. Use of dietary fenugreek (*Trigonella foenum-graecum *L.) seed for the production of safe broiler lean meat. Res Agric Livest Fisheries. 2016;3(2):305–14. 10.3329/ralf.v3i2.29356.

[221] Cao S, Liu M, Han Y, Li S, Zhu X, Li D, et al. Effects of saponins on lipid metabolism: the gut–liver axis plays a key role. Nutrients. 2024;16:1514. 10.3390/nu16101514. 10.3390/nu16101514 PMC11124185 38794751

[222] Gholami-Ahangaran M, Haj‐Salehi M, Ahmadi‐Dastgerdi A, Zokaei M. The advantages and synergistic effects of Gunnera (*Gundelia tournefortii *L.) extract and protexin in chicken production. Vet Med Sci. 2021;7(6):2374–80. 10.1002/vms3.624. 10.1002/vms3.624 PMC8604120 34538006

[223] Rashid Z, Mirani ZA, Zehra S, Gilani SMH, Ashraf A, Azhar A, et al. Enhanced modulation of gut microbial dynamics affecting body weight in birds triggered by natural growth promoters administered in conventional feed. Saudi J Biol Sci. 2020;27(10):2747–55. 10.1016/j.sjbs.2020.06.027. 10.1016/j.sjbs.2020.06.027 PMC7499368 32994734

[224] Song Z, Xie K, Zhang Y, Xie Q, He X, Zhang H. Effects of dietary ginsenoside Rg1 supplementation on growth performance, gut health, and serum immunity in broiler chickens. Front Nutr. 2021;8:705279. 10.3389/fnut.2021.705279. 34912836 10.3389/fnut.2021.705279 PMC8667319

[225] Ghasemian SO, Gholami-Ahangaran M, Pourmahdi O, Ahmadi-Dastgerdi A. Dietary supplementation of protexin and artichoke extract for modulating growth performance and oxidative stress in broilers. Ankara Univ Vet Fak. 2022;69(3):281–8. 10.33988/auvfd.833094.

[226] Badiri R, Saber SN. Effects of dietary oregano essential oil on growth performance, carcass parameters and some blood parameters in Japanese male quail. Int J Pure App Biosci. 2016;4(5):17–22. 10.18782/2320-7051.2397.

[227] Hosseini S, Meimandipour A. Feeding broilers with thyme essential oil loaded in chitosan nanoparticles: an efficient strategy for successful delivery. Brit Poult Sci. 2018;59(6):669–78. 10.1080/00071668.2018.1521511. 30196710 10.1080/00071668.2018.1521511

[228] Windisch W, Schedle K, Plitzner C, Kroismayr A. Use of phytogenic products as feed additives for swine and poultry. J Anim Sci. 2008;86(14):140–8. 10.2527/jas.2007-0459. 10.2527/jas.2007-0459 18073277

[229] Provenza FD. Postingestive feedback as an elementary determinant of food preference and intake in ruminants. J Range Mgt. 1995;48(1):2–17. 10.2307/4002498.

[230] Smulikowska S, Pastuszewska B, Swiech E, Ochtabinska A, Mieczkowska A, Nguyen V, et al. Tannin content affects negatively nutritive value of pea for monogastrics. J Anim Feed Sci. 2001;10(3):511–24. 10.22358/jafs/68004/2001.

[231] Lupia C, Castagna F, Bava R, Naturale MD, Zicarelli L, Marrelli M, et al. Use of essential oils to counteract the phenomena of antimicrobial resistance in livestock species. Antibiotics. 2024;13:1631–26. 10.3390/antibiotics13020163. 10.3390/antibiotics13020163 PMC10885947 38391549

[232] Chhetri S, Arora S, Parcha V, Kumar D, Rawat DS. Nano encapsulation of an essential oil transpire the therapeutic approach. Curr Pharm Biotechnol. 2024. 10.2174/0113892010291682240423095306. 38738728 10.2174/0113892010291682240423095306

[233] Huang P, Zhang Y, Xiao K, Jiang F, Wang H, Tang D, et al. The chicken gut metagenome and the modulatory effects of plant-derived benzylisoquinoline alkaloids. Microbiome. 2018;6:211. 10.1186/s40168-018-0590-5. 30482240 10.1186/s40168-018-0590-5 PMC6260706

[234] Sun H, Wang N, Cang Z, Zhu C, Zhao L, Nie X, et al. Modulation of microbiota-gut-brain axis by berberine resulting in improved metabolic status in high-fat diet-fed rats. Obes Facts. 2017;9(6):365–78. 10.1159/000449507. 10.1159/000449507 PMC5644798 27898425

[235] Heger M, van Golen RF, Broekgaarden M, Michel MC. The molecular basis for the pharmacokinetics and pharmacodynamics of curcumin and its metabolites in relation to cancer. Pharmacol Rev. 2014;66(1):222–307. 10.1124/pr.110.004044. 24368738 10.1124/pr.110.004044

[236] Das Q, Islam MR, Lepp D, Tang J, Yin X, Mats L, et al. Gut microbiota, blood metabolites, and spleen immunity in broiler chickens fed berry pomaces and phenolic-enriched extractives. Front Vet Sci. 2020;7:526484. 10.3389/fvets.2020.00150. 10.3389/fvets.2020.00150 PMC7188780 33134328

[237] Patra AK. An overview of antimicrobial properties of different classes of phytochemicals. In: Patra A, editor. Diet Phytochemicals Microbes. Dordrecht: Springer; 2012. p. 1–32. 10.1007/978-94-007-3926-0_1.

[238] Trombetta D, Castelli F, Sarpietro MG, Venuti V, Cristani M, Daniele C, et al. Mechanisms of antibacterial action of three monoterpenes. Antimicrob Agents Ch. 2005;49(6):2474–8. 10.1128/AAC.49.6.2474-2478.2005. 10.1128/AAC.49.6.2474-2478.2005 PMC1140516 15917549

[239] Devi KP, Nisha SA, Sakthivel R, Pandian SK. Eugenol (an essential oil of clove) acts as an antibacterial agent against *Salmonella typhi* by disrupting the cellular membrane. J Ethnopharmacol. 2010;130(1):107–15. 10.1016/j.jep.2010.04.025. 20435121 10.1016/j.jep.2010.04.025

[240] Chowdhury S, Mandal GP, Patra AK, Kumar P, Samanta I, Pradhan S, et al. Different essential oils in diets of broiler chickens: 2. Gut microbes and morphology, immune response, and some blood profile and antioxidant enzymes. Anim Feed Sci Tech. 2018;236:39–47. 10.1016/j.anifeedsci.2017.12.003.

[241] Daniel AN, Sartoretto SM, Schmidt G, Caparroz-Assef SM, Bersani-Amado CA, Cuman RKN. Anti-inflammatory and antinociceptive activities a of eugenol essential oil in experimental animal models. Rev Bras Farmacogn. 2009;19:212–7. 10.1590/S0102-695X2009000200006.

[242] Kreydiyyeh S, Usta J, Copti R. Effect of cinnamon, clove and some of their constituents on the Na^+^-K^+^-ATPase activity and alanine absorption in the rat jejunum. Food Chem Toxicol. 2000;38(9):755–62. 10.1016/s0278-6915(00)00073-9. 10930696 10.1016/s0278-6915(00)00073-9

[243] Gao YY, Zhang XL, Xu LH, Peng H, Wang CK, Bi YZ. Encapsulated blends of essential oils and organic acids improved performance, intestinal morphology, cecal microflora, and jejunal enzyme activity of broilers. Czech J Anim Sci. 2019;64(5):189–98. 10.17221/172/2018-CJAS.

[244] Huang Q, Liu X, Zhao G, Hu T, Wang Y. Potential and challenges of tannins as an alternative to in-feed antibiotics for farm animal production. Anim Nutr. 2018;4(2):137–50. 10.1016/j.aninu.2017.09.004. 10.1016/j.aninu.2017.09.004 PMC6104569 30140753

[245] Starčević K, Krstulović L, Brozić D, Maurić M, Stojević Z, Mikulec Ž, et al. Production performance, meat composition and oxidative susceptibility in broiler chicken fed with different phenolic compounds. J Sci Food Agr. 2015;95(6):1172–8. 10.1002/jsfa.6805. 10.1002/jsfa.6805 24995966

[246] Ren X, Yuan P, Niu J, Liu Y, Li Y, Huang L, et al. Effects of dietary supplementation with microencapsulated *Galla chinensis* tannins on growth performance, antioxidant capacity, and lipid metabolism of young broiler chickens. Front Vet Sci. 2023;10:1259142. 10.3389/fvets.2023.1259142. 10.3389/fvets.2023.1259142 PMC10637619 37954663

[247] Niu J, Wang Q, Jing C, Liu Y, Liu H, Jiao N, et al. Dietary *Galla Chinensis* tannic acid supplementation in the diets improves growth performance, immune function and liver health status of broiler chicken. Front Vet Sci. 2022;9:1024430. 10.3389/fvets.2022.1024430. 10.3389/fvets.2022.1024430 PMC9614106 36311675

[248] Maurya A, Singh VK, Das S, Prasad J, Kedia A, Upadhyay N, et al. Essential oil nanoemulsion as eco-friendly and safe preservative: Bioefficacy against microbial food deterioration and toxin secretion, mode of action, and future opportunities. Front Microbiol. 2021;12:751062. 10.3389/fmicb.2021.751062. 10.3389/fmicb.2021.751062 PMC8667777 34912311

