# Supplementary material for: Phytobiotics in poultry: revolutionizing broiler chicken nutrition with plant-derived gut health enhancers
Source: J Anim Sci Biotechnol. 2024 Dec 9;15:169. doi: 10.1186/s40104-024-01101-9 (PMC11626766; doi:10.1186/s40104-024-01101-9)
Supplement: Supplementary file 1 — Additional file 1. Effects of various phytobiotics on physiological responses and growth performance of broiler birds. [file 40104_2024_1101_MOESM1_ESM.docx]

| Additional file 1 Effects of various phytobiotics on physiological responses and growth performance of broiler birds | | | | | | | |  |
| --- | --- | --- | --- | --- | --- | --- | --- | --- |
| Breed and sex | **Age** | **Size** | **Days** | **Diets** | **Overall effects** | **Implications** | **References** |  |
| Ross 308 M | 1-day old | 200 | 42 | *Enteromorpha prolifera* polysaccharide at 400 mg/kg diet | Improved intestinal immune response, antioxidant function and villi development | Improved growth performance | [10] |  |
|  |  |  |  |  |  |  |  |  |
| Arbor acres M | 1-day old | 500 | 42 | Control, 50, 100, 200 or 400 mg/kg EO (Thymol, Carvacrol and Cinnamaldehyde) | Increased expression of nutrient transporters, sIgA level, and villi development | Improved weight gains significantly at 200 mg/kg inclusion level | [11] |  |
| Ross 308 F | 1-day old | 128 | 42 | Grape seed or fermented grape seed at 5 g/kg | Enhanced proliferation of beneficial microbes, and antioxidant function | Improved growth performance but no effect on carcass yield | [18] |  |
| Arbor acres Mixed sex | | 1-day old | 384 | 42 | Control, Antibiotics, Natural Oregano Essential oil (NOEO), and synthetic Essential oil (SEO) at 200 mg/kg respectively | NOEO increased intestinal antioxidant function, immunity, enzyme secretion, and villi morphology | NOEO significantly improved growth performance and feed efficiency | [19] |
| Qingyuan F | | 1-day old | 960 | 36 | Control, Virginiamycin (20 mg/kg), Oregano essential oil at 150 or 300 mg/kg diet | OEO improved gut microbiota balance, immunity, antioxidant and intestinal barrier function. | OEO improved growth performance | [20] |
| Ross 308 M | | 1-day old | 500 | 42 | Control, Enramycin at 50 mg/kg, OA (200 mg/kg), EO (150 mg/kg) and OA blend plus coated EO | The EO and OA groups improved nutrient digestibility and reduced pathogen colonization in the gut | The EO and OA groups improved growth performance and feed efficiency | [21] |
| Ross-708 M | | 1-day old | 432 | 42 | Antibiotics, Ginger root extract at 0.375%, 0.75%, 1.5% and 3% | 1.5 % increased immunity and suppressed pathogen proliferation | Growth performance was reduced with 3% while 1.5% was comparable to control | [22] |
| Arbor Acres M and F | | 1-day old | 840 | 42 | Antibiotics, Oregano aqueous extract at 400, 500, 600 and 700 mg/kg | 0AE improved villi morphology, intestinal immunity, abundance beneficial microbes and SCFAs level | OAE at 700 mg/kg significantly improved growth performance and feed efficiency | [23] |
| Ross 308 M | | 1-day old | 400 | 42 | *Enteromorpha* Polysaccharides (EP), 200 mg/kg, plus Yeast glycoprotein (YG), 200 mg/kg | Improved gut antioxidant function | Improved growth performance and modulated serum biochemical indices | [24] |
| Arbor acres M | | 1-day old | 324 | 42 | 80 mg/kg Zn from ZnSO4; 80 mg/kg Zn + 482 mg POS (pectin oligosaccharide) and 80 mg/kg Zn‐POS chelate | POS and Zn-POS enhanced synthesis of SCFAs, positively modulated duodenal and jejunal morphology, | Enhanced growth performance and antioxidant status | [25] |
| Ross 308 M | | 1-day old | 240 | 21 | Xylooligosaccharides (XOS; 100 mg/kg) and Gamma-irradiated *Astragalus polysaccharides* (600 mg/kg) | The combined form improved villi morphology, goblet cell number and Tight junction proteins | Enhanced weight gain and feed efficiency | [26] |
| Arbor acres M | | 1-day old | 360 | 42 | *Plotytarya strohilacea Sieb.et Zuce* tannin; 100, 400, 800 mg/kg diet | Improved jejunal morphology at 100 mg/kg inclusion | Improved growth performance | [29] |
| Ross 308 M | | 3-day old | 200 | 35 | Lavender (*Lavandula angustifolia L*.) essential oil; 0, 200, 400, 600 mg/kg | Reduced inflammatory response but below 600 mg/kg | Exerted no significant effect on growth performance | [31] |
| Ross 308 unsexed | | 1-day old | 288 | 42 | Basil oil: Free and microencapsulated at 500 ppm respectively | Enhanced jejunal morphology | Reduced FCR but no significant effect on weight gain | [33] |
| MB-202 M | | 1-day old | 144 | 35 | Microencapsulated turmeric by maltodextrin: 1, 2, 3 g/kg diet | Enhanced gut morphology, beneficial microbes count, haematology and antioxidant function at 3g/kg | Enhanced growth performance | [34] |
| Arbor acres M | | 1-day old | 480 | 42 | Antibiotics, 300 mg/kg *Astragalus membranaceus*, and 150 mg/kg *Glycyrrhiza uralencis* | ASP and GSP improved villi morphology, intestinal barrier function, immunity, and abundance of beneficial microbes | Improved growth performance, and antioxidant status | [56] |
| Ross 308 M | | 1-day old | 432 | 35 | Gum arabic powder (*Acacia senegal*) at 0.12%, 0.25%, 0.5%, 0.75% and 1.0% | Increased villi morphometrics and histometric values of the ileum | Improved weight gain and feed conversion ratio | [75] |
| Cobb 500 M | | 1-day old | 234 | 28 | Essential oil 400 mg/kg Plant essential oil, 30 mg/kg virginiamycin, control without additives | Improved abundance of beneficial microbiota | Improved weight gain | [81] |
| Ross 308 unsexed | | 1-day old | 180 | 35 | Control, Enramycin 10 mg/kg, synbiotics (3 × 10 (11) CFU/kg, EO at 100 mg/kg and EO + synbiotics | EO improved villi architecture, E0 +SYN increased gut health-related genes expression, SCFAs, and gut microbiota composition | The SYN and EO +SYN treatments increased weight gain | [84] |
| Ross 308 M | | 1-day old | 500 | 35 | Licorice extract (*Glycyrrhiza glabra*). 0.25, 0.5, 1 and 2 g/kg diet | Improved gut immunity, increased the expression of tight junction proteins, and reduced inflammation | Growth performance was enhanced significantly | [96] |
| Arbor Acres M | | 7-day old | 720 | 35 | Olive leaf extract at 0.1%-0.5% | Increased abundance of *Lactobacillus* and *Bifidobacterium,* decreased *E. coli* | Decreased growth performance but enhanced breast muscle meat | [99] |
| Cobb 500 M | | 1-day old | 800 | 42 | *Quillaja saponaria* and *Yucca* schidigera; OY biomass at 250 g/t | Improved jejunal morphology, maintained intestinal barrier function and nutrient digestibility | Enhanced feed efficiency and growth performance | [101] |
| Ross 308 M | | 1-day old | 300 | 42 | *Forepia subpinata* powder at 1%, 2%, 3% | Enhanced villi morphology and beneficial microbes count at 3% inclusion level | Improved growth performance and feed efficiency | [102] |
| Ross 308 M | | 1-day old | 240 | 35 | *Pulicaria Jauberti* powder at 0, 3, 6 and 9 g/kg | Regulated gut immunity and inflammatory response, increased beneficial gut microbes | Optimal growth performance at 3 g/kg | [103] |
| Ross 308 M | | 1-day old | 576 | 42 | *Pulicaria gnaphalodes* powder at 0.1%-0.3%, Antibiotics (0.05% bacitracin methylene disalicylate) | Plant powder enhanced villi morphometrics significantly at 0.3 % inclusion level | Improved weight gain and feed efficiency at 0.3% inclusion level | [104] |
| Ven Cobb 400 unsexed | | 1-day old | 240 | 42 | Chicory root (1.0%) and coriander seed powder (1.5%) | Improved villi morphology and nutrient utilization | Improved weight gain and growth performance | [105] |
| Ross 308 M | | 1-day old | 200 | 28 | Curcumin (200 mg/kg), *Pueria* Extract (200 mg/kg) and CUR + PE (200 + 200 mg/kg) | The CUR, CUR + PE improved intestinal antioxidant function, villi morphology and PE increased intestinal barrier function | PE + CUR improved feed efficiency | [108] |
| Cobb 500 M | | 1-day old | 400 | 42 | EO (Star Anise, rosemary, thyme and oregano), blend of *quilaja saponin* and EO at 25 mg/kg | Blend of saponin and EO improved villi morphology | Improved weight gain and feed conversion ratio | [122] |
| Arbor Acres M | | 1-day old | 480 | 42 | Wheat germ (powder), hops and grape seed extract as a mixture: 0.05%, 0.1% and 0.2% | The blend significantly reduced pathogens and increased *Lactobacillus* proliferation | 0.2 % inclusion level significantly improved growth performance at all phases | [129] |
| Ross 308 M | | 10-day old | 500 | 42 | E0 (thyme, peppermint, and Eucalyptus) at 50, 100, 150 and 200 ppm drinking water | 150 and 200 ppm enhanced villi morphometrics in the ileum | Improved weight gain, and FCR at 150 ppm inclusion level. | [133] |
| Yellow-Feather M | | 1-day old | 360 | 51 | Magnolol at 100-400 mg/kg | Enhanced jejunal antioxidant status and abundance of SCFA producers | 200 and 300 mg/kg improved weight gain and feed efficiency | [140] |
| Arbor acres M | | 1-day old | 240 | 21 | Beta sitosterol at 25, 50, 75, and 100 mg/kg | Reduced serum endotoxin level, increased villi development, and antioxidant enzymes. | Improved feed efficiency | [142] |
| Arbor acres M | | 1-day old | 96 | 42 | Ferulic acid at 80 mg/kg | Improved digestive enzymes secretions, antioxidant and immunity function | Improved growth performance | [143] |
| Cobb-500 M | | 1-day old | 180 | 42 | Licorice extract (*Glycyrrhiza glabra*). 0.4 or 0.8 g/L of water | 0.4 g enhanced villi morphometrics and increased the number of CD3^+^ in the duodenum and ileum | Enhanced growth performance | [144] |
| Cobb-500 M | | 1-day old | 480 | 21 | Encapsulated product (Capsicum blend with black pepper and ginger extract) at 250 ppm | Spicy blend enhanced ileal digestibility of nutrients | Spicy blend improved growth performance at early stage | [145] |
| Arbor Acres M | | 1-day old | 192 | 42 | *Forsythia suspensa* extract (FSE): 100 mg/kg, CTC, (basal diet + 75 mg/kg chlortetracycline) | FSE enhanced villi morphometrics of the duodenum, jejunum and ileum | Improved nutrient digestibility, which reduced the nitrogen excretion | [146] |
| White feathers M | | 1-day old | 144 | 50 | A blend (*Astragalus membranaceus* and *Codnopsis pilosula* extract) at 500 mg/kg | Improved intestinal immunity and barrier function, and altered gut microbial composition | Improved feed efficiency but growth performance was comparable to control | [147] |
| Yellow Feather M | | 1-day old | 480 | 56 | Yucca saponin (YSa), *Yucca schidigera* (YS), and *Quillaja Saponaria* (QS) extracts at 500 mg/kg each | The extracts improved immunity status, and SCFAs level | YS improved feed efficiency while all treatments improved growth performance | [148] |
| Arbor acres Unsexed | | 1-day old | 300 | 42 | Cinnamon oil at 500, 1,000 and 1,500 mg/kg | Decreased gut pathogens and increased lactic acid bacteria count | Improved growth performance and immunity status. | [151] |
| Yellow Feather M | | 1-day old | 720 | 48 | EO at 200, 400 and 600 mg/kg (Carrier: rice husk powder and silica) | Inhibited the growth and biofilm formation of pathogens, reduced intestinal permeability and enriched metabolic pathways | Improved growth performance and feed to gain ratio | [152] |
| Arbor acres M | | 1-day old | 480 | 42 | Control, Virginiamycin at 200 mg/kg, Lavender EO at 300 or 600 mg/kg | 600 mg/kg improved villi morphology and gut microbiota balance | Improved growth performance and feed efficiency for all stages | [153] |
| Yellow Feather M | | 1-day old | 270 | 42 | Antibiotics, Polyherbal mixture (5 herbs), at 1,000 mg/kg | Improved mucosal immunity, antioxidant function, intestinal barrier function and microbial diversity | Improved weight gain and feed-to gain ratio significantly | [154] |
| Zhejiang Xianju M | | 1-day old | 192 | 70 | *Dendrobium officinale* leaves at 0, 1%, 5% or 10% | Improved villi morphology, microbial composition, SCFAs synthesis and reduced inflammation | Improved growth performance and feed efficiency at 5 % level | [156] |
| Arbor Acres M | | 1-day old | 288 | 42 | *Galla chinesis* Tannins at 300 mg/kg | GCT increased gut beneficial microbes, and antioxidant capacity via upregulated expression of related genes | Growth performance was not evaluated. | [159] |
| Yellow Feather M | | 1-day old | 288 | 56 | *Terminalia chebula* extract at 0, 200, 400 and 600 mg/kg | Improved gut morphology, immunity status, SCFAs synthesis and microbial diversity | Improved growth performance and feed to gain ratio in a dose-dependent manner | [160] |
| Arbor acres M | | 1-day old | 396 | 35 | Algae -derived polysaccharides; *Enteromorpha* at 0, 1,000, 2,500, 4,000, 5,500, and 7,000 mg/kg | Improved villi morphology, antioxidant function, and reduced DAO-and D-LA level in the serum | Improved weight gain and feed efficiency | [161] |
| Ross 308 M | | 1-day old | 360 | 42 | Ethanol extract of elecampane (*Inula helenium* L.) rhizome at 250, 500 or 1000 mg/kg | Improved gut morphology and antioxidant function | Feed efficiency and weight were increased in a dose-dependent manner | [163] |
| Ross 308 Unsexed | | 1-day old | 250 | 35 | Anthocyanin-Rich Roselle (Hibiscus sabdariffa L.) extract at 50, 100, 200 and 400 mg/kg | Improved villi morphometrics at 100 mg/kg inclusion level | Dietary treatments had no effect on growth performance | [164] |
| Lohmann unsexed | | 1-day old | 360 | 42 | *Ilicis Chinesis folium* extract (powder) at 0, 250, 500, and 1000 mg/kg | Extract improved intestinal morphology and immunity status | Growth performance was significantly improved, 500 mg/kg recommended as optimal | [168] |
| Hubbard M | | 1-day old | 960 | 42 | Oral solutions of: Oregano EO, *macleaya cordata* extract (MCE), and Oregano EO + MCE at (125 mL/1,000 L) | Combined OEO and MCE improved gut morphology, immunity and reduced inflammatory response | Combined OEO and MCE improved growth performance significantly | [173] |
| Arbor Acres M | | 1-day old | 640 | 42 | Thymol and Carvacrol eucetic at 0, 30, 60 or 120 mg/kg | Improved intestinal morphology, immunity and intestinal barrier function | Optimal growth performance was attained with 300 mg/kg | [174] |
| Yellow feathered Unsexed | | 21-day old | 720 | 60 | *Loncirae flos* and turmeric extract at 0, 300, 500 g/t | Improved villi morphology, intestinal barrier function, immunity and microbial diversity | The LTE improved growth performance in a dose-dependent manner | [175] |
| Arbor acre M | | 1-day old | 576 | 42 | Combination of 100/200 mg of CEO (Cinnamon EO) and 16.7/33.3 mg of BLF (Bamboo leaf flavonoid)/kg | Reduced duodenal crypt depth | No effect on growth performance | [177] |
| Yellow Feather M | | 1-day old | 432 | 63 | *Galla chinesis* extract; 250, 500, 1000 or 2000 mg/kg | 250 mg/kg enhanced beneficial microbes and gut immunity | Improved growth performance | [181] |
| Yellow-Feather F | | 1-day old | 270 | 56 | Sanguinarine (from *Macleaya cordata*) at 0.7 mg/kg diet | Increased abundance of beneficial microbes, villi morphometrics and reduced inflammation in the jejunum | Improved growth performance at early phase | [183] |
| Yellow Feather M | | 1-day old | 240 | 56 | Antibiotics (30 mg/kg Zinc bacitracin), Fenugreek extract at 50 or 100 mg/kg | FSE enhanced villi morphology, gut immunity and beneficial microbes, and decreased inflammation | Improved performance and gut microbiota homeostasis | [184] |
| Ross 308 M | | 1-day old | 400 | 42 | Plant extract (curcuma, chamomile, licorice and olive leaf) at 500-1,000 mg/kg | Improved villi morphology and intestinal barrier function | Enhanced weight gain and reduced feed conversion ratio | [185] |
| Ross 308 M | | 3-day old | 240 | 35 | Blend of (OEO) oregano Essential oil and GLM (Glycerol monolaurate) at 0, 0.15%, 0.45%, and 0.75% | Improved villi morphology in all the intestinal segments, reduced goblet count in the ileum | 0.75 % increased the growth performance index | [188] |
| Arbor Acres M | | 1-day old | 250 | 42 | Antibiotics (40 ppm tetracycline),  Coconut husk extract (CHE) at 100, 400 and 700 ppm | CHE inhibited the growth of pathogens in the gut and increased villi development | The extract significantly improved performance indices | [189] |
| Ross 308 M | | 3-day old | 300 | 35 | Safflower oil; 5 and 10 g/kg diet | Increased villi morphometrics, mucosal thickness, and goblet cell count | Improved growth performance and amino acid digestibility | [194] |
| Ross 308 mixed sex | | 1-day old | 412 | 36 | Control, Antibiotic, protected organic acids and essential oil (OE) blend at 300 g/t, OE 300 g/t +3% oat hull | Improved jejunal morphology, goblet cell number, beneficial gut microbes but had no effect on SCFA synthesis | Improved gut health and could be used as replacement for antibiotics | [195] |
| Ross 308 M | | 1-day old | 250 | 42 | Sesame meal bioactive peptides at 3 g/ kg, and EO (savory and thyme) at 0.5 g/kg, antibiotics: 0.5 g/kg of Bacitracin | The combined form enhanced gut villi morphology and suppressed pathogens colonization | Improved weight gain and reduced feed conversion ratio | [196] |
| Ven Cobb 400 M | | 1-day old | 192 | 42 | Quercetin:1 g/kg, Vegetable oil: 10% | Had no effect on gut morphology | Quercetin enhanced haematological indices | [197] |
| Arbor acres M | | 1-day old | 720 | 42 | Control, Aureomycin, combined plant oil (Eucalyptus, carvacrol, cinnamyl aldehyde) at 100 g/ton | The combined plant oil improved gut morphology, bacterial diversity and abundance of probiotic bacteria. | All treatment improved growth performance and feed efficiency, comparably | [198] |
| Cobb 500 M | | 1-day old | 288 | 28 | Control without additive, Virginiamycin at 30 mg/kg, Plant extract oil at 200 or 400 mg/kg | PEO improved microbial composition and related metabolic functional pathways | Improved weight gain and feed-meat ratio at 400 mg/kg inclusion level | [199] |
| Ross 308 M | | 1-day old | 650 | 42 | Thyme, coriander, dill and common nettle extracts at 150, 300 and 450 mg/L | 150 mg /L of thyme extract enhanced proliferation of LAB in the ileum | 300 mg /L of thyme extract improved weight gain and feed efficiency | [200] |
| Arbor Acres M | | 1-day old | 720 | 42 | Epimedium extract at 100, 200, 400, or 800 mg/kg | Improved intestinal immunity and barrier function, SCFA synthesis and gut microbial composition | EM at 200 mg/kg improved growth performance | [201] |
| Cobb-500 Unsexed | | 1-day old | 60 | 28 | Clove powder and Tulsi extract via water at (0.5% +2%), (1% + 3%), and (1.5% + 4%) | Improved villi morphology, and proliferation of beneficial microbes | Significant improvement in weight gain with (1% + 3%) inclusion level | [202] |
| Korean Native chicken M and F | | 1-day old | 360 | 100 | Herbal mixture (Ginseng and Artichoke: 0.05%) and organic acid (guanidinoacetic acid: 0.06%) | Improved antioxidant capacity | 0.05% herbal mixture improved weight gain and feed efficiency | [204] |
| Yellow-Feather F | 1-day old | 360 | 63 | Berberine (BBR) at 250 mg, Control, and 200 mg/kg oxytetracycline calcium (antibiotics) | BBR supplementation increased Ileal microbial composition and diversity | Improved growth performance and feed to gain ratio | [208] |  |
| Chinese Yellow M | | 1-day old | 300 | 70 | Control, Bacitracin zinc 30 mg/kg, Encapsulated blend of EO and OA at 150, 200 or 250 mg/kg | Blend of EO and OA increased villi architecture, intestinal enzyme secretion, immunity status and balanced microflora | Blends of EO and OA increased weight gain and improved gain-feed ratio | [243] |
| Arbor acres M | | 1-day old | 280 | 42 | Green tea powder (GTP) at 1%, 0.0004% antibiotics+ 1% GTP | 1 % GP enhanced population of beneficial microbes and reduced pathogens colonization | Improved body weight gain and meat quality | [249] |
| Ven Cobb unsexed | | 1-day old | 192 | 42 | Phytogenic blend powder (*Aerva lanata*, *Piper betle*, *Cynodon dactylon*, and *Piper nigrum* at 1% and 2% | Improved jejunal villi height and ileal digestibility of organic matter | Improved growth performance and feed efficiency | [250] |
| Ross 308 unsexed | | 1-day old | 1500 | 35 | EO (oregano, rosemary, cinnamon chilli pepper extracts: 100 g/t), OA (1 kg/t), EO + OA (100 g:1 kg/t) | Improved villi height of the jejunum but no effect on crypt depth and villi width | Improved growth performance and FCR, but no effect on FI | [251] |
| Cobb-500 Unsexed | | 1-day old | 600 | 35 | Antibiotics, *Acaia Nilotica* extract at 0.1 %, 0.3 % and 0.5% | Extract improved intestinal morphology | The extract improved weight gain and feed efficiency significantly | [252] |
| Cobb-500 Unsexed | | 1-day old | 352 | 33 | *Piper aduncum*, *Morinda citrifolia,* and *Artocarpus altilis* leaves (0.005% each) and 0.01% EE of each leaves | 0.01 % *M. citrifolia* improved villi morphology and inhibited pathogen growth | 0.01 % *M. citrifolia* improved weight gain and feed efficiency at all phases | [253] |

**References**

249. Chen X, Zhu W, Liu X, Li T, Geng Z, Wan X. The growth performance, meat quality, and gut bacteria of broilers raised with or without antibiotics and green tea powder. J Appl Poult Res. 2019;28(3):712–21. https://doi.org/10.3382/japr/pfz02.

250. Oso AO, Suganthi RU, Reddy GM, Malik PK, Thirumalaisamy G, Awachat VB, et al. Effect of dietary supplementation with phytogenic blend on growth performance, apparent ileal digestibility of nutrients, intestinal morphology, and cecal microflora of broiler chickens. Poultry science, 2019;98(10):4755–66. http://dx.doi.org/10.3382/ps/pez191.

251. Iqbal H, Rahman A, Khanum S, Arshad M, Badar IH, Asif AR, et al. Effect of essential oil and organic acid on performance, gut health, bacterial count and serological parameters in broiler. Braz J Poult Sci. 2021;23(03):001–10. http://dx.doi.org/10.1590/1806-9061-2021-1443.

252. Zahid MU, Khalique A, Qaisrani SN, Ashraf M, Sheikh AA, Yaqoob MU. The effect of *Acacia nilotica* bark extract on growth performance, carcass characteristics, immune response, and intestinal morphology in broilers as an alternative to antibiotic growth promoter. Anim Biosci. 2023;36(7):1059–66. https://doi.org/10.5713/ab.22.0284.

253. Paredes-López DM, Robles-Huaynate RA, Soto-Vásquez MR, Perales-Camacho RA, Morales-Cauti SM, Beteta-Blas X, et al. Modulation of gut microbiota, and morphometry, blood profiles and performance of broiler chickens supplemented with *Piper aduncum*, *Morinda citrifolia*, and *Artocarpus altilis* leaves ethanolic extracts. Front Vet Sci. 2024;11:1286152. https://doi.org/10.3389/fvets.2024.1286152.
